# Stress, self-esteem and well-being among female health professionals: A randomized clinical trial on the impact of a self-care intervention mediated by the senses

**DOI:** 10.1371/journal.pone.0172455

**Published:** 2017-02-27

**Authors:** Eliseth Ribeiro Leão, Daniela Reis Dal Fabbro, Rebeca Barqueiro de Oliveira, Ingrid Ribeiro dos Santos, Elivane da Silva Victor, Rita Lacerda Aquarone, Cristiane Benvenuto Andrade, Vivian Finotti Ribeiro, Roselaine Coelho de Oliveira, Rosa Friedlander, Daniela Santos Ferreira

**Affiliations:** 1 Research Institute, Hospital Israelita Albert Einstein. São Paulo, Brazil; 2 University of Sao Paulo, Sao Paulo, Brazil; 3 Israeli Faculty of Heath Sciences Albert Einstein, Hospital Israelita Albert Einstein, Sao Paulo, Brazil; 4 Quality and Safety, Hospital Israelita Albert Einstein, Sao Paulo, Brazil; 5 Natura Cosméticos, Sao Paulo, Brazil; Mayo Clinic, UNITED STATES

## Abstract

**Background:**

Stress levels are evident among health professionals. However, there are few studies on sensory-based self-care aimed at stress management, self-esteem and subjective well-being in this group of professionals.

**Objective:**

To assess the impact of a self-care intervention mediated by the senses on the stress levels, self-esteem and well-being of health professionals in a hospital environment.

**Methods:**

A total of 93 health professionals participated in an unblinded clinical trial, randomized into four groups: 1) control (no intervention); 2) Monosensory—daily body moisturizing (DBM) with odorless cream; 3) Bisensory—DBM with scented cream; 4) Multisensory—DBM with scented cream associated with audiovisual material. Participants answered specific questionnaires to assess stress, self-esteem and well-being and cortisol samples were collected at baseline, 15 and 30 days following intervention, and at the 30-day follow-up.

**Results:**

Self-care was characterized as neglected, with most participants reporting inadequate hours of sleep (74%), irregular physical activity (68%), and inadequate nutrition (45%). Compared to the other groups, the Bisensory group had lower stress on all three assessments (p = 0.017; 0.012; 0.036), a life satisfaction 8% higher at follow-up than at baseline (95% CI: 2% to 15%, p = 0.016), a 10% increase in positive affect (95% CI: 2% to 19%, p = 0.011) and a 12% reduction in negative affect (95% CI: 3% to 21% less, p = 0.014) after 30 days. The Multisensory group showed improvement in self-esteem (p = 0.012) and reduced cortisol (p = 0.036) after 30 days of intervention. The control group showed no changes in the variables studied, except for cortisol: an increase at the 15-day evaluation (denoting higher risk for stress, p = 0.009) and a reduction at follow-up (p = 0.028), which was nevertheless within normal levels.

**Trial registration:**

Clinicaltrials.gov NCT02406755

## Introduction

The stress of health professionals has been the subject of scientific research due to its costs and deleterious effects on individuals and organizations. For over two decades, national and international studies have shown the potential negative effects of occupational stress on the health and well-being of health professionals, particularly in terms of low satisfaction [1], physical and psychological complaints [2] and absenteeism [3–4]. Women are particularly prone to problems related to occupational stress [5].

Stress is associated with the occurrence of adverse events, mainly regarding medication administration errors (in prescribing, dosing, dispensing, or administering drugs) [6]. Therefore, controlling stress is necessary not only to preserve workers health and well-being but also to ensure quality of care and safety for the patient. This reinforces the need for self-care among professionals to allow them to recharge their energy, eventually leading to better work performance.

Although stress levels are evident among health professionals, there are few intervention studies in the literature indicating effective measures of self-care for stress management for this segment of the population, or exploring the impact of stress on self-esteem and subjective well-being (understood in this study as life satisfaction and characterized by the predominance of positive affect).

Sensory experiences (tactile, olfactory and visual) have been studied in many clinical situations and they show a potential for promoting well-being and stress management [7]. However, interventions in hospitals involving the senses (together or in isolation) have been conducted mostly with patients and not with health professionals as the target population.

Therefore, this study aimed to evaluate the impact of a self-care intervention mediated by the senses on levels of stress, self-esteem and well-being among female health professionals working in a hospital setting.

## Methods

The protocol for this trial and supporting CONSORT checklist are available as supporting information; see **S1** and **S2 Texts**.

### Type of study

This was an open, controlled, randomized clinical trial, with a quantitative approach, performed at a large private philanthropic hospital in the city of São Paulo. The study (called H-Senses) was funded by Call of Science, Technology and Innovation—Natura Campus.

### Ethical and legal aspects

H-Senses Project—Self Care Mediated by the Senses was registered under clinicaltrials.gov identifier: NCT02406755 (October 27, 2014, **S3 Text**). The authors confirm that all related trials for this intervention are registered. Unfortunately due to administrative problems logging in clinical trials platform it was submitted late and after the inclusion of some participants in the study (July, 2014). However, we emphasize that the study recorded strictly follows the version approved by the Research Ethics Committee of the Hospital Israelita Albert Einstein in a regular meeting held on April 8, 2014 before the study start under n° CAAE: 26547314.3.0000.0071 (Letter of approval of the Research Ethics Committee presented in additional supplement **S4 Text**). In accordance with the Resolution 466/12 of the National Health Council, participants were informed about the content and purpose of the study and were included in the study after signing an informed consent.

### Study participants

The convenience sample consisted of female health professionals aged between 18 and 60 years old who worked in health care or health care-related areas. Exclusion criteria included relevant dermatological findings, women who worked at night or alternating shifts (due to the known chronobiological changes that could affect the outcomes in this study), as well as women who were pregnant or lactating. The target sample size was defined for convenience with no theoretical background, since data necessary for a pre-study sample size determination were not available in the previous literature in the way that was considered in this study. Nevertheless, anticipating potential losses in the follow-up, we intended to reach at least 100 participants for the total sample size, aiming to guarantee a good performance in the adjustment of a Generalized Estimating Equation (GEE model)[8].

**Fig 1:**
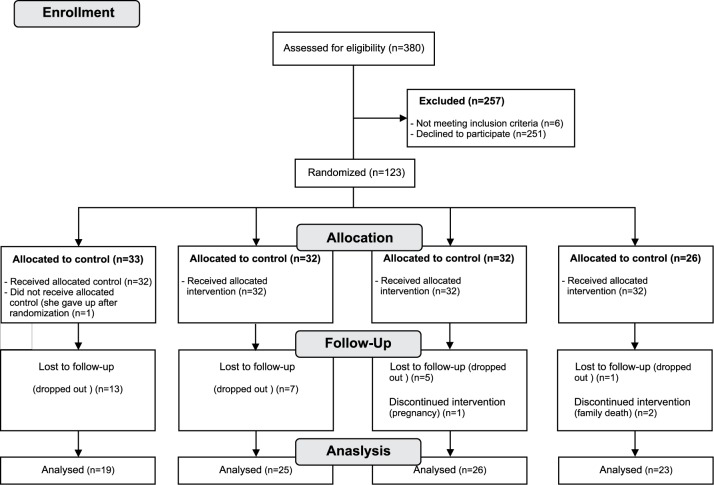
Participation flowchart.

Of the 380 recruited health professionals, only six did not meet the inclusion criteria and 251 did not have the time to participate. Therefore, we initially included 123 participants. Ninety-three participants had two complete evaluations and were therefore included in the analyses. The participation flowchart is shown in Fig 1.

### Procedures

At the first meeting with the participants, we ran a checklist to verify the inclusion and exclusion criteria and participants signed an informed consent form. Then, we randomized participants and conducted a pilot test with seven volunteers to evaluate any necessary adjustments to the study (1 control participant, and two participants for each of the intervention groups).

Participants were randomized using opaque, sealed and numbered envelopes and then randomly selected using a computer program with numbers generated by the Research Randomizer® in order to distribute participants among the four arms of the study:

- Group 1—control group (no intervention);
- Group 2—Monosensory intervention (mediated by touch) that involved daily body moisturizing with an odorless cream;
- Group 3—Bisensory intervention (mediated by touch and smell) that involved daily body moisturizing with a scented cream;
- Group 4—Multisensory intervention (mediated by touch, smell, hearing and vision) that involved daily body moisturizing with a scented cream whose fragrance was associated with the nature scenes shown in the accompanying audiovisual material.

The enrollment occurred continuously from July to October, 2014. The follow-up of each participant was always performed 30 days after the end of the intervention. The study was conducted at a single center. Data collection was completed in December 2014.

### Experimental intervention

The proposed intervention consisted of daily body moisturizing with the “Todo Dia Natura–Algodão” (cotton) cream, already commercially available (Groups 3 and 4) and a version of the same cream without fragrance for group 2. None of the packages contained references to the brand Natura® or any other cosmetic brand.

Each participant was asked to select a time of day to apply the moisturizer on the body (excluding the face), for a period of 30 days. In groups 2, 3 and 4, moisturizing consisted of light bodily self-massage for distribution and absorption of the moisturizer. Group 4 participants were also instructed to watch specially prepared audiovisual material on a tablet before and during the procedure. The audiovisual material consisted of a Power Point presentation with a sequence of images of nature and music that lasted two minutes and forty seconds. No functions were enabled on the tablet other than the playing of the selected images.

The image selection was based on studies of the impact of nature images on health [9–10]. We also used color, verbal, visual, tactile and olfactory stimuli associated with cotton to complement the picture sequence consisting of 17 photographs, mostly depicting flowers and landscapes. We used the literature to guide our audio selection: the traditional Irish song “Sweet Portaferry” [11], performed by Eve Watters on the CD Quiet Visit—Celtic Harp. This is a traditional Irish melody, with arrangement for harp, tonality in E-flat Major, slow tempo (35 beats/minute), compound compass (6/8), melancholic and sweet. This song, besides containing musical features with a relaxing effect [12–13], has a harp timbre, which is soft, warm and cozy like cotton [14].

### Assessments

The main outcome assessed in this study was stress. Secondary outcomes were self-esteem and subjective well-being (life satisfaction and positive and negative affect).

All ratings were carried out by the study team in the facilities of the Instituto de Ensino e Pesquisa do Hospital Israelita Albert Einstein (IIEP) (the teaching and research institute at Albert Einstein Hospital) and took place at four different times: at the beginning of the study (baseline), after 15 days, after 30 days (end of intervention), and at the 30-day follow-up. The control group underwent the same evaluations as the intervention groups. All participants from all groups were evaluated by a dermatologist at the beginning and end of the study.

At the baseline assessment, participants also completed a questionnaire regarding socioeconomic information: age, marital status, number of children, spiritual beliefs, education, work area, time since graduation, time of work experience in their field, work hours, income, presence of financial, physical or emotional difficulties, as well as practice and frequency of self-care activities. This information allowed us to characterize the participants and verify the homogeneity across randomized groups. The inventories completed by the participants are described below.

#### Stress inventory and cortisol collection

The ISS–Inventory of Symptoms of Stress (Lipp’s Inventory of Stress Symptoms for Adults) is a four-phase model adapted from the three-phase model developed by Selye (1936) and modified by Lipp [15]. It is composed by four dimensions (Q) that temporally divide symptoms into “the last 24 hours” (Q1–15 symptoms; the alert phase), “last week” (Q2 –up to 9 symptoms; the resistance phase) Q3 (more than 10 symptoms, the near-exhaustion phase), and “last month” (Q4–23 symptoms; exhaustion phase or Burnout). The symptoms are divided into physical and psychological dimensions, corresponding to the most frequent manifestations of stress. In ISS, a positive diagnosis of stress is based on the sum of the symptoms of each frame of the inventory (Q1 > 6; Q2 > 3 or Q3 > 9; Q4 > 8), allowing data to indicate not only that the person has stress, but also the phase where symptoms are predominant.

Salivary cortisol was collected at our institution’s clinical laboratory. Produced in the adrenal cortex, the steroid cortisol is involved in the stress response and is thus an objective measure of stress. The concentration of salivary cortisol has the same circadian rhythm as serum cortisol; therefore, the highest levels are measured around 8 am and the lowest during the night (between midnight and 4 am). The concentration of cortisol in saliva represents the free cortisol fraction and reflects the amount of cortisol in the blood. In this study, we used a noninvasive, painless and safe method that uses plastic tubes with cotton for the morning saliva collection [16]. There was only one collection per evaluation.

#### Self-esteem scale

The Rosenberg Self-Esteem Scale [17] is a one-dimensional measurement comprising 10 statements related to feelings of self-esteem and self-acceptance. The items are answered on a 4-point Likert scale including strongly agree, agree, disagree and strongly disagree. In this study we used the Portuguese adaptation of the scale [18], whose initial results indicated the one-dimensionality of the instrument and psychometric characteristics equivalent to those found by Rosenberg (1989). The level of self-esteem is rated as follows: High self-esteem—above 30; Average self-esteem—between 20 and 30; Low self-esteem—below 20.

#### Scales of subjective well-being

The Life Satisfaction Scale [19] consists of five items assessing one of the cognitive components of subjective well-being (e.g., in most ways, my life is close to my ideal; if I lived my life over, I would change almost nothing). Participants base their answers on a seven-point scale ranging from 1 (strongly disagree) to 7 (strongly agree). In this study, we used the translation and adaptation made for Brazil [20]. The results measured by this scale range from 5–35, and the higher the score, the higher the satisfaction (20 is considered neutral).

The Positive and Negative Affect Scale (Escala de Afeto Positivo e Negativo; EAPN) uses nine adjectives to access the valence of emotions [21]. The adjectives for positive affect were cheerful, happy, satisfied and fun, while those for negative affect were depressed, worried/anxious, frustrated, irritated/hostile and unhappy. The instrument aims to assess how much the participant experienced each of the emotions in the last few days, which is measured on a seven-point scale ranging from 1 = ‘Very Slightly or Not at all’ to 7 = ‘ Extremely. In this study, we used the Brazilian Portuguese adaptation [22–23] that includes the adjective "optimistic" for positive affect (in order to balance the number of items for both scale factors), which was verified and validated for use in the local population.

### Data analysis

Data analysis and the final report were performed from January to July, 2015.

Socioeconomic variables and the other variables of interest were described by absolute frequencies and percentages (for qualitative variables) or by summary measures (such as means and standard deviations, or medians and interquartile ranges), as well as minimum and maximum values in the case of numerical variables.

The distribution of numerical variables was evaluated using histograms and box plots and normality was analyzed by the Shapiro-Wilk normality test. The age of the participants was the only variable with normal distribution.

Comparisons of categorical variables between groups at baseline were performed by the Pearson’s chi-square or Fisher’s exact test. For numerical variables, we used one-factor ANOVA to compare age and the nonparametric Kruskal-Wallis test for the remaining variables. Comparisons ignored cases with no response.

To assess changes in the variables over time, we used generalized estimating equations [24] that consider the dependence between the measurements of the same individual at different times. We consider the first order auto-regressive (AR-1) covariance structure for the generalized estimating equations, which assumes a stronger correlation between measures at baseline and after 15 days than the correlation between measures at baseline and after 2 months, as an example. The models were adjusted separately for each group in order to evaluate the effect of time. For categorical variables, we used binomial and multinomial logistic models, and presented the results by estimated odds ratios with Evaluation 1 as the reference category, 95% confidence intervals for the estimates, and p values. For numeric variables, we used Poisson or Gamma distributions, with results presented by means ratios, with Evaluation 1 as the reference category, 95% confidence intervals for the estimates, and p values. The choice of distributions was done using the analysis of model residues.

All analyses were conducted using the package R (R Core Team, 2015) [25].

## Results

### Characterization of participants and self-care

Table 1 depicts a full description of individual and group characteristics. Ninety-three participants were analyzed who were mostly nurses (60%) between 21 and 58 years old. Groups did not differ in terms of age (p = 0.157). There were also no difference between groups for ‘time since degree’ and ‘time of work experience in the field’ (p = 0.722 and p = 0.410). The “time since degree” and “time of work experience in the field” has minimum and maximum values of one year and 30 years, respectively. Most participants were married in the control groups (58%), in the Monosensory group (64%) and in the Bisensory group (65%). In the Multisensory group, 39% were married. There were no significant differences between groups regarding marital status (p = 0.058) or education level (p = 0.192).

**Table 1:** Sample description by groups.

	Group				P
	Control	Monosensory	Bisensory	Multisensory	
**Age (years)–mean (SD)**	38.2 (10.2)	36.4 (8.2)	41.0 (9.2)	35.5 (8.9)	0.157
**Marital status**					
**Did not respond**	0 (0%)	1 (4%)	0 (0%)	0 (0%)	0.058
**Married/living with partner**	11 (58%)	16 (64%)	17 (65%)	9 (39%)	
**Separated/divorced**	3 (16%)	6 (24%)	2 (8%)	2 (9%)	
**Single**	5 (26%)	2 (8%)	7 (27%)	11 (48%)	
**Widow**	0 (0%)	0 (0%)	0 (0%)	1 (4%)	
**Has children**	9 (47%)	19 (76%)	21 (81%)	10 (43%)	**0.011**
**Number of children**					
**1**	2 (22%)	10 (53%)	9 (43%)	6 (60%)	
**2**	6 (67%)	7 (37%)	10 (48%)	3 (30%)	
**3**	1 (11%)	1 (5%)	2 (10%)	1 (10%)	
**4**	0 (0%)	1 (5%)	0 (0%)	0 (0%)	
**Education level**					0.192
**Technical**	1 (5%)	7 (29%)	9 (36%)	9 (41%)	
**Undergraduate**	13 (68%)	16 (67%)	12 (48%)	9 (41%)	
**Graduate**	5 (26%)	1 (4%)	4 (16%)	4 (18%)	
**Time since degree–median (IQR)**	8.0(2.0–18.0)	10.0(7.0–14.0)	10.0(7.0–18.0)	6.5(4.0–14.0)	0.722
**Time of experience working in the field–median (IQR)**	3.0(2.0–10.0)	7.0(3.0–10.0)	7.0(3.0–14.0)	6.0(3.0–13.0)	0.410
**Work area**					
**Did not respond**	0 (0%)	1 (4%)	0 (0%)	2 (9%)	0.348
**Open sector**	13 (68%)	21 (84%)	19 (73%)	18 (78%)	
**Closed sector**	6 (32%)	3 (12%)	7 (27%)	3 (13%)	
**Work hours**					
**Full time**	13 (68%)	16 (64%)	14 (54%)	13 (57%)	0.860
**Morning only**	3 (16%)	4 (16%)	6 (23%)	7 (30%)	
**Afternoon only**	3 (16%)	5 (20%)	6 (23%)	3 (13%)	
**Approximate monthly income**					
**1 to 3 sal.**	3 (16%)	4 (16%)	2 (8%)	7 (30%)	0.174
**4 to 10 sal.**	8 (42%)	16 (64%)	17 (68%)	14 (61%)	
**More than 10 sal.**	8 (42%)	5 (20%)	6 (24%)	2 (9%)	
**Undergoing financial difficulties**	4 (21%)	5 (20%)	10 (38%)	7 (30%)	0.435
**Undergoing physical or emotional difficulties**	6 (32%)	12 (48%)	12 (46%)	7 (30%)	0.472
**Frequently-performed self-care**					
**Healthy, balancedNutrition at regular hours**	10 (53%)	14 (56%)	13 (50%)	14 (61%)	0.888
**Regular physical activity**	10 (53%)	8 (32%)	6 (23%)	6 (26%)	0.171
**Eight hours of sleep/day**	5 (26%)	6 (24%)	10 (38%)	3 (13%)	0.247
**Acupuncture**	1 (5%)	1 (4%)	0 (0%)	2 (9%)	0.481
**Massage**	1 (5%)	3 (12%)	2 (8%)	2 (9%)	0.922
**Spiritual activity**	7 (37%)	11 (44%)	9 (35%)	12 (52%)	0.613
**Meditation**	1 (5%)	1 (4%)	1 (4%)	3 (13%)	0.621
**Leisure**	12 (63%)	15 (60%)	11 (42%)	12 (52%)	0.482
**Has a spiritual belief**	15 (79%)	24 (96%)	22 (85%)	20 (87%)	0.367
**Performs any otherself-care activities**	9 (47%)	11 (44%)	11 (42%)	11 (48%)	0.939
**Frequency of self-care**					
**More than four times a week**	2 (22%)	5 (45%)	2 (17%)	2 (17%)	0.175
**One to four times a week**	6 (67%)	4 (36%)	6 (50%)	10 (83%)	
**Less than once a week**	1 (11%)	2 (18%)	4 (33%)	0 (0%)	

Data represents the absolute number of participants in each category and the percentage within each group (inside parenthesis). Distinct cases are described in the table. IQR: 1^st^ quartile– 3^rd^ quartile. SD: Standard deviation. The only significant difference observed (p<0.05) was the higher number of participants with children in the Multisensorial group compared to the Unisensorial group.

Full-time work in the open sector was most prevalent, and around 50% of participants worked in the field for over seven years, with an approximate monthly income between 4–10 times the minimum wage. Overall, 28% of professionals were experiencing financial difficulties and 40% reported some physical or emotional difficulty.

Self-care was neglected by a significant portion of participants, considering that many reported not eating properly (45%), 68% did not practice regular physical activity and 74% did not often sleep eight hours a night. The most prevalent self-care practices were related to leisure (54%) and activities of a spiritual nature (42%), and 87% reported having some spiritual belief. Some complementary therapies practiced as self-care, such as meditation, massage and acupuncture, were not adopted by most health professionals. However, other forms of self-care were cited by 45% of participants, especially those related to beauty care focusing on skin, nails and hair.

The Fisher's exact test revealed that groups did not differ in terms of work area (p = 0.348), working hours (p = 0.860), income (p = 0.174), spiritual beliefs (p = 0.367), acupuncture practice (p = 0.481), massage (p = 0.922), meditation (p = 0.482) or amount of sleep (p = 0.247). The chi-square test revealed that groups did not differ in terms of financial difficulties (p = 0.435), physical/emotional difficulties (p = 0.472), nutrition (p = 0.888), physical activity (p = 0.171), spiritual activity (p = 0.613) or leisure (p = 0.939).

### Stress, self-esteem and well-being

Table 2 shows that at baseline, the groups were matched on all variables except cortisol levels: the Bisensory group had a higher proportion of participants with levels above the median when compared to the control group. In this analysis, we considered only the 79 participants who had at least two measurements during the course of the study so that the evolution of the participants could be evaluated. Because only 8 out of 79 participants presented cortisol levels outside the limits of normality (between 100 e 750nG/dL), we considered the categories formed by the median of the cortisol levels observed at baseline (217nG/dL).

**Table 2:** Comparison of variables across groups at baseline.

	Group				p-value
	Control(n = 19)	Monosensory(n = 25)	Bisensory(n = 26)	Multisensory(n = 23)	
**Cortisol -n (%)**					
** Below the median**	3 (21.4)	10 (47.6)	15 (68.2)	7 (31.8)	**0.023**^**a**^
** Above the median**	11 (78.6)	11 (52.4)	7 (31.8)	15 (68.2)	
**LIPP stress—n (%)**					
** No stress**	13 (68.4)	12 (48.0)	14 (53.8)	14 (60.9)	0.555
** Stress**	6 (31.6)	13 (52.0)	12 (46.2)	9 (39.1)	
**LIPP stress phase—n (%)**					
** No stress**	13 (68.4)	12 (48.0)	14 (53.8)	14 (60.9)	0.267
** Alert**	0 (0.0)	1 (4.0)	0 (0.0)	0 (0.0)	
** Resistance**	4 (21.1)	12 (48.0)	11 (42.3)	8 (34.8)	
** Near-exhaustion**	2 (10.5)	0 (0.0)	1 (3.8)	1 (4.3)	
**Self-esteem—n (%)**					
** Low**	12 (63.2)	11 (44.0)	11 (45.8)	13 (56.5)	0.544
** Average/High**	7 (36.8)	14 (56.0)	13 (54.2)	10 (43.5)	
**Negative affect–median (1^st^ quartile– 3^rd^ quartile)**	13.00[10.50; 17.50]	15.00[13.00; 21.00]	13.50[11.75; 15.50]	13.00[9.00; 17.50]	0.354
**Positive affect–median (1^st^ quartile– 3^rd^ quartile)**	24.00[22.50; 26.50]	24.00[20.00; 27.00]	23.50[18.75; 26.25]	24.00[19.00; 24.50]	0.470
**Life satisfaction–median (1^st^ quartile– 3^rd^ quartile)**	24.00[20.00; 30.00]	23.00[16.00; 29.00]	26.00[21.50; 29.25]	22.00[19.50; 27.50]	0.497

^a^Multiple comparisons showed differences between the Bisensory and control groups.

Data represents the absolute number of participants in each category and the percentage within each group (inside parenthesis).

Since 53 of 93 participants (57.0%) were stress-free at baseline according to LIPP, the comparison of the phases of stress over time was done descriptively and we observed that all groups (considering all participants) varied in stress scores to some degree, without changes in cortisol levels at the post-baseline assessments. Between evaluations 1 and 3, participants in the control group showed a 25% improvement and 17% worsening in stress; the Monosensory group showed 38% improvement and 14% worsening; the Bisensory group showed 38% improvement and 4% worsening, and the Multisensory group showed 9% improvement and 5% worsening.

The most prevalent physical and psychological symptoms are presented in Table 3, highlighting muscle tension presented by most participants in 24h, besides other anxiety-related symptoms. The feeling of constant physical tiredness prevailed during the previous week, with repercussions on memory, libido and emotional states. These symptoms, when evaluated in the previous month, revealed a profile of exhaustion accompanied by psychological distress.

**Table 3:** Main symptoms included in the LIPP Stress Inventory.

VISIT 1	Control		Monosensory		Bisensory		Multisensory	
**LAST 24H**	n	%	N	%	N	%	N	%
**Muscle tension (back, neck or shoulder pain)**	16	84.2	19	76	17	65.4	14	60.9
**Tightness in jaw/teeth grinding, biting of the nails or tip of pen**	7	36.8	7	28	9	34.6	5	21.7
**Dry mouth**	6	31.6	7	28	4	15.4	1	4.3
**Changes in appetite (eating a lot or having no appetite)**	6	31.6	12	48	13	50.0	5	21.7
**Sudden urge to start new projects**	6	31.6	6	24	5	19.2	8	34.8
**Insomnia, difficulty sleeping**	5	23.3	5	20	8	30.8	6	26.1
**Cold hands and/or feet**	2	10.5	6	24	7	26.9	9	39.1
**LAST WEEK**								
**Constant sense of physical tiredness**	11	57.9	13	52	1	38.5	8	34.8
**Constant tiredness**	8	42.1	1	40	11	42.3	7	30.4
**Memory problems, forgetfulness**	7	36.8	11	44	7	26.9	7	30.4
**Reduced libido**	7	36.8	6	24	11	42.3	11	47.8
**Excessive emotional sensitivity, gets emotional easily**	6	31.6	7	28	5	19.2	7	30.4
**Excessive irritability**	5	26.3	1	40	8	30.8	4	17.4
**LAST MONTH**								
**Exhaustion**	7	36.8	9	36	12	46.2	7	30.4
**Daily distress or anxiety**	3	15.80	1	44	8	30.8	4	17.4
**Constantly thinking about the same subject**	5	26.30	9	36	8	30.8	2	8.7
**Irritability for no apparent reason**	5	26.30	6	24	5	19.2	7	30.4

Data represents the absolute number of participants in each category and the percentage within each group.

In subsequent evaluations, we did not observe changes in the pattern of symptoms, but rather a lower prevalence in the resistance and near-exhaustion phases among the participants of the Monosensory and Bisensory groups, in which we observed a reduction of stress over time in Table 4.

**Table 4:** Study outcomes regarding stress (LIPP and cortisol) and self-esteem (Rosenberg Scale) for all groups over time.

	Group	Comparisons to baseline					
		Evaluation 2		Evaluation 3		Follow-up	
		Ratio (95% CI)	p	Ratio (95% CI)	p	Ratio (95% CI)	p
LIPP	Control	1.19 (0.41–3.48)	0.749	1.41 (0.39–5.15)	0.604	0.74 (0.1–5.23)	0.761
	Monosensory	0.46 (0.22–0.97)	**0.041**	0.25 (0.07–0.85)	**0.026**	0.83 (0.25–2.73)	0.758
	Bisensory	0.20 (0.05–0.75)	**0.017**	0.22 (0.07–0.72)	**0.012**	0.24 (0.06–0.91)	**0.036**
	Multisensory	0.95 (0.46–1.97)	0.899	0.75 (0.35–1.61)	0.463	0.85 (0.27–2.63)	0.774
Cortisol	Control	1.57 (1.12–2.20)	**0.009**	0.88 (0.60–1.30)	0.529	0.84 (0.72–0.98)	**0.028**
	Monosensory	1.12 (0.85–1.46)	0.428	0.97 (0.58–1.64)	0.918	1.78 (1.23–2.59)	**0.003**
	Bisensory	1.07 (0.78–1.46)	0.684	1.09 (0.84–1.41)	0.511	1.18 (0.92–1.51)	0.205
	Multisensory	0.87(0.56–1.37)	0.556	0.79 (0.63–0.99)	**0.036**	0.99 (0.77–1.26)	0.928
Self-esteem	Control	1.14 (0.6–2.15)	0.697	0.43 (0.17–1.08)	0.071	0.96 (0.5–1.84)	0.892
	Monosensory	1.73 (0.69–4.32)	0.243	0.60 (0.18–1.93)	0.390	0.49 (0.12–2.05)	0.332
	Bisensory	3.28 (1.05–10.26)	**0.041**	0.57 (0.16–2.12)	0.406	1.86 (0.5–6.89)	0.355
	Multisensory	1.22 (0.52–2.86)	0.656	0.28 (0.11–0.75)	**0.012**	3.89 (0.85–17.8)	0.080

For LIPP, we show the odds ratio of stress at each evaluation compared to baseline. For self-esteem, we show the odds ratio of low self-esteem at each evaluation compared to baseline. For cortisol, we show the mean ratio of cortisol levels at each evaluation compared to baseline.

For this inferential analysis, we only considered participants who were in the resistance or near-exhaustion phase, thus excluding participants who did not have stress. All groups showed some variation in stress stages throughout the study, but there were no significant changes at the second evaluation (15 days). According to Table 4, the Monosensory and Bisensory groups showed a significant reduction in stress on the third assessment compared to the initial assessment, but only the Bisensory group maintained this stress reduction at follow-up.

Regarding salivary cortisol levels, the control group had higher levels on the second evaluation, even though it improved by 16% at follow-up (p = 0.028) compared to baseline. The Monosensory group was also worse at follow-up (p = 0.003). Only the Multisensory group showed a significant reduction on the third evaluation (21%, p = 0.036) and also showed a significant improvement in self-esteem (72%, p = 0.012), which was not maintained at follow-up.

Regarding the Rosenberg Self-Esteem Scale, we evaluated the average and high categories together due to the small number of participants classified as high self-esteem. We used logistic regression models to estimate the odds ratios of presenting low self-esteem against presenting average or high self-esteem at each of the times and separately, by groups (Table 4). The groups showing significant changes were the Bisensory group, with a mean ratio between 2^nd^ and 1^st^ evaluation estimated in 3.28 (95% CI: 1.05 to 10.26 and p = 0.041), and Multisensory group, whose score on evaluation 3 was estimated at 0.28 times the mean score of evaluation 1 (95% CI: 0.11 to 0.75, p = 0.012)

Regarding life satisfaction, the only group showing significant changes was the Bisensory group, with an 8% improvement, on average, from the first evaluation to the time of follow-up (95% CI: 2% to 15% and p = 0.016).

Changes in positive affect were evaluated using a generalized linear mixed model with a gamma distribution and the only significant change was observed in the Bisensory group, whose score on evaluation 3 was 10% more than on evaluation 1 (95% CI: 2% to 19%, p = 0.011) and negative affect was 12% less than on evaluation 1 (95% CI: 3% to 21% lower, p = 0.014).

## Discussion

In order to assess the impact of a self-care intervention mediated by the senses on the stress, self-esteem and well-being of health professionals working in a hospital environment, we needed to identify the basic self-care profile among the participants tested. The first thing that caught our attention was the fact that self-care was overlooked by many participants, with basic human needs not being fully met by a significant number of participants. Maslow’s hierarchy of basic human needs [26] indicates that physiological, safety, love and/or social, esteem and self-realization needs, when not met, can generate dissatisfaction and preclude meeting human needs for growth, self-realization and self-development.

Study participants showed deficiencies mainly in keeping adequate levels of sleep, nutrition, physical and leisure activities, and half of them also reported low self-esteem. The sum of these factors can increase the risk not only of stress, but also of becoming ill over time, either physically or psychologically. Other authors have reported a failure by health professionals to meet basic human needs, particularly nursing staff [27], which suggests how this situation contributes to a context of dissatisfaction, which leads to lesser care, not only of the self, but also of others, thus compromising the workforce and quality of care.

In health institutions, professionals care for patients, worry about their rehabilitation, strive to prevent their patients’ health problems and to ensure safety, but paradoxically present a disregard and neglect toward their own health [28]. The neglect of self-care seems to stem from the lack of time to eat properly, take care of oneself physically and aesthetically, and also spend less time on oneself due to work [29]. We were surprised by the lack of time and availability of participants to take part in the study. Although a large number of participants was interested in participating during the recruitment stage, they showed poor compliance and did not attend subsequent evaluations, especially those scheduled for 15 days and 30 days following the study (follow-up).

It is common to see that caretakers do not allow themselves the time and conditions to be cared for, nor do they receive support or the proper conditions for self-care. This creates an almost generalized situation of alienation that is perceived as being absolutely "natural" and part of their professional choice, which can be traced back to historical descriptions of self-denial [30]. The increasingly demanding and selective job market, with no signs of improving work conditions or payments, also leads the professional to increase his/her dedication and commitment, which reduces time for the self and one’s personal life [31]. Therefore, these professionals tend to be constantly dominated by a sense of ambivalence, as a result of failing to perform, mainly due to the huge amount of work that is required of them in a short amount of time. When they realize this, professionals experience feelings of irritation and frustration, leading them to feel constrained by contextual factors and far from their personal and professional ideals [32].

Other forms of self-care that help reduce stress and promote well-being, such as meditation, massage, and acupuncture, were also not mentioned often by participants. By contrast, aesthetic forms of care (skin, nails and hair) were mentioned, reflecting concern with one’s image/appearance, which is fairly common among women. However, the daily self-massage during the application of moisturizer constituted not only a moment of care, but also an opportunity to look at oneself, at one’s body, bringing to consciousness a possible discrepancy between the idealized body (dictated by the media and fashion) with the actual body that may have been neglected in several respects. Perhaps this was one of the reasons why participants in the Bisensory group had worse self-esteem at the second evaluation, as they had to look at their own bodies, which may not have received adequate attention for some time. In a study conducted in another hospital on the perception of self-care of nursing professionals, concern with aesthetics was also present. Furthermore, a self-assessment exercise revealed participants’ discomfort with their own body, which they regarded as being outside the desired aesthetic standards [33]. Engaging in self-care practices is a challenge, and studies that investigate ways of teaching health professionals to apply these practices should be conducted [34].

Stress, when studied in the work environment, pervades many factors related not only to organizations, but also to the lifestyle adopted by professionals and even individual characteristics, which makes their analysis and interpretation rather complex. When analyzing the sociodemographic profile of the participants studied, we observed some factors that may be related to stress, such as the fact that most women were married with children (and some were divorced), which alone represents a double load of work and responsibilities. Other factors also mentioned by many participants included coping with financial, physical or emotional difficulties.

The most represented professional category in this study was nursing, showing a particular interest among these professionals in participating in studies on this topic. This is why our discussion is based mostly on studies conducted with these professionals. Although it affects all categories of health professionals, stress in nursing has been studied the most, as nurses make up a significant portion of professionals working in hospitals. The literature reports levels of stress among these professionals that vary according to type of institution and work units, as well as personal coping methods.

Analyzing the stress levels of intensive care nurses revealed the already recognized hegemonic predominance of women (91.6%) working in the healthcare sector, where 36.5% presented medium stress levels and 23.6% were at high risk for elevated stress [35]. The prevalence observed by those authors is higher than that observed in the present study, which may be related to organizational and labor sector characteristics. In our study, most professionals worked in open inpatient units at a private hospital, which differs from studies where most professionals worked in intensive care units in public hospitals. Another study also reported a higher prevalence of psychological symptoms among nurses working double shifts relative to those working single shifts [36]. Other authors also observed this discrepancy in stress levels across institutions. Similar to our findings, in a study conducted in a university hospital, the authors observed low levels of stress related to the characteristics of the study population, which were influenced by the choice of the work unit, conducting graduate studies, single employment and adoption of strong coping strategies [37].

Nursing seems to generate high levels of stress due to: inflexible hierarchical structure, with inflexible rotations, shifts, rules and regulations; task and responsibility overload, with high demand levels, interpersonal problems at work; exposure to loss, suffering and death; an imbalance of sleep, bodily rhythms, eating, and family and social life; an imbalance between satisfaction and emotional gain with unpleasant situations and personal and professional stress; besides the demand overload from various sources and with different levels of complexity (own, institutional, other professionals, the patient, the family and society) [31].

When psychological pain resulting from stress is detected, the literature alerts to the need to restore the state of physical and psychological health of nurses and other professionals who work mainly in care activities [38]. It is known that the emotional profile of nurses undergoes changes during a single shift, which can be attributed to the wear and stress associated with the act of providing assistance, especially in units where there is a demand for high-level skills and immediate responses, resulting in fatigue at the end of the shift [39].

The first reaction to work-related stress is the feeling of physical and mental overload and relationship difficulties. Often the high intensity of the imbalance leads to exhaustion, also known as Burnout Syndrome, an excessive work-related stress response that is presented in three dimensions: emotional exhaustion, depersonalization, and reduced efficiency [40–41]. Another study conducted with nurses in the same institution showed that 55.4% had a propensity for burnout syndrome, as well as health problems related to work and emotional complaints [42].

Fragmenting, reductionist, biasing and alienating situations lead to defensive behaviors, especially denial, isolation and sublimation, which are manifested as avoidance and reinforced by the heroic persona trying to rescue the other from suffering, sparing no effort, thus sacrificing his/her own health and renouncing life outside of this role. Depersonalization via self-denial (reflected in neglected self-care) hiding distress when faced with tense situations is transformed into heroic impulses aligned with the capitalist production mode, that relegate workers to self-neglect, causing extensive damage to family and social life [31].

The physical and psychological symptoms presented by participants in this study did not differ from those reported in the literature, especially muscle tension and constant physical wear that culminates in exhaustion. Some scholars associate these symptoms with chronic fatigue syndrome, defined as a wearing of physical or mental energy that can be recovered through rest, nutrition or specific clinical guidance. Fatigue may have repercussions on various systems of the body, causing multiple changes in functions that lead to reduced performance at work, absenteeism and a number of psychological, family and social disturbances [43]. Based on the data, we can say that participants are at risk of not only developing chronic fatigue syndrome, but also of reaching near-exhaustion and exhaustion over time, considering that they also reported sleep disorders, an inadequate number of rest hours, as well as irregular eating, all aspects that are essential to the recovery of fatigue. It is noteworthy that a significant number of participants was in the resistance phase at the beginning of the study.

The Bisensory group, who used the cream with fragrance, showed better results than the Monosensory group (who used the cream without fragrance) and the multisensory group (who also had the association with the senses of hearing and vision through the video). This indicates that smell associated with touch was more effective than touch alone, highlighting the importance of scent in the proposed method of self-care.

The application of massage (or self-massage) has several therapeutic effects by acting on the individual’s psychological and physiological functions. It relieves pain and muscle tension, promotes relaxation [44], attention and affection, and increases the feeling of well-being [45]. Tactile stimulation affects skin functions, which can be grouped into functions of protection, information and deep regulation, which are directly controlled by the central nervous system [46]. The skin is a sensory apparatus, home to the sense of touch, which contains an enormous area of exposed nerve endings. It is known that massage provides relaxation and influences emotional states [47]. Inner feelings such as tension, stress and anxiety are usually replaced by calmness and tranquility, and salivary cortisol is reduced [48]. The act of touching every day also induced a reduction in negative affect and an increase in positive affect in the Bisensory group only when associated with olfactory stimulus.

The olfactory stimulus seems to have contributed synergistically to the tactile stimulus, leading to better results in the Bisensory group relative to the Monosensory group that received only tactile stimuli. Because the fragrance used is protected by a trade secret, we will not discuss its specific properties, but its soft scent seems to promote similar results to those observed for some essential oils.

Fragrances have often been used to reduce stress, including work-related stress [49]. Although they used different fragrances for different durations, our findings are similar to those of a study conducted with healthy individuals who participated in a massage program that used essential geranium and lavender oils once a week for four weeks. In that study, participants experienced a reduction in anxiety and increased feelings of well-being. The researchers also found significant changes in several neurobiological markers, including salivary cortisol. The salivary cortisol collected pre- and post-intervention decreased from 4.7 ng/dl to 4.3 ng/dl; however, the basal salivary cortisol did not change after 30 days, showing that the intervention had only short-term effectiveness [50].

Our data also did not indicate significant changes in cortisol levels, since all oscillations occurred within the normal range. This suggests that the adoption of Cortisol levels as a biomarker in studies about stress should be done with caution. Two aspects should be considered: 1) cortisol may be a more appropriate biomarker for studies in which levels above the normal range are an inclusion criterion; 2) the evaluation of variations within normality parameters could help identify a way of acting preventively; in other words, the effects, even if seen several times during a short period, may contribute so that cortisol levels do not rise to critical levels. We believe this is an interesting perspective that should be considered in future investigations.

The literature has shown that nature scenes can positively affect emotional states, decrease stress levels and pain, and promote well-being [51]. Although evidence exists for the benefits of nature on health and welfare [52] even when presented virtually [53], we observed that there was no reduction in stress for the Multisensory group (who used the scented cream together with the video with pictures and music); in fact, they showed even lower improvement than the control group. Two considerations may explain this result: 1) the sensory hyperstimulation may have resulted in a sensory overload, which instead of producing feelings of well-being may have been construed as an additional task; 2) although we did not measure it formally, many participants spontaneously voiced that they had concerns regarding the tablet (fear of theft, damage), which may have made it one more source of tension and stress, making the material provided on the tablet secondary. On the other hand, the Multisensory group was the only one that showed improved self-esteem and reduced cortisol levels at the third assessment.

Self-esteem is considered a major mental health indicator. It influences how the health professional will perform within his/her practice. Self-esteem has been defined as the perception that the individual has of him/herself and within the professional realm; it reflects the individual’s internal and external beliefs when he/she compares him/herself to other professionals [54]. People with low self-esteem tend not to be as highly motivated as those with high self-esteem and therefore tend to judge everyday situations negatively, are less assertive, and display a decline in productivity, greater susceptibility to stress, less job satisfaction, lower commitment and lower levels of enthusiasm [55]. Self-esteem is vital to nurses, for they make decisions that affect the care of patients. High self-esteem is related to personal well-being and to the delivery of healthcare that is affordable, competent and safe. It is also a protective factor against burnout [56].

In this study, participants had low self-esteem, even though they worked in a private institution considered a reference in Latin America, which could be considered a source of pride and professional recognition leading to a positive self-evaluation. However, this is a delicate subject that has not been well studied.

As for subjective well-being, with the exception of the Bisensory group, we did not observe an improvement in participants’ satisfaction with life. We know, however, that life satisfaction may be influenced by a number of other factors [57], including those of a financial nature, as pointed out by one-third of participants.

The life satisfaction assessment conducted with doctors, nurses and psychologists revealed that an important factor for these three professional categories is income. The correlation between income and satisfaction has been specifically observed in developing countries such as Brazil. Women display even greater dissatisfaction and express the view that they "should earn more". There is a recognized income gap between genders (and between categories) that characterizes a degree of discrimination suffered by women in the labor market, both in Brazil and in the United States [58]. On the other hand, the Bisensory group, which showed a significant reduction in stress, also showed decreased negative affect and increased positive affect, with a better perception of life satisfaction at follow-up.

Defining well-being is difficult, as it may be influenced by variables such as age, gender, socioeconomic status and culture. A broad definition states, a person with a high sense of well-being is satisfied with life, has frequent positive affect, and relatively little negative affect [59]. Pleasurable events when experienced daily (such as self-care in the Bisensory group) are related to positive affect, while unpleasurable events, are associated with negative affect. Satisfaction and happiness result from the accumulation of these specific moments, and these happy experiences [60].

Welfare levels are relevant indicators in mental health, considering that low welfare can increase depressive symptoms. The assessment of well-being has an important practical use to evaluate and prevent depression [61]. Therefore, corporate health services in hospitals should consider implementing these assessments.

Researchers have pointed out is the importance for health institutions to develop incentives to encourage health professionals to adopt healthier lifestyles that may protect against stress caused by the demands of work in hospitals [62–63]. Conducting this research on the self-care of health professionals in the hospital provided participants with an opportunity for reflection and showed that a simple measure, such as daily body moisturizing mediated by touch and smell can reduce stress-related symptoms.

### Study limitations

One limitation of the study was the reduced sample size composed of health professionals from a single private health institution. Low adherence of participants to the four evaluations may also have affected the analysis of the interventions.

The fact that most participants worked in nursing may also be considered a limitation since other professions have different work demands that can influence stress levels, self-esteem and well-being and may not have been represented in the sample studied. On the other hand, because nursing represents the largest professional category in the hospital environment, the present analysis serves as a model for the development of other studies on this topic with other professional groups and other health institutions.

## Conclusions

In this study, we observed that the self-care method (mediated by touch, smell, sight and hearing) does not reduce stress. However, it improved participants’ self-esteem. This finding indicates that self-esteem and stress are not always related; opposing what has been suggested by several studies. Self-care intervention mediated by touch and smell can help in fighting the stress in health professionals and thus should be further explored as a strategy to ameliorate the stress that these professionals usually carry in their professional lives.

## Supporting information

S1 Text Original protocol. (PDF)

S2 Text CONSORT CHECKLIST. (PDF)

S3 Text ClinicalTrial.gov–Protocol registration. (PDF)

S4 Text Letter of ethical approval. (PDF)

S5 Text Translated protocol (main points). (PDF)
