# Supplementary material for: Stress, self-esteem and well-being among female health professionals: A randomized clinical trial on the impact of a self-care intervention mediated by the senses
Source: PLoS One. 2017 Feb 27;12(2):e0172455. doi: 10.1371/journal.pone.0172455 (PMC5328264; doi:10.1371/journal.pone.0172455)
Supplement: S1 Text — (PDF) [file pone.0172455.s001.pdf]

Versão 2 – 27FEV2014

PROJETO H-SENSES: Autocuidado mediado pelos sentidos: avaliação do impacto na autoestima, stress e bem-estar de mulheres profissionais de saúde.

Núcleo de Pesquisa Assistencial–Instituto Israelita de Ensino e Pesquisa Albert Einstein

Hospital Israelita Albert Einstein (HIAE)

Investigador principal: Eliseth Ribeiro Leão

1. Objetivo do projeto e problemas abordados

Este estudo tem como objetivos: explorar as relações entre bem-estar, autoestima e stress de mulheres profissionais de saúde; comparar três intervenções de autocuidado mediada pelos sentidos (monossensorial - tato, bissensorial tato e olfato e multissensorial – tato, olfato, visão e audição) e conhecer a percepção das mesmas sobre essa experiência.

Partimos da premissa de que, na assistência à saúde, um cuidado com qualidade é fruto da competência profissional, aliada à condição de profissionais saudáveis e em equilíbrio. O autocuidado é fundamental para quem pretende cuidar do outro, mas temos observado, tanto na prática clínica quanto docente, que a consciência disto e a adoção de práticas saudáveis por profissionais já formados ou em formação constituem um desafio.

O ambiente de trabalho dos profissionais de saúde comumente é considerado estressante, tendo em vista que necessitam lidar com situações delicadas, que incluem sofrimento, risco de morte, tomadas de decisão que precisam ser rápidas e acuradas, além de conflitos gerados nas relações interpessoais. Há um discurso corrente de que necessitamos cuidar de quem cuida, entretanto, poucas são as intervenções estudadas e validadas para redução efetiva do stress desses profissionais que repercutam em estados de bem-estar. Há pouco espaço para a discussão deste tema durante a jornada de trabalho, bem como para se aprender técnicas e medidas efetivas de gerenciamento de stress.

O autocuidado constitui ponto central da Teoria de Dorothea Orem, que é ensinada no curso de graduação em enfermagem e adotada em muitas instituições hospitalares na Sistematização da Assistência de Enfermagem, para nortear a orientação de autocuidado aos pacientes. Entretanto, temos observado que há uma lacuna entre o que se aprende e o que se pratica, muitas vezes, na área da saúde. Observamos que muitos profissionais não cuidam de si mesmos, adotam hábitos pouco saudáveis de alimentação, lazer e outros, o que pode prejudicar a confiança e a adesão dos pacientes às orientações de saúde quando fornecidas, principalmente aquelas que objetivam o autocuidado.

Para se entender a teoria do autocuidado é necessário definir os conceitos relacionados, como os de autocuidado, ação de autocuidado, e demanda terapêutica de autocuidado. Autocuidado é a atividade que os indivíduos praticam em seu benefício para manter a vida, a saúde e o bem estar. Ação de autocuidado é a capacidade de o homem engajar-se no autocuidado. E demanda terapêutica de autocuidado são os fatores que devem ser trabalhados, controlados e modificados no individuo por afetarem o funcionamento do seu organismo e o seu desenvolvimento humano (Orem, 1995).

O autocuidado precisa ser aprendido e, sobretudo, vivenciado. Por outro lado, pouco se sabe sobre quais intervenções são eficazes e comprovadas cientificamente, quando se trata do manejo do stress, e de medidas que promovam a autoestima e o bem-estar de mulheres profissionais de saúde.

Há algumas décadas o stress dos enfermeiros, bem como de outros profissionais da área da saúde, vem sendo estudado. Os níveis de stress são às vezes tão elevados que levam os profissionais a desistirem da profissão que abraçaram, a conhecida *síndrome de burnout*. Estudo sobre esta síndrome entre enfermeiros constatou altos níveis de exaustão emocional, despersonalização, além de baixo nível de realização profissional. Mostrou ainda as seguintes associações: alto nível de exaustão emocional e realizar frequentemente/sempre tarefas com muita rapidez (p=0,039) e receber salário incompatível com o esforço empregado (p=0,016). Para 19,0% dos participantes do estudo, pelo menos duas das três dimensões do instrumento apontavam alta propensão à síndrome. (Galindo et al; 2012).

Devido a elevadas demandas de trabalho, entre outros aspectos, níveis de stress são observados entre os diversos profissionais de saúde e resultam em sintomas físicos e psicológicos, tais como: nervosismo, pesadelos, irritabilidade, cefaléia, insônia e problemas gastrointestinais (Tsai, Liu, 2012). O desequilíbrio causado pelo stress muitas vezes repercute também em baixa autoestima e diminuição do bem-estar subjetivo.

A ênfase do cuidado em saúde recai predominantemente sobre a assistência que é prestada aos pacientes, ficando o autocuidado do profissional relegado a um segundo plano.

A negligência do cuidado de si parece decorrer da falta de tempo para alimentar-se adequadamente, cuidar-se física e esteticamente e, ainda, do abdicar do uso do tempo para si em prol do trabalho. Parcela significativa dos profissionais da saúde é constituída por mulheres, que muitas vezes têm dupla ou tripla jornada de trabalho, o que torna essa situação ainda mais crítica.

O imaginário social conduz à impressão de que a profissional da área da saúde, o ser humano-educadora-cuidadora, não adoece e não se cansa. Assim sendo, frequentemente aquela que presta cuidados ao outro acaba por esquecer-se de si mesma. Estudiosos sobre o tema apontam a necessidade de resgatar o cuidado do seu próprio eu para o exercício pleno da profissão e da realização como seres humanos (Baggio, Formaggio; 2007).

É imperioso, portanto, investigar formas de autocuidado que sejam passíveis de serem aprendidas e incorporadas no cotidiano das profissionais de saúde, formas essas que se forem comprovadas como efetivas, tenham condições de ser incorporadas no cotidiano como uma forma saudável de melhor cuidar de si, para um cuidar melhor do outro.

Neste sentido, este projeto busca estar alinhado ao conceito de bem-estar/estar bem da Natura, empresa parceira neste projeto, ao considerar que o objeto a ser investigado busca avaliar o quanto é possível promover uma “relação harmoniosa, agradável do indivíduo consigo mesmo, com seu corpo e uma relação empática, bem sucedida, prazerosa do indivíduo com o outro, com a natureza da qual faz parte, com o todo”.

Percebemos o mundo pelos nossos sentidos do tato, audição, visão, olfato e paladar. Por isso, neste estudo, a proposta de uma intervenção de hidratação corporal será avaliada em sua relação com os sentidos, de forma monossensorial (tato), bissensorial (olfato e tato) e multissensorial (olfato, tato, audição e visão), a fim de verificarmos o impacto sobre os níveis de stress, bem-estar subjetivo (compreendido como satisfação com a vida do individuo) e autoestima (satisfação consigo) das profissionais de saúde.

As hipóteses deste estudo são:

1) Existe uma correlação inversa entre bem-estar e autoestima e níveis de stress.
2) O grupo da intervenção monossensorial apresentará maior redução do stress e melhora da autoestima e bem-estar do que o grupo controle.
2) O grupo da intervenção bissensorial apresentará maior redução do stress e melhora da autoestima e bem-estar do que o grupo controle e o grupo de intervenção monossensorial.
3) O grupo da intervenção multissensorial apresentará maior redução do stress e melhora da autoestima e bem-estar do que o grupo controle e os grupo de intervenção monossensorial e bissensorial.
4) O grupo da intervenção multissensorial apresentará maior adesão ao autocuidado do que o grupo controle, grupo de intervenção monossensorial e bissensorial.

2. Estado da Arte

O stress dos profissionais da saúde tem sido objeto de investigação científica devido aos custos e efeitos ao nível individual e organizacional. Há mais de duas décadas os resultados de estudos internacionais vêm demonstrando os potenciais efeitos negativos do stress ocupacional na saúde e no bem-estar dos profissionais de saúde, nomeadamente, em termos da baixa satisfação (Blegen, 1993), das queixas físicas e psicológicas (Cooper, Dewe, O’Driscoll, 2001) e do absenteismo (Borda & Norman, 1997; Cascio, 1995). As mulheres, em particular, apresentam maior tendência para os problemas relacionados ao stress ocupacional (Silva, Gomes, 2009).

A primeira reação do stress ligado ao trabalho é a sensação de exaustão, esgotamento, sobrecarga física e mental e dificuldades de relacionamento. Muitas vezes a intensidade do desequilíbrio é tão elevada que levam ao esgotamento, conhecida também como Síndrome de Burnout, uma reação de stress excessivo relacionada ao trabalho que se apresenta em três dimensões: exaustão emocional, despersonalização e ineficácia. Elevadas prevalências desta síndrome têm sido observadas entre médicos (Barros et al, 2008).

O stress na enfermagem, que corresponde a significativa parcela dos profissionais que atuam nos hospitais, também tem sido amplamente estudado. Levantamento dos níveis de stress dos enfermeiros em Unidades de Terapia Intensiva das regiões do Brasil revelou o já reconhecido predomínio hegemônico de mulheres (91,6%) que atuam na área assistencial, em que 39,9% apresentaram baixo nível de stress, 36,5% nível médio de stress e 23,6% caracterizaram alerta para alto nível de stress. A análise geral das regiões do Brasil demonstrou que níveis mais elevados de stress são característicos da região sudeste (Guerrer, Bianchi, 2011).

Outros autores alertam para a necessidade de restaurar o estado de saúde físico e psicológico dos enfermeiros que trabalham principalmente em atividades assistenciais, também em unidades abertas (Negeliski, Mautert, 2011). A predominância de sintomas psicológicos tem sido observada, sendo mais elevada entre enfermeiros que tem dupla jornada de trabalho em relação aos com jornada única (Páfaro, Martino, 2004). O perfil emocional dos enfermeiros sofre alterações no decorrer do plantão, o que pode ser creditado ao desgaste e ao stress próprios da atividade de prestar assistência, sobretudo nas unidades, onde há exigência de alto nível de habilidades e necessidade de respostas imediatas em emergências que resultam em cansaço relevante ao final do plantão (Martino, Misko, 2004). Isso reforça a necessidade de autocuidado dos profissionais para que suas energias sejam recarregadas e assim possam voltar a experimentar níveis de bem-estar que contribuam para sua saúde e qualidade de vida, além de contribuir para um melhor desempenho profissional.

Stress, bem-estar e autoestima são estados intimamente ligados, uma vez que o desequilíbrio físico e emocional afeta a percepção de bem-estar, sendo demonstrada que a autoestima também está relacionada de forma significativa com o bem-estar (Freire, Tavares; 2010).

Embora níveis de stress sejam evidentes entre os profissionais de saúde, a literatura é escassa em estudos de intervenção que indiquem medidas efetivas de autocuidado para o manejo de stress. Investigadores têm questionado ainda sobre como estimular a percepção dos profissionais da equipe enquanto cuidadores, para a promoção do cuidado de si (Vieira, Camada, 2006)

As experiências sensoriais têm sido exploradas na literatura em situações clínicas diversas, que indicam o potencial de produção de bem-estar, e que podem ser úteis em relação ao manejo do stress.

O estímulo tátil, quando utilizado com técnicas de massagem, resulta em melhora nos padrões de vigília (constatado pelo uso do Eletroencelalograma) e tônus vagal elevado, levando os indivíduos a um estado mais relaxado, com predomínio da atividade parassimpática (Field, Ironson, Scafidi, et al; 1996), diminuição da pressão arterial em pacientes portadores de diabetes (Castro-Sanchez et al; 2010), alívio da dor (Moyer, Rounds, Hannum, 2004), adaptações fisiológicas ao stress (Moraska et al; 2010); aumento da circulação local, aumento da flexibilidade muscular e intensificação da movimentação da linfa (Imamura, Furlan, Dryden, Irvin; 2008).

Os aromas têm sido investigados em relação aos efeitos psicológicos que produzem. Essa abordagem se baseia principalmente na memória olfativa. Os aromas têm acesso direto ao sistema nervoso, entram sem grandes “filtros”. Dessa forma, são rapidamente armazenados juntamente com a memória da situação que o acompanhava, razão pela qual normalmente pode evocar a situação com a qual estava associado inicialmente (Pert, 2003), o que é de particular interesse na ancoragem de sensações de bem-estar.

Inúmeros estudos têm apontado a utilização de óleos essenciais aplicados em inalação, sem, contudo, uma explicação clara de como os efeitos são desencadeados. Sabe-se que os aromas influenciam no funcionamento dos sistemas imune e endócrino a partir de sua atuação no processo de stress, via alteração de humor, comportamento, emoção e também via alterações químicas (Davidson; 2002).

No âmbito da visão, os estudos tem demonstrado que pacientes que tem a vista de suas janelas voltadas para jardins tem melhor recuperação em relação aqueles que são privados de uma bela paisagem (Ulrich, 1984). O contato com a natureza sempre esteve associado a sensações de equilíbrio e bem-estar. Os chamados jardins de cura têm sido explorados ao longo dos tempos (Marcus, Barnes; 1995) e, na atualidade, constituem aspecto importante na arquitetura das instituições hospitalares, como um fator de influencia na promoção e recuperação da saúde. Porém, nem sempre e possível criar um ambiente natural e programas artificiais são criados para minimizar a ausência deste recurso e seus consequentes benefícios.

Há muita diferença entre ver uma paisagem produzida artificialmente de uma paisagem natural. Assim como há diferença, por exemplo, entre ouvir um CD ou um concerto ao vivo. Há um campo de energia, vibrações, odores e relações que não existe na primeira situação. Contudo, há uma tendência humana a se render ao belo e podemos vivenciar uma experiência estética, que produza emoções e altere nossos neurotransmissores, quando ficamos expostos a uma bela fotografia, uma obra de arte ou uma bela musica.

Nessa linha de raciocínio é que propomos a visualização de cenas da natureza, na perspectiva de que se não é possível ir até a natureza, é possível trazer um pouco da natureza ao cotidiano das profissionais da saúde, favorecendo um estado de presença. A presença pode ser definida como a subjetiva experiência de estar em um lugar ou ambiente, mesmo quando se está situado fisicamente em outro (Witmer, Cantor, 1998).

O contato virtual é um meio bastante familiar na atualidade, quer seja via computadores, *tablets* ou celulares, e necessita também ser considerado quando propomos alguma intervenção. O conceito de "*persuasive technology*" tem permeado programas educativos *web-based* e é definido como a tecnologia designada para alterar comportamentos e atitudes por meio da persuasão e influência social, mas não pela coerção. Essas tecnologias são usadas regularmente em vendas, diplomacia, política, treinamento militar, saúde pública e em gestão e podem ser usadas em qualquer área de interação humano-humano ou humano-computador (Fogg, 2002).

E em relação à audição, o som também exerce um valioso papel na percepção humana. Em síntese, a literatura revela que o nervo auditivo não vai diretamente ao cérebro, mas passa primeiramente pelo bulbo – sede das ações nervosas – seguindo o seu percurso ate o nível diencefálico – sede das emoções – e, mais adiante, atinge o córtex cerebral – sede das reações intelectuais. A interpretação musical envolve o sistema límbico, o tálamo, o hipotálamo e outras estruturas do tronco cerebral responsáveis pela modulação das emoções (Wisnik; 1989). Outros autores indicam que as propriedades físicas e objetivas dos sons estão associadas a determinadas sensações subjetivas da música e como os sons são produzidos e interpretados pelo cérebro (Roederer, 1998, Jourdain 1997). A música exerce um papel psicológico e é uma ferramenta para despertar as mais variadas sensações no público, como bem explicita sua utilização no cinema, por exemplo.

A música instaura personagens, acontecimentos marcantes nas narrativas a até pode carregar significados que perpassam gerações. A música sozinha já tem o poder de comunicação emocional, que pode ser otimizado quando amplamente integrada aos elementos de linguagem visual e ainda em associação com outros sentidos (Luz, Davino; 2009).

A música por si própria também pode desencadear imagens mentais ou reforçá-las, uma vez que alguns temas têm sido apontados como recorrentes às imagens, tais como cenas da natureza (sol, céu, oceano, plantas e animais), experiências extracorpóreas (flutuações, viagens cósmicas) e imagens religiosas (Deus, morte, julgamento), bastante comuns em exercícios de relaxamento (Osborne, 1981) e normalmente relatadas como produtoras de sensações de bem-estar. Talvez porque trate de valores universais e coletivos contidos no inconsciente sob a forma de categorias herdadas ou arquétipos, ou seja, imagens primordiais

que se propagam universalmente e irrompem de novo na existência por meio de uma função psíquica natural. À medida que os cérebros humanos são uniformemente diferenciados, na mesma medida a função mental possibilitada é coletiva e universal. Assim é que se explica o fato de que os processos inconscientes dos povos e raças, separados no tempo e no espaço, apresentam uma correspondência impressionante, que se manifesta, entre outras coisas, pela semelhante e fartamente confirmada presença de temas e formas mitológicas (Jung, 1987).

Se a estimulação isolada de cada um dos sentidos resulta em benefícios físicos e psicológicos, indagamos se a associação entre eles pode resultar em efeitos sinérgicos em relação às variáveis estudadas.

A abordagem multissensorial proposta tem como base que processos perceptivos, bem como de comportamento, são influenciados por entradas simultâneas de vários sentidos. Os seres humanos possuem um impressionante conjunto de sistemas sensoriais especializados que lhes permite monitorar simultaneamente uma série de estímulos ambientais. Este processamento "paralelo" de múltiplos estímulos não só aumenta a probabilidade de detectar um dado estímulo, mas também aumenta a probabilidade da sua identificação precisa. Em muitas circunstâncias, eventos são mais facilmente percebidos, têm menos ambiguidade e podem levar a uma resposta muito mais rápida quando sinalizados pela ação coordenada de várias modalidades sensoriais. Sistemas sensoriais têm evoluído para trabalhar em conjunto e, comumente, diferentes estímulos sensoriais que se originam a partir do mesmo evento são concordantes no espaço e no tempo. Neurônios multissensoriais do córtex participam de processos perceptivos, mnemônicos e associativos que servem para unir os componentes específicos de uma experiência multissensorial (Moser-Mercer,2005).

Este estudo busca explorar, portanto, lacunas ainda existentes na literatura no que diz respeito à eficácia de intervenções voltadas à redução do stress e promoção de bem-estar e autoestima da população-alvo, assim como promover a discussão e reflexão de questões relativas ao contato consigo mesmo, na perspectiva do autocuidado.

3. Justificativa e impactos esperados

Embora stress configure um termo em moda, pesquisadores indicam que o crescimento da atividade profissional no domínio do gerenciamento de stress não foi acompanhado por um aumento compatível de pesquisas sérias. A maioria dos programas existentes não foi avaliada nesse sentido. Parece haver dois problemas: o primeiro é que muitas intervenções não são avaliadas de forma sistemática e o segundo é que, em caso de avaliações, os planos de estudo são frequentemente caracterizados por falhas metodológicas graves. Apesar de haver uma pesquisa de intervenção metodológica e teoricamente mais confiável do que há vinte

anos, esse tipo de dado ainda se desenvolve pouco a pouco e só cobre uma minoria das atividades atuais de prevenção de stress. Por fim, esse tipo de pesquisa de avaliação continua tendo "um viés post hoc para o indivíduo" (Kompier, Kristensen; 2003).

Há escassez de estudos de intervenção. Mesmo na ampla literatura que indica o quanto os profissionais de enfermagem e de saúde em geral são sujeitos ao stress, pouco se tem estudado sobre formas de prevenção e ou eficácia de intervenções.

Na literatura encontramos estudos que tangenciam as questões de redução do stress e de outros sintomas por meio de técnicas de relaxamento, tais como a massagem, a música e a utilização de imagens mentais. Entretanto, a pesquisa na forma como está proposta para a população do estudo é inédita em nosso meio.

São resultados esperados do estudo:

- Que ao menos uma das intervenções se mostre efetiva para o manejo do stress e promoção da autoestima e bem-estar;
- Como os desfechos são a redução do stress, promoção de bem-estar e melhora da autoestima em mulheres profissionais de saúde, é possível que outras populações femininas na mesma faixa etária também tenham os mesmos benefícios, uma vez que os resultados observados serão mediados pelos sentidos humanos;
- Se os resultados esperados forem observados abre-se a perspectiva para a realização de novos estudos considerando outras faixas etárias e gênero.

As entregas deste projeto:

- Publicações científicas e divulgação em eventos científicos.

4. Casuística e Método

Desenho do estudo

Trata-se de um estudo clínico, controlado, aberto, randomizado, com abordagem mista (quantitativa e qualitativa).

A intervenção terá duração de trinta dias. Serão coletados dados no momento inicial do estudo (*baseline*), após 15 dias, após 30 dias (final da intervenção) e após follow-up de 30 dias (60 dias após o início do estudo).

Após a explanação dos objetivos do estudo e verificação dos critérios de inclusão e exclusão no estudo (Anexo II), se a voluntária decidir por participar da pesquisa, avaliará e assinará o

termo de consentimento livre e esclarecido TCLE (Anexo XX) e receberá as orientações em conformidade com o grupo para o qual foi aleatorizada. A aleatorização será feita pelo software Research Randomizer® para distribuição de 60 participantes para cada um dos quatro braços do estudo:

- Grupo 1) grupo controle
- Grupo 2) intervenção monossensorial (mediada pelo tato) representada pela atividade diária de hidratação corporal com um creme inodoro;
- Grupo 3) intervenção bissensorial (mediada pelo tato e olfato) representada pela atividade diária de hidratação corporal com um creme com fragrância;
- Grupo 4) intervenção multissensorial (mediada pelo tato, olfato, audição e visão) representada pela atividade diária de hidratação corporal com um creme com fragrância, após assistir um breve material audiovisual com cenas da natureza.

Aspectos Éticos

Todas as respostas coletadas no estudo serão confidenciais, de modo que cada participante seja identificada por uma codificação no banco de dados. As fichas de coleta de dados e demais documentos com informações do estudo serão armazenadas em armário com chave e os arquivos eletrônicos serão armazenados em um computador/arquivo protegido por senha, conhecida somente pelos pesquisadores. O projeto será submetido ao Comitê de Ética em Pesquisa institucional e a participação no estudo será feita mediante anuência em Termo de Consentimento Livre e Esclarecido.

Em conformidade com a Resolução 466/12 do Conselho Nacional de Saúde, as participantes serão informadas sobre o conteúdo e propósito da pesquisa e poderão abandonar o estudo em qualquer momento, sem que haja comprometimento para a participante.

Participantes do estudo

Após a aprovação pelo Comitê de Ética em Pesquisa institucional, a divulgação da pesquisa será iniciada, assim como a inclusão de pacientes nos quatro grupos de estudo, até que se alcance o total de 60 mulheres profissionais de saúde da Sociedade Beneficente Brasileira Israelita Albert Einstein (SBIBAE), em um total de 240 mulheres. O tamanho estimado para a amostra foi definido por conveniência, sem a utilização de cálculo teórico, tendo em vista que a relação entre o objeto e as intervenções do estudo não foram abordadas anteriormente na literatura na forma como proposta, de modo que não possuímos informação a priori sobre as estimativas de efeito esperadas no estudo.

A comunicação para recrutamento das participantes será feita pela área de comunicação interna institucional, por meio da intranet, e-mails e cartazes afixados em murais informativos da instituição com grande circulação de funcionárias (Anexo I).

Serão considerados os seguintes critérios de inclusão:

- Profissional da área da saúde;
- Adulto (18 a 60 anos);
- Sexo feminino;
- Atuação assistencial ou administrativa na SBIBAE;
- Participação voluntária;
- Assinatura no termo de consentimento livre e esclarecido.

Serão considerados critérios não inclusão as profissionais que:

- Avaliação dermatológica com achados relevantes diagnosticados pela dermatologista;
- Trabalhem no período noturno ou em períodos alternados (em virtude das sabidas alterações cronobiológicas que afetariam o estudo do cortisol, um dos desfechos considerados no estudo);
- Estejam amamentando;
- Estejam grávidas.

A utilização do seu creme de hidratação corporal habitual de uso diário não será considerada critério de exclusão, todavia, será realizada orientação específica para suspensão do mesmo durante a realização do estudo. O mesmo será aplicável ao uso de medicamentos que podem estar relacionados ou não com situações de estresse. O hidratante corporal permitido durante o estudo (Grupos II, III e IV) será o produto fornecido na 1ª visita.

Todas as avaliações com cada participante serão realizadas nas instalações físicas do Instituto Israelita de Ensino e Pesquisa Albert Einstein do Hospital Israelita Albert Einstein (IIEP), mediante agendamento realizado pela coordenadora de pesquisa clínica do Centro de Pesquisa clínica do IIEP-SBIBAE.

Na ocasião do primeiro contato com as voluntárias, o *check-list* para verificação dos critérios de inclusão e exclusão (Anexo II) e o Termo de consentimento livre e esclarecido – TCLE (Anexo XX) serão utilizados para a decisão sobre a inclusão ou não da voluntária no estudo.
Será realizado teste piloto para avaliação de ajustes nos procedimentos de operacionalização do estudo com 10 voluntárias.

Intervenção experimental

Para a hidratação será utilizado o produto Creme hidratante corporal (Grupos 3 e 4) e uma apresentação da mesma base sem fragrância para o grupo 2. Todas as embalagens não terão referência alguma que relacione à marca Natura ou qualquer outra marca.
O código da fórmula do creme hidratante corporal, a relação dos ingredientes e suas respectivas funções estão contidas no Anexo XXI. O código da fórmula deste estudo:1082.18602.2.

Modo de uso do creme hidratante corporal: Aplicação em todo o corpo, exceto no rosto. Uso diário.

Será solicitado a cada participante que selecione um horário do dia em que utilizará o hidratante sobre o corpo (exceto rosto), por um período de 30 dias. O participante deverá preencher diariamente um diário de uso domiciliar do produto durante o estudo.

Nos Grupos 2 e 3, a hidratação consistirá de leve automassagem corporal para distribuição e absorção do hidratante. As participantes deste grupo receberão orientações específicas, apresentadas no Anexo III.

Para o Grupo 4, será solicitado às participantes que antes e durante a execução da atividade de autocuidado, assistam um material audiovisual disponível no *tablet* que será fornecido a elas no início do estudo, ocasião em que assinarão um recibo de entrega do tablet e compromisso de devolução ao término do estudo (Anexo IV). Uma apresentação de *powerpoint* será disponibilizada no *tablet* com uma sequência de imagens da natureza e música com duração de dois minutos e quarenta segundos. O tablete não terá nenhuma outra função disponível que não a de reprodução das imagens selecionadas. As imagens foram selecionadas tendo como referência, além dos estudos apresentados inicialmente neste projeto (Ulrich, 1984; Marcus, Barnes; 1995), estudo não publicado realizado pelo patrocinador buscando alguns signos cromáticos, verbais, visuais, táteis e olfativos do algodão, por consultores com expertise em semiótica, uma vez que este signo está ligado ao creme hidratante corporal. A sequência de imagens está constituída por 17 fotografias, principalmente de flores e paisagens, apresentada no Anexo V. As orientações específicas das participantes do grupo 4 estão descritas no Anexo VI.

A análise semiótica retromencionada também serviu como referência para a sonorização, que recaiu sobre a canção tradicional irlandesa *Sweet Portaferry* (McGrath,1992), executada por Eve Watters no CD Quiet Visit – Celtic Harp (2005). Trata-se de uma melodia tradicional irlandesa, com arranjo para harpa, tonalidade em Mi bemol Maior, andamento lento (35 Beats/minuto), compasso composto (6/8), caráter melancólico e terno. Esta canção (Anexo VII), além de atender os parâmetros musicais de melodias que tem efeito relaxante (Nilsson 2010; Lai, Li; 2011), carrega no timbre da harpa, signos semelhantes aos do

algodão, como por exemplo, suavidade, acolhimento e cuidado (Ganzini, Rakoski, Cohn, Mularski; 2013)
Em casos de ocorrência eventual de alergias ou qualquer intercorrência em virtude do estudo, a participante será avaliada pelo dermatologista que acompanhará o estudo e se necessário for será encaminhada para atendimento e acompanhamento médico também no serviço de saúde de instituição conveniada, conforme benefício institucional da colaboradora, bem como será preenchida uma ficha de notificação de evento adverso (Anexo VIII), que será encaminhada para o Comitê de Ética em Pesquisa e acompanhada pela da equipe do projeto.

Ao término do estudo será de responsabilidade do patrocinador a retirada das amostras e encaminhamento para a central de resíduos da empresa.

Medidas de avaliação – Variáveis do Estudo

As principais variáveis estudadas serão: cortisol salivar, nível de stress percebido, autoestima, bem-estar subjetivo, experiência simbólica, percepção diária e global da experiência com o autocuidado proposto e adesão ao autocuidado após término do estudo.

O grupo controle será submetido às mesmas avaliações dos grupos de intervenção, com exceção do preenchimento do diário e follow-up. O grupo controle e os grupos de intervenção serão avaliados no *baseline*, em 15 dias, em 30 dias e após 30 dias de follow-up. Todas as participantes de todos os grupos passarão por uma avaliação realizada por uma dermatologista no início e ao término do estudo.

A frequência de cada avaliação específica está demonstrada no Anexo IX.

Na visita de baseline as participantes responderão a um questionário com informações socioeconômicas: idade, estado civil, número de filhos, crença espiritual, escolaridade, área de trabalho, tempo de formado, tempo de atuação na área, horário de trabalho, renda, presença de dificuldades financeiras ou físicas ou emocionais, além de práticas e frequência de atividades de autocuidado. Estas informações permitirão a caracterização das pacientes e verificação da homogeneidade dos grupos formados a partir da aleatorização (Anexo X). A relação desta aleatorização estará contida na ficha de randomização (Anexo XXII).

As pacientes farão a coleta de cortisol salivar no laboratório clínico da instituição. O cortisol é um esteróide produzido no córtex da suprarenal e está envolvido na resposta ao stress. Trata-se de uma medida objetiva para compor a avaliação do nível de stress. A concentração de cortisol salivar apresenta o mesmo ritmo circadiano que o cortisol sérico, assim, encontramos níveis mais elevados em torno das 8 horas da manhã e mínimos durante a madrugada (entre 0

e 4 horas). A concentração de cortisol na saliva representa a fração livre do cortisol e reflete a concentração no sangue, entretanto, níveis muito altos no soro levam a níveis desproporcionais na saliva. O método utilizado no estudo tem a vantagem de ser nãoinvasivo, indolor e seguro. A coleta é feita mediante a utilização de tubos plásticos com algodão específico para a coleta de saliva. Este dispositivo permite uma coleta fácil e limpa Os exames serão coletados no laborarório de análises clínicas do Einstein, no Bloco A1 – Intermediário 3 entre 8 e 9h da manhã.

A medida do cortisol salivar independe do fluxo de saliva e das flutuações da transcortina. As amostras de saliva são obtidas por procedimento simples, não invasivo, livre de stress, podendo ser realizadas por pessoas não treinadas em ambulatório ou na própria residência do paciente. Estas amostras podem ser coletadas muitas vezes ao dia, permitindo a avaliação dinâmica da secreção de cortisol livre. Além disso, as amostras do cortisol salivar são estáveis em temperatura ambiente por uma semana e podem ser transportadas ao laboratório pelo correio ou pelo portador, sem nenhuma perda da atividade do cortisol (Castro; 2003).

O volume de saliva obtido com o procedimento corresponde normalmente entre 0.5 a 1.5 ml. As coletas de saliva serão realizadas apenas uma vez a cada visita.

As participantes responderão ainda, aos seguintes inventários:

- Inventário de Sintomas de Stress para adultos de LIPP – ISSL (Anexo XI)
- Lista de Sintomas de Stress para Adultos - LSS (Anexo XII)
- Escala de autoestima (Anexo XIII)
- Escala de satisfação com a vida (Anexo XIV)
- Escala de afetos positivos e negativos (Anexo XV)
- Diário dos Grupos 2 e 3 (Anexo XVI)
- Diário do Grupo 4 (Anexo XVII)

INVENTÁRIO DE SINTOMAS DE STRESS PARA ADULTOS DE LIPP (ISSL): (Anexo XI)

O Inventário de Sintomas de Stress para Adultos, padronizado por Lipp e Guevara (1994), baseou-se num modelo trifásico desenvolvido por Selye. O ISSL é composto por três quadros (Q) que se referem às quatro fases do stress, divididos de forma temporal em sintomas das últimas 24 horas (Q1 – 15 sintomas da fase de alerta), última semana (Q2 – 15 sintomas da fase de resistência e quase exaustão) e último mês (Q3 – 23 sintomas da fase de exaustão). A sintomatologia é dividida em física e psicológica, correspondentes às manifestações mais frequentes do stress sentidas pelos indivíduos que vivenciam uma situação potencialmente estressora. No ISSL, o diagnóstico positivo é dado a partir da soma dos sintomas de cada quadro do inventário.

Este instrumento fornece uma medida objetiva da sintomatologia do stress em jovens acima de 15 anos e adultos. Sua aplicação leva aproximadamente 10 minutos e pode ser realizada individualmente ou em grupos de até 20 pessoas. É formado por três quadros referentes às fases do stress. O primeiro quadro, composto de 15 itens refere-se aos sintomas físicos ou psicológicos que a pessoa tenha experimentado nas últimas 24 horas. O segundo, composto de dez sintomas físicos e cinco psicológicos, está relacionado aos sintomas experimentados na última semana. E o terceiro quadro, composto de 12 sintomas físicos e 11 psicológicos, refere-se a sintomas experimentados no último mês. Alguns dos sintomas que aparecem no quadro 1 voltam a aparecer no quadro 3, mas com intensidade diferente. No total, o ISSL apresenta 37 itens de natureza somática e 19 psicológicas, sendo os sintomas muitas vezes repetidos, diferindo somente em sua intensidade e seriedade. A fase 3 (quase exaustão) é diagnosticada na base da frequência dos itens assinalados na fase de resistência.
Obtem-se a classificação para cada fase da seguinte forma:
Alerta - se maior que 6 sintomas;

Resistência - se maior que 9 sintomas;

Exaustão - se maior que 8 sintomas.

LSS – LISTA DE SINTOMAS DE STRESS (FERREIRA, VASCONCELOS, MARQUES; 2002) (Anexo XII)
A Lista de Sintomas de Stress é constituída por 59 itens redigidos em primeira pessoa, sobre sintomas de estresse psicofisiológico e social. O sistema de avaliação dos sintomas de stress envolve a identificação da frequência em uma escala tipo Likert de quatro pontos, graduada da seguinte forma: zero (nunca), um (raramente), dois (frequentemente), e três (sempre). Os escores dos índices indicativos da frequência de estresse variam de zero a 177, sendo que os escores mais baixos indicam ausência ou baixa frequência de manifestação de sintomas, enquanto escores mais altos indicam alta frequência dessa manifestação, conforme classificação do stress a seguir:

0 – 11 pontos –nulo

12 – 28 pontos – baixo

29 – 60 pontos – nível médio

61 – 120 pontos – alto

Acima de 120 pontos – altíssimo

ESCALA DE AUTOESTIMA DE ROSENBERG – EAR (Anexo XIII)

A Escala de Autoestima desenvolvida por Rosenberg (1989) é uma medida unidimensional constituída por dez afirmações relacionadas a um conjunto de sentimentos de autoestima e autoaceitação que avalia a autoestima global. Os itens são respondidos em uma escala tipo Likert de quatro pontos variando entre concordo totalmente, concordo, discordo e discordo totalmente. Neste estudo será utilizada a versão adaptada para o português por Hutz (2000), cujos resultados iniciais já indicavam a unidimensionalidade do instrumento e características psicométricas equivalentes às encontradas por Rosenberg (1989). Seu preenchimento aproximado é de dez minutos. O nível de autoestima é classificado da seguinte forma:

Elevada autoestima – maior que 30;

Média autoestima – entre 20 e 30;

Baixa autoestima – menor 20.

ESCALAS DE BEM-ESTAR SUBJETIVO (Anexo XIV)

A Escala de Satisfação com a Vida (Diener et al., 1985) é composta por cinco itens que avaliam um dos componentes cognitivos do bem-estar subjetivo (por exemplo, na maioria dos aspectos, minha vida é próxima ao meu ideal; se pudesse viver uma segunda vez, não mudaria quase nada na minha vida). Os participantes baseiam suas respostas em uma escala de 7 pontos, com os extremos 1 (discordo totalmente) e 7 (concordo totalmente). traduzida e adaptada para o português brasileiro por Gouveia, Taciano, Milfont, Fonseca e Coelho (2009).

Os resultados mensurados por esta escala variam de 5 a 35, sendo que quanto maior o escore, mais satisfeito (20 é o ponto considerado neutro).

ESCALA DE AFETOS POSITIVOS E NEGATIVOS (Anexo XV)

A Escala de Afeto Positivo e Negativo (EAPN) originalmente composta por Diener e Emmons (1985), que selecionaram nove adjetivos para acessar a valência de afetos. Para os afetos positivos, consideraram-se os adjetivos: alegre, feliz, satisfeito e divertido; ao passo que para os negativos, os autores selecionaram: deprimido, preocupado/ansioso, frustrado, irritado/hostil e infeliz. O instrumento tem como objetivo avaliar quanto o participante tem experienciado cada uma das emoções nos últimos dias, sendo seus itens respondidos em uma escala de sete pontos variando entre 1 = Nada e 7 = Extremamente.

Neste estudo seguimos a adaptação realizada por Chaves (2003) que verificou a validade e a precisão da Escala de Afetos Positivos e Negativos em uma amostra de 306 pessoas da população geral da cidade de João Pessoa – PB,.no qual, a fim de equilibrar o número de itens para ambos os fatores da escala, foi acrescentado o adjetivo “otimista” para os afetos positivos.

DIÁRIO (Anexos XVI e XVII)

Para apreensão da percepção da experiência diária e global da experiência será fornecido um diário em formato brochura para cada participante, onde ela irá registrar a data da última menstruação e a data do primeiro dia do ciclo (caso a voluntária fique menstruada no meio da pesquisa) e diariamente os seguintes dados: data, horário de início e término do autocuidado, local escolhido para o autocuidado e um breve relato de sensações, emoções e sinais clínicos (caso for observado algum sinal) decorrentes da experiência naquele dia e decorrentes da experiência após os 30 dias. Para a avaliação global da experiência, será solicitado às participantes que respondam à seguinte questão aberta: “*Como foi esta experiência de autocuidado para você durante este mês*?”

O diário do grupo 4 será idêntico o dos grupos 2 e 3 exceto por ter três questões específicas sobre o material audiovisual e o lembrete para devolução do tablet.

Embora o diário seja um instrumento de coleta de dados mais utilizado na área educacional, será utilizado neste estudo porque além da análise que possibilita, favorece a reflexão imediata da participante sobre a prática do autocuidado realizada, o que reforça a percepção da intervenção, uma vez que o estudo busca a adesão à prática do autocuidado.

Para Zabalza (2004), os diários são usados em distintos contextos de pesquisa por terem a capacidade de responder à dupla exigência metodológica de “centrar as análises em situações concretas, integrando a dupla dimensão referencial e expressiva dos fatos”. Em outras palavras, os diários permitem a objetividade da situação por meio da versão subjetiva que os sujeitos dão a ela e subjetividade da situação por meio dos dados objetivos que o próprio documento pessoal proporciona. Possibilita a presença de referentes afetivos e cognitivos e cria um espaço para a constituição de subjetividades.

DESENHO (Anexo XVIII)

Para apreender a experiência simbólica será solicitado às participantes que façam um desenho no início e término do estudo que represente a seguinte pergunta: *Como você está se sentindo tendo como referência sua última semana?* Para tanto, serão fornecidos lápis coloridos e papel sulfite tamanho A4.

O desenho é uma técnica de representação gráfica, é uma das formas de comunicação não verbal e, de acordo com diversos estudiosos, a mais antiga. Linhas, curvas e cores divulgam histórias, culturas, sociedades e até mesmo a personalidade. Os desenhos revelam sentimentos, desejos, pensamentos e ação. Desenhar é projetar-se no espaço com apropriação do conhecimento em si e do meio com o qual se interage. É uma forma de mostrar como se percebe a vida. Nos vários campos da ciência, o desenho apresenta relevância para a humanidade. Seja como elemento precursor da escrita (ideográfica e fonética), destacando sua importância histórico cultural, ou na avaliação do desenvolvimento infantil (para maior compreensão sobre as fases de vida das crianças), na identificação de personalidade para intervenções psicológicas, na avaliação de habilidades cognitivas; o desenho tem se destacado como um importante instrumento metodológico. Souza Filho (1998) esclarece ainda que, como uma das primordiais formas de linguagem, o desenho revela aspectos que permeiam o mundo, as experiências, a memória, a imaginação. Para ele, o desenho "é um grande esforço de abstração, a partir da socialização e da comunicação, na tentativa de fixar, em um suporte físico duradouro, situado fora do seu próprio cérebro, fragmentos de suas percepções e experiências no mundo".

FOLLOW-UP (Anexo XIX)
A título de follow-up, após trinta dias do término do estudo, as medidas de avaliação (exceto o diário) serão novamente aplicadas, acrescidos da seguinte questão para verificar a adesão ao autocuidado após término do estudo: *Você tem exercitado o autocuidado? Se sim, como?*

Análise dos Dados:

As variáveis socioeconômicas e as variáveis e interesse do estudo avaliadas? nas visitas de baseline, 15 dias, 30 e 60 dias serão descritas por frequências absolutas e porcentagens no caso de variáveis qualitativas ou por medidas resumo como média, mediana, desvio padrão, intervalos interquartis e valores mínimos e máximos no caso das variáveis numéricas.

Os resultados dos instrumentos serão avaliados em categorias, definidas nos estudos que validaram os instrumentos, de modo a facilitar a interpretação dos resultados.

A avaliação da relação entre os níveis de bem estar, autoestima e níveis de stress será feita por tabelas de dupla entrada e testes de associação de Qui-quadrado de Pearson.

A comparação dos grupos quanto aos níveis de stress, bem estar e autoestima será feita por equações de estimação generalizadas, contemplando a correlação entre as medidas realizadas

ao longo do tempo, ou por técnicas não paramétricas de análise de variância para medidas repetidas.

Os desenhos serão analisados quanto às cores utilizadas classificadas segundo o espectro de luz, em cores quentes (Vermelho, laranja, amarelo, marrom e nuances intermediárias) e frias (Verde, azul, cinza, violeta e nuances intermediárias). As cores e os objetos representados serão analisados utilizando-se um Dicionário de Símbolos (Chevalier, Gheerbrant, 2001), adotada em estudos anteriores (Leão, 2005).

As respostas qualitativas serão submetidas à análise de conteúdo, conforme Bardin (1977), enquanto um conjunto de técnicas de análise das comunicações, que utiliza procedimentos sistemáticos e objetivos de descrição do conteúdo das mensagens.

5. Equipe do estudo

O estudo será coordenado pela pesquisadora proponente e a equipe técnica será definida incluindo profissionais de saúde do Hospital Israelita Albert Einstein (enfermeiro, psicólogo, estatístico) e pesquisadores da Natura.

6.Referencias

Albuquerque As, Trócolli BT. Desenvolvimento de uma escala de bem-estar subjetivo. Psicologia:Teoria e Pesquisa 2004; 20(2): 153-64.

Baggio MA, Formaggio FM. Profissional de enfermagem: compreendendoo autocuidado. Revista Gaúcha de Enfermagem 2007;28(2):233-41.

Bardin L. Análise de conteúdo. Lisboa: Edições 70; 1977.

Barros DS, Tironi MOS, Nascimento Sobrinho CL, Neves FS, Bitencourt AGV, Almeida AM. Médicos plantonistas de unidade de terapia intensiva: perfil sócio-demográfico, condições de trabalho e fatores associados à síndrome de burnout. Rev Bras Ter Intensiva 2008; 20(3): 235-240.

Blegen, M. A. (1993). Nurses' job satisfaction: a meta-analysis of related variables.Nursing Research, 42, 36-41.

Borda, R. C., & Norman, I. J. (1997). Factors influencing turnover and absenceof nurses: a research review. International Journal of Nursing Studies, 34,385-394.

Cascio, W. F. (1995). Whiter industrial and organizational psychology in a mchanging world of work? American Psychologist, 50, 928-939.

Castro M, Moreira AC. Análise crítica do cortisol salivar na avaliação do eixo hipotálamo-hipófise-adrenal. Arq Bras Endocrinol Metab 2003; 47(4): 358-367.

Castro-Sanchez AM, Moreno-Lorenzo C, Matarán-Peñarrocha G, Ferriche-Fernández-Castanys B, Sanchez NR, Sanchez MMJ. Eficacia de un programa de masaje y ejercicio sobre el índice tobillo/brazoy presión sanguíneaen pacientes con diabetes mellitus tipo 2 y enfermedad arterial periférica: ensayo clínico aleatorizado. Med Clin (Barc). 2010;134(3):107-10.

Cerchiari EAN, Caetano D, Faccenda O. Prevalência de transtornos mentais menores em estudantes universitários. Estudos de Psicologia 2005; 10(3): 413-20.

Chaves SSS. Valores como preditores do bem-estar subjetivo. 2003. Dissertação deMestrado. Universidade Federal da Paraíba. Centro de Ciências Humanas, Letras e Artes.Departamento de Psicologia. Mestrado em Psicologia Social. 2003.

Chevalier J, Gheerbrant A. Dicionário de Símbolos. 16ª ed. Rio de Janeiro: José Olympio; 2001.

Cooper, C. L., Dewe, P. J., & O`Driscoll, M. O. (2001). Organizational stress: a

review and critique of theory, research, and applications. London: Sage.

Costa ALS, Polak C. Construção e validação de instrumento para avaliação de stress em estudantes de enfermagem (AEEE). Rev Esc Enferm USP 2009; Número especial(43): 1017-1026.

Davidson JL. Aromatherapy & work related stress. The International Journal of Aromatherapy 2002; 12(3): 145-51.

Diener E, Emmons RA, Larsen RJ, Griffin S. The Satisfaction with Life Scale. Journal of Personality Assessment 1985; 49: 71-5.

Ferreira EAG, Vasconcellos EG, Marques AP. Assessment of pain and stress in fibromyalgia patients. Rev Bras Reumatol 2002; 42-104-10.

Freire T, Tavares D. Influencia da autoestima, da regulação emocional e do gênero no bem-estar subjetivo e psicológico de adolescentes. Rev Psiq Clín. 201;38(5):184-8.

Field T, Ironson G, Scafidi F, Nawrocki T, Gonclaves A, Burman I. Massage therapy reduces anxiety and enhances EEG pattern of alertness and math computations. Int J Neurosci. 1996; 86(3-4):197-205.

Fogg, BJ. Persuasive Technology: Using Computers to Change What We Think and Do. São Francisco: Morgan Kaufmann; 2002.

Freire T, Tavares D. Influencia da autoestima, da regulação emocional e do gênero no bem-estar subjetivo e psicológico dos adolescentes. Rev Psiq Clin 2011; 38(5): 184-8.

Galindo SG, Moreno IM, Muñoz JG. Prevalencia de Ansiedad y Depresión en una Población de Estudiantes Universitarios: Factores Académicos y Sociofamiliares Asociados. Cínica y Salud 2009, 20(2): 177-187.

Ganzini L, Rakoski A, Cohn S, Mularki RA. Family members' views on the benefits of harp music vigils for terminally-ill or dying loved ones. Palliat Support Care 2013; 16:1-4.

Giacomoni CH, Hutz CS.. A mensuração do bem-estar subjetivo: escala de afeto positivo e negativo e escala de satisfação de vida. Pôster apresentado no Congresso da Sociedade Interamericana de Psicologia, São Paulo, SP; 1997.

Giacomoni CH. Bem-estar subjetivo: Em busca da Qualidade de Vida.Temas em Psicologia 2004; 12: 43-50.

Gouveia VV, Taciano L, Milfont TL, Fonseca PN, Coelho JAP. Life satisfaction in Brazil: Testing the psychometric properties of the satisfaction with life scale (SWLS) in five brazilian samples. Social Indicators Research 2009; 90: 267–277.

Guerrer FJL, Bianchi ERF. Estresse dos enfermeiros atuantes em uti nas regiões do Brasil. Enfermería Global 2011, 22: 1-9.

Hutz CS. Adaptação da escala de autoestima de Rosenberg. Manuscrito não publicado. Universidade Federal do Rio Grande do Sul: Porto Alegre; 2000.

Imamura M, Furlan AD, Dryden T, Irvin E. Evidence-informed management of chronic low back pain with massage TheSpine Journal 2008;(8): 121–133.

Jourdain R. Cérebro e êxtase: como a música captura nossa imaginação. Rio de Janeiro: Objetiva; 1997.

Jung CG. O eu e o inconsciente. Trad. de Dora Ferreira da Silva. Petrópolis: Vozes; 1987.

Kalman BA, Grahn RE. Measuring salivary cortisol in the behavioral neuroscience laboratory. J Undergrated NeuroscienceEducation 2004; 2(2): A41-A49.

Kirschbaum C, Hellhammer DH. Salivary cortisol in psychoneurondocrine research: recent developments andapplications. Psychoneuroendocrinology 1994; 19(4): 313-33.

Kompier M A J, Kristensen TS. As intervenções em stress organizacional. Cad. psicol. soc. trab. 2003 periódico na Internet. citado 2012 Out 19. Disponível em: http://www.revistasusp.sibi.usp.br/scielo.php?script=sci_arttext&pid=S15163717200300020000 4&lng=pt.

Lai HL, Li YM. The effect of musico n biochemical markers and self-perceived stress among first-line nurses: a randomized controlled crossover trial. J Adv Nurs 2011. 67(11): 2414-24.

Leão ER, Silva MJP da. The relationship between music and musculoskeletal chronic pain. Online Brazilian Journal of Nursing (OBJN-ISSN 1676-4285) [online] 2005 April; 4(1) Available in: www.uff.br/nepae/objn401leaoetal.htm

Lipp MEN, Guevara AJH. Validação empírica do Inventário de Sintomas de Stress. Estudos de Psicologia 1984; 11(3): 4349.

Lipp MEN. Manual do inventário de sintomas de stress para adultos. São Paulo: Casa do Psicólogo; 2000.

Luz MC, Davino GE. Música, sedução e comunicação. In: Leão ER. (org) Cuidar de pessoas e música: uma visão multidisciplinar. São Caetano do Sul: Yendis; 2009. p. 6793.

Martino MMF, Misko MD. Estados emocionais de enfermeirosno desempenho profissionalem unidades críticas. Rev Esc Enferm USP2004; 38(2):161-7.

Matos FJA. Farmácia Viva: sistema de utilização de plantas medicinais projetado para pequenas comunidades. Fortaleza: Editora UFC; 2002.

Marcus CC, BarnesM. Gardens in healthcare facilities: uses, therapeutic benefits, and design recommendations.California: The Center for Health Design; 1995.

McGrath M. My gentle harp: Irish fold tunes. Dublin: Stepaside; 1992.

Moraska A et al. Physiological Adjustments to Stress Measures Following Massage Therapy: A Review of the Literature. Evid Based Complement Alternat Med. 2010 December; 7(4): 409–418.

Moyer CA, Rounds J, Hannum JW. A MetaAnalysis of Massage Therapy Research. Psychol Bull. 2004; 130(1):3–18.

Moser-Mercer B.Remote Interpreting: Issues of Multi-Sensory Integration in a Multilingual Task./ Meta: Translators' Journal 2005; 50(2): 727-738.

Negeliskii C, Lautert L. Estresse laboral e capacidade para o trabalho de enfermeiros de um grupo hospitalar. Rev. Latino-Am. Enfermagem. [periódico na Internet]. maio-jun 2011 [acesso 22 jun 2013];19(3):[8 telas]. Disponível em: http://www.scielo.br/pdf/rlae/v19n3/ pt_21.pdf

Nilsson U. Music: a nursing intervention. European J Cardiovasc Nurs 2010; 10:73-4.

Orem DE.Nursing – concepts of practice. 5 ed. St. Louis: Mosby; 1995.

Osborne JW. The mapping of thoughts, emotions, sensations, and images as response to music. J Mental Imagery 1981; 5(4): 133-36.

Páfaro RC, Martino MMF. Estudo do estresse do enfermeiro com dupla jornada de trabalho em um hospital de oncologia pediátrica de Campinas. Rev Esc Enferm USP 2004; 38(2):152-60.

Pert CB. Molecules of emotion. New York: Scribner; 2003.

Roederer JG. Introdução à psicofísica da música. São Paulo: EDUSP; 1998.

Rosenberg M. Society and the adolescent self-image. Revised edition. Middletown, CT: Wesleyan University Press; 1989.

Souza Filho EB (1998) – Semiótica Russa _ Desenho como sistema modelizante.

Disponível em http://www.pucsp.br/pos/cos/cultura/desenho.htm acesso em 20 jun 2013.

Rosenberg M. Society and the adolescent self-image. Revised edition. Middletown, CT: Wesleyan University Press; 1989.

Souza Filho EB (1998) – Semiótica Russa _ Desenho como sistema modelizante.

Disponível em http://www.pucsp.br/pos/cos/cultura/desenho.htm acesso em 20 jun 2013.

YC Tsai, Liu CH. Factors and symptoms associated with work stress and health-promoting lifestyles among hospital staff: a pilot study in Taiwan. . BMC Health Services Research 2012; 12:199.

Ulrich R. View through a window may influence recovery from surgery. Science1984; 224: 420-21.

Watson D, Clark L A, Tellegen A.Development and Validation ofBrief Measures of Positive and Negative Affect: The PANAS Scales. Journal of Personality and Social Psychology 1988; 54: 1063-1070.

Wisnik JM. O som e o sentido. São Paulo: Cia das Letras; 1989.

Zabaoza MA. Diários de aula. São Paulo: Artmed, 2004.

7. Cronograma

	2013					2014											
Etapas	ago	set	out	nov	dez	Jan	fev	mar	abr	mai	jun	jul	ago	set	out	nov	dez
Avaliação jurídica	x	x	x	x													
Assinatura do contrato Natura/Einstein					x												
submissão ao CEP e avaliação ética					x			x									
planejamento/infra-estrutura					x	X	x	x									
desenvolvimento do material audiovisual					x	X	x	x									
seleção das bolsistas de IC						X	x										
divulgação interna da pesquisa									x	x							
Recrutamento										x	x	x	x	x	x		
coleta de dados												x	x	x	x	x	
follow-up													x	x	x	x	x
análise e interpretação dos dados														x	x	x	x
elaboração do relatório final																	x
divulgação da pesquisa																	x

8. Orçamento estimado adequado aos objetivos do projeto:

Despesas	Quantidade	Valor Unitário	Total
Teste cortisol salivar	960	110,00	105.600,00
Diário de Campo individual	240	3,00	720,00
Material audiovisual	1	10.000,00	10.000,00
Diária especialista	2	1.000,00	2.000,00
Cópias dos instrumentos de coleta	6240	0,50	3.120,00
Laptops	2	1.500,00	3.000,00
Pacote Office	2	1.400,00	2.800,00
Sulfite (pacote)	10	15,00	150,00
Lápis de cor (caixa)	6	10,00	60,00
Cartucho de impressora	5	66,00	330,00
Bolsista 1 IC	14	615,00	8.610,00
Bolsista 2 IC	14	615,00	8.610,00
Divulgação do projeto	1	10.000,00	10.000,00
Avaliação Dermatológica*	480	75,00	36.000,00
**Total**			**191.000,00**

Todos os custos do projeto serão cobertos pela Natura, tendo em vista esta pesquisa ter sido vencedora do Edital NaturaCampus/2013. Os tablets também serão fornecidos diretamente pela Natura, por isso não estão contemplados no presente orçamento.

ANEXO I – Comunicado para Recrutamento

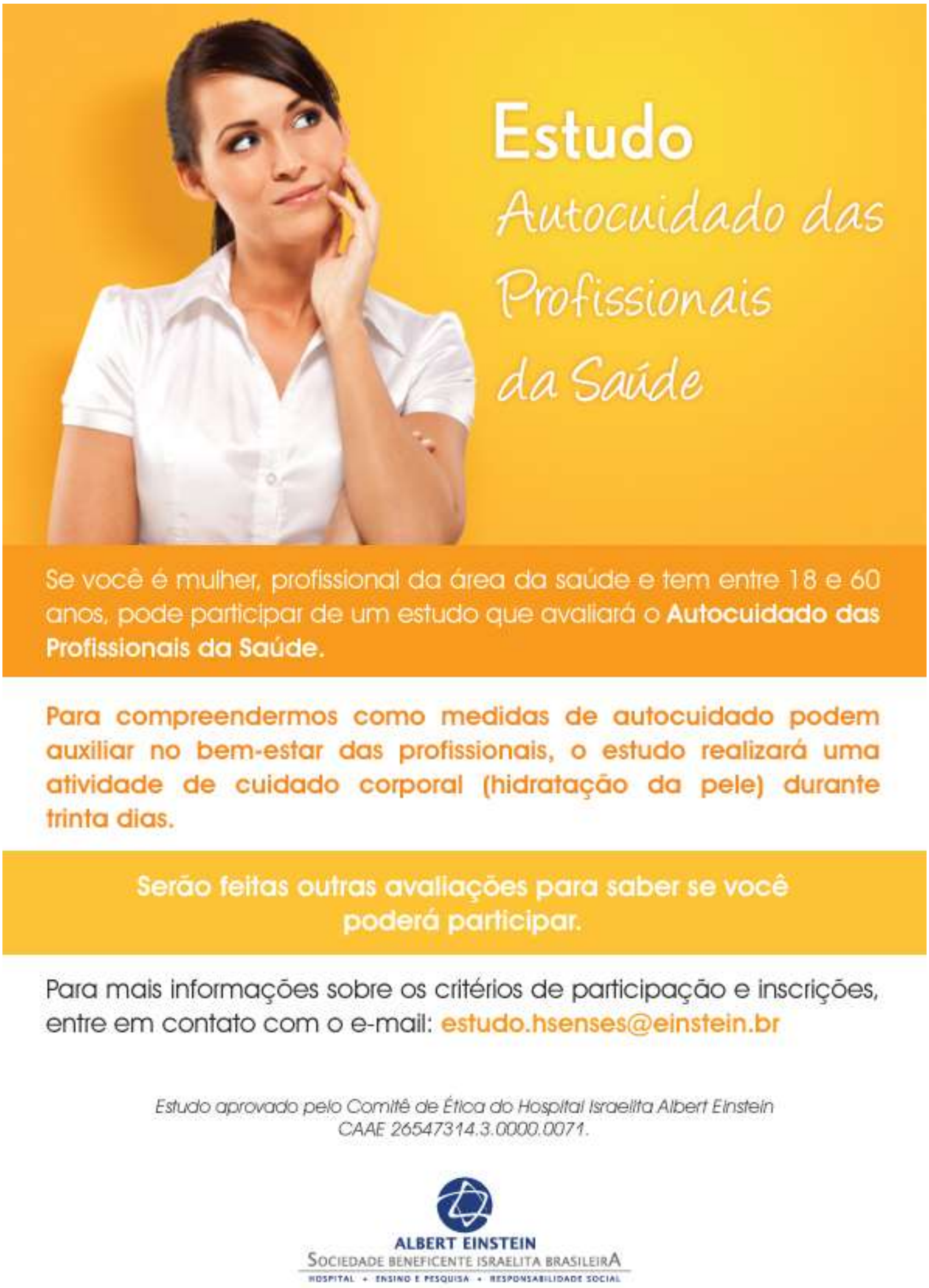

II – Check-list para verificação de critérios de inclusão e exclusão no estudo

Nome:____

Serão considerados os seguintes **critérios de inclusão**

Critérios de Inclusão	SIM
Profissional da área da saúde	( )
Adulto	( )
Sexo feminino	( )
Atuação assistencial ou administrativa	( )
Participação voluntária	( )
Assinatura no TCLE	( )

Serão considerados **critérios de não inclusão** as profissionais que apresentarem:

Critérios de Não inclusão	NÂO
Avaliação dermatológica com achados relevantes diagnosticados pela dermatologista	( )
Trabalho no período noturno ou em períodos alternados	( )
Amamentação	( )
Gravidez	( )

Liberada pela dermatologista para participar da pesquisa:

( ) Sim

( ) Não

Registro de medicamentos em uso:

____

____

Carimbo e assinatura da dermatologista

____

____

Nome da responsável pelo recrutamento

Anexo III – Orientação às participantes dos Grupos 2 e 3

Obrigada por ser voluntária em participar do estudo

Orientações importantes:

Obrigada por ser voluntária em participar do estudo

- Você está recebendo um creme hidratante corporal para uma atividade de autocuidado por um período de 30 dias. Esse produto não pode ser compartilhado com outras pessoas. Somente você poderá utilizá-lo neste período.

- Caso você esteja utilizando algum outro creme, você terá que suspendê-lo por 30 dias, ou seja, o tempo de duração do estudo.

- O frasco fornecido é suficiente para uso durante 30 dias. Porém, caso você perceba que o produto irá acabar antes desse prazo, entre em contato com a equipe do estudo para que um novo frasco seja providenciado a fim de evitar interrupções. Ao final do estudo você irá devolver o(s) frasco(s) independente da quantidade residual.

- Você irá escolher um horário durante seu dia para o momento de autocuidado. Neste momento você irá aplicar o creme hidratante corporal que estamos lhe fornecendo.

- Ao terminar de aplicar o produto você irá registrar no seu diário as seguintes informações: data, horário, tempo de duração do autocuidado localização física onde você realizou o autocuidado e as sensações e ou emoções proporcionadas pela experiência que acabou de ter e que você queira descrever.

- Caso você venha a apresentar qualquer tipo de alergia, vermelhidão da pele, coceira, ou qualquer manifestação que lhe chame a atenção, você deverá suspender a utilização do produto registrar no diário de uso em domicilio e entrar em contato com a equipe do estudo para receber as devidas orientações e ser avaliada por um médico dermatologista.

- Se por ventura, você se esquecer de usar o produto durante um dia registre esta informação no diário, bem como a razão pela qual você deixou de fazer o seu autocuidado. Mas faça o possível para cumprir essa prática por 30 dias consecutivos.

- Suas próximas avaliações serão em 15 dias, 30 dias e 60 dias, conforme as datas abaixo, no IIEP – 2º subsolo do Bloco A:

___/___/___   Horário: ______
___/___/___ Horário:______
___/___/___ Horário: ______

Nossos telefones: 11 973801412 ou 21511032.
Eliseth Leão
Pesquisadora Responsável do estudo H-SENSES

Anexo IV

Recibo de entrega do tablet e compromisso de devolução (via participante)

Eu, ____, RG: estou recebendo um tablet identificado pelo nº ______________________________em perfeitas condições de uso e me comprometo a devolvê-lo ao final deste estudo, na data de avaliação de 30 dias.

Data da entrega: ____/____/____

Assinatura do participante

✂ -

Recibo de entrega do tablet e compromisso de devolução (via equipe do estudo)

Eu, ____, RG: ___________estou recebendo um tablet identificado pelo nº ______________________________em perfeitas condições de uso e me comprometo a devolvê-lo ao final deste estudo, na data de avaliação de 30 dias.

Telefone: ____

Endereço:____

Data da entrega: ____/____/____

Assinatura do participante

Anexo V – Sequência de fotografias da natureza
(as fotos utilizadas estarão em alta resolução)

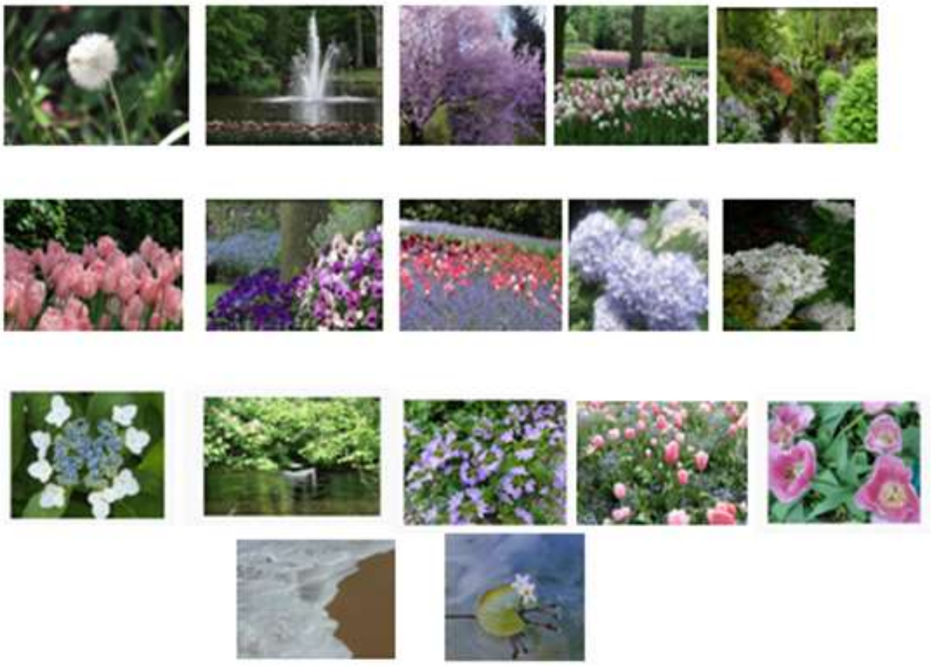

Anexo VI – Orientações às participantes do Grupo 4

Obrigada por ser voluntária em participar do estudo

Orientações importantes:

- Você está recebendo um creme hidratante corporal para uma atividade de autocuidado por um período de 30 dias. Esse produto não pode ser compartilhado com outras pessoas. Somente você poderá utilizá-la neste período.

- Caso você esteja utilizando algum outro produto, você terá que suspendê-lo por 30 dias, ou seja, o tempo de duração do estudo.

- O frasco fornecido é suficiente para uso durante 30 dias. Porém, caso você perceba que o produto irá acabar antes desse prazo, entre em contato com a equipe do estudo para que um novo frasco seja providenciado a fim de evitar interrupções. Ao final do estudo você irá devolver o(s) frasco(s) independente da quantidade residual no frasco. Você também irá devolver o tablet, que será entregue hoje, após a assinatura do recibo de entrega e compromisso de devolução.

- Você irá escolher um horário no seu dia para um momento de autocuidado. Neste momento você irá hidratar o seu corpo com o produto que estamos lhe fornecendo.

- Mas, antes de aplicar o creme hidratante corporal você irá assistir um material audiovisual, preparado especialmente para este seu momento e que tem a duração de 2 minutos e quarenta segundos. Este material está contido no tablet que lhe fornecemos, para acessá-lo basta abrir o link.

- Assista ao vídeo completo por uma vez, ao terminar a primeira exibição, inicie a aplicação do creme hidratante corporal, mantendo o vídeo em execução.

- Você irá registrar no seu diário as seguintes informações: data, horário, tempo de duração do autocuidado, localização física onde você realizou o autocuidado e as sensações e ou emoções proporcionadas pela experiência que acabou de ter e que você queira descrever.

- Caso você venha a apresentar qualquer tipo de alergia, vermelhidão da pele, coceira, ou qualquer manifestação que lhe chame a atenção, você deverá suspender a utilização do produto registrar no diário de uso em domicílio e entrar em contato com a equipe do estudo para receber as devidas orientações e ser avaliada por um médico dermatologista.

- Se por ventura, você se esquecer de usar a loção cremosa durante um dia registre isso no diário, bem como a razão pela qual você deixou de fazer o seu autocuidado. Mas faça o possível para cumprir essa prática por 30 dias consecutivos.

- Suas próximas avaliações serão em 15 dias, 30 dias e 60 dias, conforme as datas abaixo, no IIEP – 2º subsolo do Bloco A:
___/___/___ Horário: ______
___/___/___ Horário:______
___/___/___ Horário: ______

Nossos telefones: 11 973801412 ou 21511032.
Eliseth Leão
Pesquisadora Responsável do estudo H-SENSES

Anexo VII – Sweet Portaferry - Partitura

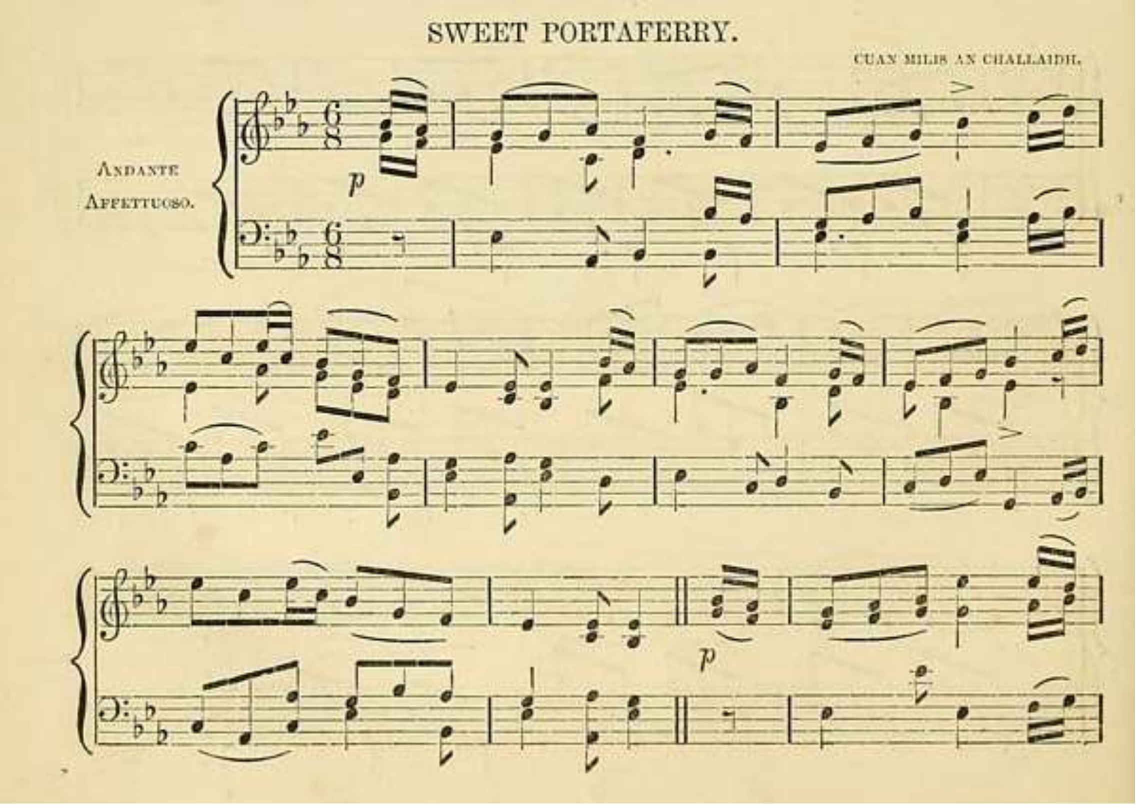

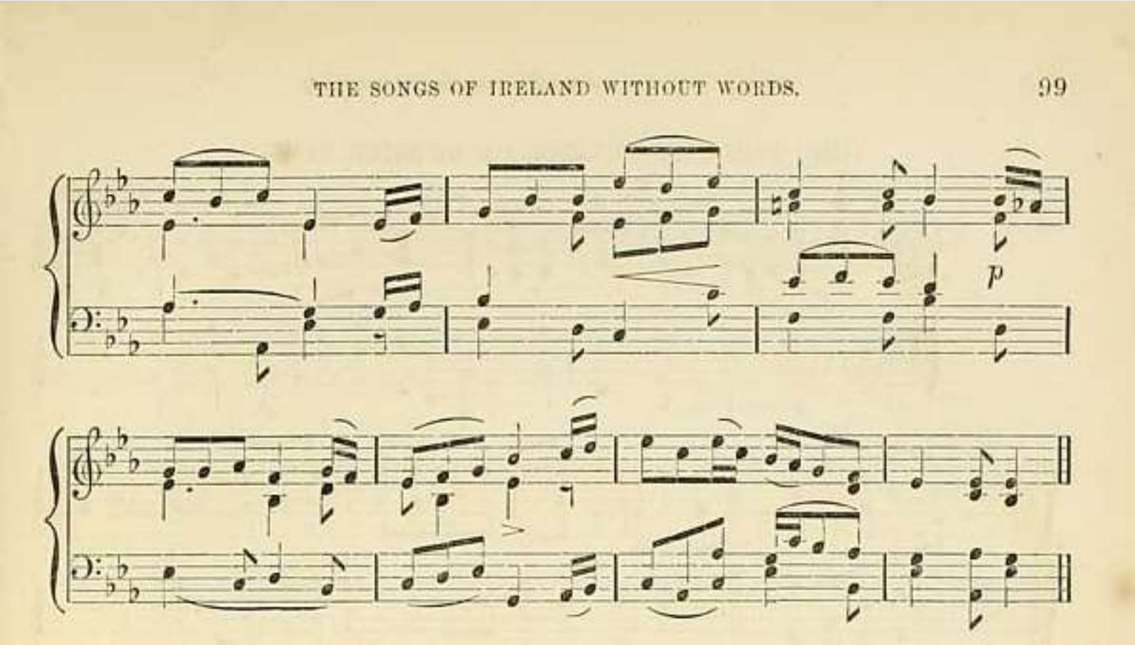

Anexo VIII– Formulário de notificação de evento adverso

Iniciais do participante: _______________ Nr. de Randomização: _________

Relato Inicial

1) Descrições do evento adverso segundo a participante da pesquisa (Preenchimento do Técnico):

____

____

____

____

Será encaminhado para avaliação médica?

( ) Sim ( ) Não

Se sim, preencha as questões 1 a 4

Nome do Responsável: ____

Assinatura: ____ **Data** _________

Iniciais do participante: _______________ Nr. de Randomização: _________

Relato Médico

1) Descrever a avaliação médica:

____

____

____

____

____

2) Este evento está relacionado com a Pesquisa (este evento está relacionado com o produto)?

( ) Sim **(Positivo)** ( ) Não **(Negativo)** ( ) Duvidoso

Comentários (preenchimento obrigatório):

____

____

____

3) Qual a conduta a ser tomada?

____

____

____

4) O caso foi encerrado?

□ Sim

□ Não (continuar o seguimento de relato adverso preenchendo as questões 5 a 7)

5) Haverá investigação?

□ Sim

□ Não

6) Qual a hipótese diagnóstica?

____

____

____

7) Tratamento

□ Sim (se sim, descrever abaixo)

□ Não

____

____

____

Nome do Médico: ___________________________________

Assinatura:_____________________________________ Data _________

Iniciais do participante: _______________ Nr. de Randomização: _________

Relato Médico

Seguimento __

8) Descrever a avaliação médica:

____

____

____

____

9) Comentários:

____

____

____

10) Evolução do Evento Adverso:

□ Regressão total (Recuperado)

□ Melhora parcial

□ Quadro mantido

□ Piora do quadro

Data de encerramento do evento: ___________________

Nome do Médico: ______________________________________

Assinatura:__________________________________ Data ___________

Observação: Este documento deverá ser arquivado junto com o *checklist* de critérios de inclusão e exclusão do voluntário.

Anexo IX – Distribuição da frequência das avaliações do estudo

	Dados Sociodemográficos	Avaliação Dermatológica	Avaliação de Stress		Avaliação de autoestima	Escalas de bem estar subjetivo		Percepção da experiência	Experiência simbólica	Follow-u
			Objetiva	Subjetiva						
Tempo	Questionário	Avaliação médica	Cortisol salivar	ISSL e LSS	Rosenberg	Satisfação com a vida	Afeto positivo e negativo	Diário	Desenho	Adesão a autocuida
Baseline	X	X	X	X	X	X	X	X	X	
15 dias			X	X	X	X	X	X		
30 dias		X	X	X	X	X	X	X	X	
Follow-up 30 dias			X	X	X	X	X			X

Anexo X - Questionário de Informações socioeconômicas

Iniciais do participante: _______________ Nr. de Randomização: _________

Instruções. Gostaríamos de conhecer algo mais acerca de nossos participantes. Não é necessário se identificar. Todas as respostas serão tratadas no conjunto.

01. Idade: _____anos

02.Estado Civil:( ) Casada/Convivente ( )Solteira ( )Viúva ( )Separada/Divorciada

03. Tem filhos? ( ) sim ( )não

Se sim, quantos? __________

04. Você possui alguma crença espiritual: ( ) Sim ( ) Não

05. Qual a sua escolaridade? ( ) nível técnico ( ) graduação ( ) MBA ( )Mestrado Profissional ( ) Mestrado ( ) Doutorado ( )pós-doutorado

06. Quanto tempo tem de formado: _______ anos

07. Qual sua área de trabalho? ( ) Setor Aberto ( ) Setor fechado (UTI, CC)

08. Há quanto tempo atua nessa área: _____ anos

09. Qual seu horário de trabalho: ( ) Somente manhã ( ) Somente tarde ( ) período integral

10. Qual sua renda mensal aproximada:

( ) 1 a 3 salários mínimos

( ) 4 a 10 salários mínimos

( ) 10 a 20 salários mínimos

( ) acima de 20 salários mínimos

11. Você está passando por dificuldades financeiras ( ) Sim ( ) Não

12. Você está passando por alguma dificuldade física e/ou emocional? ( ) Sim ( ) Não

13. Assinale quantas das práticas a seguir você realiza com frequência:

( ) alimentação saudável, equilibrada e em horários regulares

( ) atividade física regular

( ) oito horas de sono diárias

( ) acupuntura

( ) massagem

( ) atividade espiritual

( ) meditação

( ) lazer

14. Você exerce alguma outra atividade que você considera um autocuidado? ( ) sim ( ) não

Se sim. Qual? ____

Com que frequência você realiza:

( ) não realiza toda semana ( ) de 1 uma a quatro vezes por semana ( ) acima de quatro vezes por semana

ANEXO XI – Inventário de Sintomas de Stress para adultos de Lipp - ISSL

Assinale os sintomas que tem experimentado nas **ÚLTIMAS 24 HORAS:**

( ) Mãos e/ou pés frios
( ) Boca Seca
( ) Nó ou dor no estômago
( ) Aumento de sudorese (muito suor)
( ) Tensão muscular (dores nas costas, pescoço, ombros)
( ) Aperto na mandíbula/ranger de dentes, ou roer unhas ou ponta de caneta
( ) Diarréia passageira
( ) Insônia, dificuldade de dormir
( ) Taquicardia (batimentos acelerados do coração)
( ) Respiração ofegante, entrecortada
( ) Hipertensão súbita e passageira (pressão alta súbita e passageira)
( ) Mudança de apetite (comer bastante ou Ter falta de apetite)
( ) Aumento súbito de motivação
( ) Entusiasmo súbito
( ) Vontade súbita de iniciar novos projetos

Assinale os sintomas que tem experimentado na **ÚLTIMA SEMANA**:

( ) Problemas com a memória, esquecimentos
( ) Mal-estar generalizado, sem causa específica
( ) Formigamento nas extremidades (pés ou mãos)
( ) Sensação de desgaste físico constante
( ) Mudança de apetite
( ) Aparecimento de problemas dermatológicos (pele)
( ) Hipertensão arterial (pressão alta)
( ) Cansaço Constante
( ) Aparecimento de gastrite prolongada (queimação no estômago, azia)
( ) Tontura, sensação de estar flutuando
( ) Sensibilidade emotiva excessiva, emociona-se por qualquer coisa
( ) Dúvidas quanto a si próprio
( ) Pensamento constante sobre um só assunto
( ) Irritabilidade excessiva
( ) Diminuição da libido (desejo sexual diminuído)

Assinale os sintomas que tem experimentado nos **ÚLTIMO MÊS:**

( ) Diarréias freqüentes
( ) Dificuldades Sexuais
( ) Formigamento nas extremidades (mãos e pés)
( ) Insônia
( ) Tiques nervosos
( ) Hipertensão arterial confirmada
( ) Problemas dermatológicos prolongados (pele)
( ) Mudança extrema de apetite
( ) Taquicardia (batimento acelerado do coração)
( ) Tontura freqüente
( ) Úlcera
( ) Impossibilidade de Trabalhar
( ) Pesadelos
( ) Sensação de incompetência em todas as áreas
( ) Vontade de fugir de tudo
( ) Apatia, vontade de nada fazer, depressão ou raiva prolongada
( ) Cansaço excessivo
( ) Pensamento constante sobre um mesmo assunto
( ) Irritabilidade sem causa aparente
( ) Angústia ou ansiedade diária

( ) Hipersensibilidade emotiva
( ) Perda do senso de humor

Iniciais do participante: _______________ Nr. de Randomização: _________

ANEXO XII – Lista de Sintomas de Stress para adulto (LSS)

Nome:
____

Por favor, indique com qual frequência você apresenta os sintomas descritos abaixo.
Assinale o número correspondente a sua resposta, obedecendo o seguinte critério:

0 = nunca
1= poucas vezes
2 = frequentemente
3 = sempre

No.	Sintoma:	Concordo:			
1.	Sinto a respiração ofegante.	0	1	2	3
2.	Qualquer coisa me apavora.	0	1	2	3
3.	Tenho taquicardia.	0	1	2	3
4.	Tenho a sensação que vou desmaiar.	0	1	2	3
5.	No final de um dia de trabalho, sinto-me desgastado.	0	1	2	3
6.	Sinto falta de apetite.	0	1	2	3
7.	Como demais.	0	1	2	3
8.	Rôo as unhas.	0	1	2	3
9.	Tenho pensamentos que provocam ansiedade.	0	1	2	3
10.	Sinto-me alienado.	0	1	2	3
11.	Ranjo os dentes.	0	1	2	3
12.	Aperto as mandíbulas.	0	1	2	3
13.	Quando me levanto de manhã já estou cansado.	0	1	2	3
14.	Tenho medo.	0	1	2	3
15.	Tenho desânimo.	0	1	2	3
16.	Fico esgotado (a) emocionalmente.	0	1	2	3
17.	Sinto angústia.	0	1	2	3
18.	Noto que minhas forças estão no fim.	0	1	2	3
19.	Minha pressão é alta.	0	1	2	3
20.	Apresento distúrbio gastrintestinais.	0	1	2	3
21.	Tenho cansaço.	0	1	2	3
22.	Costumo faltar ao meu trabalho.	0	1	2	3
23.	Sinto dores nas costas.	0	1	2	3
24.	Tenho insônia.	0	1	2	3
25.	Sinto raiva.	0	1	2	3
26.	Qualquer coisa me irrita.	0	1	2	3
27.	Sinto náuseas.	0	1	2	3
28.	Fico afônico (a).	0	1	2	3
29.	Não tenho vontade de fazer as coisas.	0	1	2	3
30.	Tenho dificuldades de relacionamento.	0	1	2	3
31.	Ouço zumbidos no ouvido.	0	1	2	3
32.	Fumo demais.	0	1	2	3
33.	Sinto sobrecarga de trabalho.	0	1	2	3
34.	Sinto depressão.	0	1	2	3
35.	Esqueço-me das coisas.	0	1	2	3

36.	Sinto o corpo coberto de suor frio.	0	1	2	3
37.	Sinto os olhos lacrimejando e a visão embaçada.	0	1	2	3
38.	Sinto exaustão física.	0	1	2	3
39.	Tenho sono exagerado.	0	1	2	3
40.	Sinto insegurança.	0	1	2	3
41.	Sinto pressão no peito	0	1	2	3
42.	Sinto preocupações.	0	1	2	3
43.	Sinto insatisfação com meu trabalho.	0	1	2	3
44.	Tenho dor de cabeça.	0	1	2	3
45.	Tenho as mãos e/ou os pés frios.	0	1	2	3
46.	Tenho a boca seca.	0	1	2	3
47.	Sinto que meu desempenho no trabalho está limitado.	0	1	2	3
48.	Tenho pesadelos.	0	1	2	3
49.	Tenho um nó no estômago.	0	1	2	3
50.	Tenho dúvidas sobre mim mesmo(a).	0	1	2	3
51.	Sofro de enxaqueca.	0	1	2	3
52.	Meu apetite oscila muito.	0	1	2	3
53.	Tem dias que, de repente, tenho diarréia.	0	1	2	3
54.	Minha vida sexual está difícil.	0	1	2	3
55.	Meus músculos estão sempre tensos.	0	1	2	3
56.	Tenho vontade de abandonar tudo que estou fazendo.	0	1	2	3
57.	Tenho discutido frequentemente com meus amigos e familiares.	0	1	2	3
58.	Evito festas, jogos e reuniões sociais.	0	1	2	3
59.	Tenho vontade de ficar sozinho.	0	1	2	3

Pontuação:

1 (poucas vezes) x ______ questões = ______ pontos
2 (frequentemente) x ______ questões = ______ pontos
3 (sempre) x ______ questões = ______ pontos

TOTAL = ______pontos

ANEXO XIII -Escala de autoestima de Rosenberg

Iniciais do participante: _______________ Nr. de Randomização: _________

Escala de Autoestima de Rosenberg (1965) versão validada por Dini (2001)

a) Concordo plenamente	b) Concordo	c) Discordo	d) Discordo plenamente
1	2	3	4

1) De uma forma geral (apesar de tudo) estou satisfeito comigo mesmo (a).

1	2	3	4

2) Às vezes, eu acho que não sirvo para nada (desqualificado ou inferior em relação aos outros).

1	2	3	4

3) Eu sinto que eu tenho um tanto (um número) de boas qualidades.

1	2	3	4

4) Eu sou capaz de fazer coisas tão bem quanto a maioria das outras pessoas (desde que ensinadas)

1	2	3	4

5) Não sinto satisfação nas coisas que realizei. Eu sinto que não tenho muito do que me orgulhar.

1	2	3	4

6) Às vezes, eu realmente me sinto inútil (incapaz de fazer as coisas).

1	2	3	4

7) Eu sinto que sou uma pessoa de valor, pelo menos num plano igual (num mesmo nível) às outras pessoas.

1	2	3	4

8) Eu gostaria de ter mais respeito por mim mesmo (a). (Dar-me mais valor).

1	2	3	4

9) Quase sempre estou inclinado (a) a achar que sou um (a) fracasso (a).

1	2	3	4

10) Eu tenho uma atitude positiva (pensamentos, atos e sentimentos positivos) em relação a mim mesmo (a).

1	2	3	4

ANEXO XIV - Escala de Satisfação com a Vida

Iniciais do participante: _______________ Nr. de Randomização: _________

Instruções: Abaixo você encontrará cinco afirmações com as quais pode ou não concordar. Usando a escala de resposta a seguir, que vai de 1 a 7, indique o quanto concorda ou discorda com cada uma; escreva um número no espaço ao lado da afirmação, segundo sua opinião. Por favor, seja o mais sincero possível nas suas respostas.

7 = Concordo totalmente

6 = Concordo

5 = Concordo ligeiramente

4 = Nem concordo nem discordo

3 = Discordo ligeiramente

2 = Discordo

1 = Discordo totalmente

1._____Na maioria dos aspectos, minha vida é próxima ao meu ideal.

2._____As condições da minha vida são excelentes.

3._____Estou satisfeito(a) com minha vida.

4._____Dentro do possível, tenho conseguido as coisas importantes que quero da vida.

5._____Se pudesse viver uma segunda vez, não mudaria quase nada na minha vida.

ANEXO XV - Escala de afetos positivos e negativos

Iniciais do participante: _______________ Nr. de Randomização: _________

QUESTIONÁRIO DE AFETOS

INSTRUÇÕES. **A seguir você encontrará uma lista com dez** estados emocionais. **Para cada um deles, pedimos-lhe que indique** o quanto você o tem experimentado *ultimamente*. **Faça isso escrevendo um número no espaço ao lado de cada emoção/adjetivo, segundo a escala de resposta abaixo, de acordo com a sua opinião. Por favor, seja o mais sincero e honesto possível nas suas respostas.**

1	2	3	4	5	6	7
Nada	Muito Pouco	Pouco	Mais ou menos	Bastante	Muito	Extremamente

01. ______Feliz
02. ______Deprimido
03. ______Satisfeito
04. ______Frustrado
05. ______Raivoso
06. ______ Divertido
07. ______ Preocupado
08. ______ Otimista
09. ______ Infeliz
10. ______ Alegre

ANEXO XVI – Diário (Grupos 2 e 3)1 ° Dia Data:___ /___ /___ Horário:________

Iniciais do participante: _______________ Nr. de Randomização: _________

Data da última menstruação:___ /___ /___

Quantos minutos você gastou em média para o seu autocuidado: _________

Local em que você realizou o seu autocuidado:
( )Quarto ( )Banheiro ( )Outro: _________________________________

Que sensações e/ou emoções você experimentou nesta experiência de hoje? Descreva ou nos fale um pouco sobre elas.

____
____
____
____
____
____

2 ° Dia Data:___ /___ /___ Horário:________

Quantos minutos você gastou em média para o seu autocuidado: _________

Local em que você realizou o seu autocuidado:
( )Quarto ( )Banheiro ( )Outro: _________________________________

Que sensações e/ou emoções você experimentou nesta experiência de hoje? Descreva ou nos fale um pouco sobre elas.

____
____
____
____
____
____

Iniciais do participante: ________________ número de randomização: _____

3 ° Dia Data:___ /___ /___ Horário:________

Quantos minutos você gastou em média para o seu autocuidado: _________

Local em que você realizou o seu autocuidado:
( )Quarto ( )Banheiro ( )Outro: _________________________________

Que sensações e/ou emoções você experimentou nesta experiência de hoje? Descreva ou nos fale um pouco sobre elas.

____
____
____
____
____
____

4° Dia Data:___ /___ /___ Horário:________

Quantos minutos você gastou em média para o seu autocuidado: _________

Local em que você realizou o seu autocuidado:
( )Quarto ( )Banheiro ( )Outro: _________________________________

Que sensações e/ou emoções você experimentou nesta experiência de hoje? Descreva ou nos fale um pouco sobre elas.

____
____
____
____
____
____

5° Dia Data:___ /___ /___ Horário:________

Quantos minutos você gastou em média para o seu autocuidado: _________

Local em que você realizou o seu autocuidado:
( )Quarto ( )Banheiro ( )Outro: _________________________________

Que sensações e/ou emoções você experimentou nesta experiência de hoje? Descreva ou nos fale um pouco sobre elas.

____
____
____
____
____
____

Iniciais do participante: ________________ número de randomização: _____

6° Dia Data:___ /___ /___ Horário:________

Quantos minutos você gastou em média para o seu autocuidado: _________

Local em que você realizou o seu autocuidado:
( )Quarto ( )Banheiro ( )Outro: _________________________________

Que sensações e/ou emoções você experimentou nesta experiência de hoje? Descreva ou nos fale um pouco sobre elas.

____
____
____
____
____
____

7° Dia Data:___ /___ /___ Horário:________

Quantos minutos você gastou em média para o seu autocuidado: _________

Local em que você realizou o seu autocuidado:
( )Quarto ( )Banheiro ( )Outro: _________________________________

Que sensações e/ou emoções você experimentou nesta experiência de hoje? Descreva ou nos fale um pouco sobre elas.

____
____
____
____
____
____

8° Dia Data:___ /___ /___ Horário:________

Quantos minutos você gastou em média para o seu autocuidado: _________

Local em que você realizou o seu autocuidado:
( )Quarto ( )Banheiro ( )Outro: _________________________________

Que sensações e/ou emoções você experimentou nesta experiência de hoje? Descreva ou nos fale um pouco sobre elas.

____
____
____
____
____
____

Iniciais do participante: ________________ número de randomização: _____

9° Dia Data:___ /___ /___ Horário:________

Quantos minutos você gastou em média para o seu autocuidado: _________

Local em que você realizou o seu autocuidado:
( )Quarto ( )Banheiro ( )Outro: _________________________________

Que sensações e/ou emoções você experimentou nesta experiência de hoje? Descreva ou nos fale um pouco sobre elas.

____
____
____
____
____
____

10° Dia Data:___ /___ /___ Horário:________

Quantos minutos você gastou em média para o seu autocuidado: _________

Local em que você realizou o seu autocuidado:
( )Quarto ( )Banheiro ( )Outro: _________________________________

Que sensações e/ou emoções você experimentou nesta experiência de hoje? Descreva ou nos fale um pouco sobre elas.

____
____
____
____
____
____

11° Dia Data:___ /___ /___ Horário:________

Quantos minutos você gastou em média para o seu autocuidado: _________

Local em que você realizou o seu autocuidado:
( )Quarto ( )Banheiro ( )Outro: _________________________________

Que sensações e/ou emoções você experimentou nesta experiência de hoje? Descreva ou nos fale um pouco sobre elas.

____
____
____
____
____
____

Iniciais do participante: ________________ número de randomização: _____

12° Dia Data:___ /___ /___ Horário:________

Quantos minutos você gastou em média para o seu autocuidado: _________

Local em que você realizou o seu autocuidado:
( )Quarto ( )Banheiro ( )Outro: _________________________________

Que sensações e/ou emoções você experimentou nesta experiência de hoje? Descreva ou nos fale um pouco sobre elas.

____
____
____
____
____
____

13° Dia Data:___ /___ /___ Horário:________

Quantos minutos você gastou em média para o seu autocuidado: _________

Local em que você realizou o seu autocuidado:
( )Quarto ( )Banheiro ( )Outro: _________________________________

Que sensações e/ou emoções você experimentou nesta experiência de hoje? Descreva ou nos fale um pouco sobre elas.

____
____
____
____
____
____

14° Dia Data:___ /___ /___ Horário:________

Quantos minutos você gastou em média para o seu autocuidado: _________

Local em que você realizou o seu autocuidado:
( )Quarto ( )Banheiro ( )Outro: _________________________________

Que sensações e/ou emoções você experimentou nesta experiência de hoje? Descreva ou nos fale um pouco sobre elas.

____
____
____
____
____
____
____

*Lembre-se que você tem uma data agendada conosco para sua avaliação de 15 dias.

Iniciais do participante: ________________ número de randomização: _____

15° Dia Data:___ /___ /___ Horário:________

Qual a data da sua última menstruação? ___/___/____

Quantos minutos você gastou em média para o seu autocuidado: _________

Local em que você realizou o seu autocuidado:
( )Quarto ( )Banheiro ( )Outro: _________________________________

Que sensações e/ou emoções você experimentou nesta experiência de hoje? Descreva ou nos fale um pouco sobre elas.
____
____
____
____
____
____

Iniciais do participante: _______________ número de randomização: _____

16° Dia Data:___ /___ /___ Horário:________

Quantos minutos você gastou em média para o seu autocuidado: _________

Local em que você realizou o seu autocuidado:
( )Quarto ( )Banheiro ( )Outro: _________________________________

Que sensações e/ou emoções você experimentou nesta experiência de hoje? Descreva ou nos fale um pouco sobre elas.

____
____
____
____
____
____

17° Dia Data:___ /___ /___ Horário:________

Quantos minutos você gastou em média para o seu autocuidado: _________

Local em que você realizou o seu autocuidado:
( )Quarto ( )Banheiro ( )Outro: _________________________________

Que sensações e/ou emoções você experimentou nesta experiência de hoje? Descreva ou nos fale um pouco sobre elas.

____
____
____
____
____

18° Dia Data:___ /___ /___ Horário:________

Quantos minutos você gastou em média para o seu autocuidado: _________

Local em que você realizou o seu autocuidado:
( )Quarto ( )Banheiro ( )Outro: _________________________________

Que sensações e/ou emoções você experimentou nesta experiência de hoje? Descreva ou nos fale um pouco sobre elas.

____
____
____
____
____
____

Iniciais do participante: ________________ número de randomização: _____

19° Dia Data:___ /___ /___ Horário:________

Quantos minutos você gastou em média para o seu autocuidado: _________

Local em que você realizou o seu autocuidado:
( )Quarto ( )Banheiro ( )Outro: _________________________________

Que sensações e/ou emoções você experimentou nesta experiência de hoje? Descreva ou nos fale um pouco sobre elas.

____
____
____
____
____
____

20° Dia Data:___ /___ /___ Horário:________

Quantos minutos você gastou em média para o seu autocuidado: _________

Local em que você realizou o seu autocuidado:
( )Quarto ( )Banheiro ( )Outro: _________________________________

Que sensações e/ou emoções você experimentou nesta experiência de hoje? Descreva ou nos fale um pouco sobre elas.

____
____
____
____
____
____

21° Dia Data:___ /___ /___ Horário:________

Quantos minutos você gastou em média para o seu autocuidado: _________

Local em que você realizou o seu autocuidado:
( )Quarto ( )Banheiro ( )Outro: _________________________________

Que sensações e/ou emoções você experimentou nesta experiência de hoje? Descreva ou nos fale um pouco sobre elas.

____
____
____
____
____
____

Iniciais do participante: ________________ número de randomização: _____

22° Dia Data:___ /___ /___ Horário:________

Quantos minutos você gastou em média para o seu autocuidado: _________

Local em que você realizou o seu autocuidado:
( )Quarto ( )Banheiro ( )Outro: _________________________________

Que sensações e/ou emoções você experimentou nesta experiência de hoje? Descreva ou nos fale um pouco sobre elas.

____
____
____
____
____
____

23° Dia Data:___ /___ /___ Horário:________

Quantos minutos você gastou em média para o seu autocuidado: _________

Local em que você realizou o seu autocuidado:
( )Quarto ( )Banheiro ( )Outro: _________________________________

Que sensações e/ou emoções você experimentou nesta experiência de hoje? Descreva ou nos fale um pouco sobre elas.

____
____
____
____
____
____

24° Dia Data:___ /___ /___ Horário:________

Quantos minutos você gastou em média para o seu autocuidado: _________

Local em que você realizou o seu autocuidado:
( )Quarto ( )Banheiro ( )Outro: _________________________________

Que sensações e/ou emoções você experimentou nesta experiência de hoje? Descreva ou nos fale um pouco sobre elas.

____
____
____
____
____
____

Iniciais do participante: ________________ número de randomização: _____

25° Dia Data:___ /___ /___ Horário:________

Quantos minutos você gastou em média para o seu autocuidado: _________

Local em que você realizou o seu autocuidado:
( )Quarto ( )Banheiro ( )Outro: _________________________________

Que sensações e/ou emoções você experimentou nesta experiência de hoje? Descreva ou nos fale um pouco sobre elas.

____
____
____
____
____
____

26° Dia Data:___ /___ /___ Horário:________

Quantos minutos você gastou em média para o seu autocuidado: _________

Local em que você realizou o seu autocuidado:
( )Quarto ( )Banheiro ( )Outro: _________________________________

Que sensações e/ou emoções você experimentou nesta experiência de hoje? Descreva ou nos fale um pouco sobre elas.

____
____
____
____
____
____

27° Dia Data:___ /___ /___ Horário:________

Quantos minutos você gastou em média para o seu autocuidado: _________

Local em que você realizou o seu autocuidado:
( )Quarto ( )Banheiro ( )Outro: _________________________________

Que sensações e/ou emoções você experimentou nesta experiência de hoje? Descreva ou nos fale um pouco sobre elas.

____
____
____
____
____
____

Iniciais do participante: ________________ número de randomização: _____

28° Dia Data:___ /___ /___ Horário:________

Quantos minutos você gastou em média para o seu autocuidado: _________

Local em que você realizou o seu autocuidado:
( )Quarto ( )Banheiro ( )Outro: _________________________________

Que sensações e/ou emoções você experimentou nesta experiência de hoje? Descreva ou nos fale um pouco sobre elas.

____
____
____
____
____
____

29° Dia Data:___ /___ /___ Horário:________

Quantos minutos você gastou em média para o seu autocuidado: _________

Local em que você realizou o seu autocuidado:
( )Quarto ( )Banheiro ( )Outro: _________________________________

Que sensações e/ou emoções você experimentou nesta experiência de hoje? Descreva ou nos fale um pouco sobre elas.

____
____
____
____
____
____

30° Dia Data:___ /___ /___ Horário:________

Qual a data da sua última menstruação? ___/___/____

Quantos minutos você gastou em média para o seu autocuidado: _________

Local em que você realizou o seu autocuidado:
( )Quarto ( )Banheiro ( )Outro: _________________________________

Que sensações e/ou emoções você experimentou nesta experiência de hoje? Descreva ou nos fale um pouco sobre elas.

____
____
____
____
____
____

Iniciais do participante: ________________ número de randomização: _____

Obrigado por ter participado destes trinta dias de pesquisa.
Lembre-se que você tem uma data agendada conosco para sua avaliação de 30 dias.
Não se esqueça de trazer o(s) frasco(s) independente do volume que ele(s) contenha (m)

Como foi para você participar desta experiência de autocuidado?

ANEXO XVII – Diário (Grupo 4)

Iniciais do participante: ________________ número de randomização: _____

1 ° Dia Data:___ /___ /___ Horário:________

Data da última menstruação:___ /___ /___

Quantos minutos você gastou em média para o seu autocuidado: _________

Local em que você realizou o seu autocuidado:
( )Quarto ( )Banheiro ( )Outro: _________________________________

Que sensações e/ou emoções você experimentou nesta experiência de hoje? Descreva ou nos fale um pouco sobre elas.

____
____
____
____
____
____

2 ° Dia Data:___ /___ /___ Horário:________

Quantos minutos você gastou em média para o seu autocuidado: _________

Local em que você realizou o seu autocuidado:
( )Quarto ( )Banheiro ( )Outro: _________________________________

Que sensações e/ou emoções você experimentou nesta experiência de hoje? Descreva ou nos fale um pouco sobre elas.

____
____
____
____
____
____

Iniciais do participante: ________________ número de randomização: _____

3 ° Dia Data:___ /___ /___ Horário:________

Quantos minutos você gastou em média para o seu autocuidado: _________

Local em que você realizou o seu autocuidado:
( )Quarto ( )Banheiro ( )Outro: _________________________________

Que sensações e/ou emoções você experimentou nesta experiência de hoje? Descreva ou nos fale um pouco sobre elas.

____
____
____
____
____
____

4° Dia Data:___ /___ /___ Horário:________

Quantos minutos você gastou em média para o seu autocuidado: _________

Local em que você realizou o seu autocuidado:
( )Quarto ( )Banheiro ( )Outro: _________________________________

Que sensações e/ou emoções você experimentou nesta experiência de hoje? Descreva ou nos fale um pouco sobre elas.

____
____
____
____
____
____

5° Dia Data:___ /___ /___ Horário:________

Quantos minutos você gastou em média para o seu autocuidado: _________

Local em que você realizou o seu autocuidado:
( )Quarto ( )Banheiro ( )Outro: _________________________________

Que sensações e/ou emoções você experimentou nesta experiência de hoje? Descreva ou nos fale um pouco sobre elas.

____
____
____
____
____
____

Iniciais do participante: ________________ número de randomização: _____

6° Dia Data:___ /___ /___ Horário:________

Quantos minutos você gastou em média para o seu autocuidado: _________

Local em que você realizou o seu autocuidado:
( )Quarto ( )Banheiro ( )Outro: _________________________________

Que sensações e/ou emoções você experimentou nesta experiência de hoje? Descreva ou nos fale um pouco sobre elas.

____
____
____
____
____
____

7° Dia Data:___ /___ /___ Horário:________

Quantos minutos você gastou em média para o seu autocuidado: _________

Local em que você realizou o seu autocuidado:
( )Quarto ( )Banheiro ( )Outro: _________________________________

Que sensações e/ou emoções você experimentou nesta experiência de hoje? Descreva ou nos fale um pouco sobre elas.

____
____
____
____
____
____

8° Dia Data:___ /___ /___ Horário:________

Quantos minutos você gastou em média para o seu autocuidado: _________

Local em que você realizou o seu autocuidado:
( )Quarto ( )Banheiro ( )Outro: _________________________________

Que sensações e/ou emoções você experimentou nesta experiência de hoje? Descreva ou nos fale um pouco sobre elas.

____
____
____
____
____
____

Iniciais do participante: ________________ número de randomização: _____

9° Dia Data:___ /___ /___ Horário:________

Quantos minutos você gastou em média para o seu autocuidado: _________

Local em que você realizou o seu autocuidado:
( )Quarto ( )Banheiro ( )Outro: _________________________________

Que sensações e/ou emoções você experimentou nesta experiência de hoje? Descreva ou nos fale um pouco sobre elas.

____
____
____
____
____
____

10° Dia Data:___ /___ /___ Horário:________

Quantos minutos você gastou em média para o seu autocuidado: _________

Local em que você realizou o seu autocuidado:
( )Quarto ( )Banheiro ( )Outro: _________________________________

Que sensações e/ou emoções você experimentou nesta experiência de hoje? Descreva ou nos fale um pouco sobre elas.

____
____
____
____
____
____

11° Dia Data:___ /___ /___ Horário:________

Quantos minutos você gastou em média para o seu autocuidado: _________

Local em que você realizou o seu autocuidado:
( )Quarto ( )Banheiro ( )Outro: _________________________________

Que sensações e/ou emoções você experimentou nesta experiência de hoje? Descreva ou nos fale um pouco sobre elas.

____
____
____
____
____
____

Iniciais do participante: ________________ número de randomização: _____

12° Dia Data:___ /___ /___ Horário:________

Quantos minutos você gastou em média para o seu autocuidado: _________

Local em que você realizou o seu autocuidado:
( )Quarto ( )Banheiro ( )Outro: _________________________________

Que sensações e/ou emoções você experimentou nesta experiência de hoje? Descreva ou nos fale um pouco sobre elas.

____
____
____
____
____
____

13° Dia Data:___ /___ /___ Horário:________

Quantos minutos você gastou em média para o seu autocuidado: _________

Local em que você realizou o seu autocuidado:
( )Quarto ( )Banheiro ( )Outro: _________________________________

Que sensações e/ou emoções você experimentou nesta experiência de hoje? Descreva ou nos fale um pouco sobre elas.

____
____
____
____
____
____

14° Dia Data:___ /___ /___ Horário:________

Quantos minutos você gastou em média para o seu autocuidado: _________

Local em que você realizou o seu autocuidado:
( )Quarto ( )Banheiro ( )Outro: _________________________________

Que sensações e/ou emoções você experimentou nesta experiência de hoje? Descreva ou nos fale um pouco sobre elas.

____
____
____
____
____

*Lembre-se que você tem uma data agendada conosco para sua avaliação de 15 dias.

Iniciais do participante: ________________ número de randomização: _____

15° Dia Data:___ /___ /___ Horário:________

Qual a data da sua última menstruação? ___/___/____

Quantos minutos você gastou em média para o seu autocuidado: _________

Local em que você realizou o seu autocuidado:
( )Quarto ( )Banheiro ( )Outro: _________________________________

Que sensações e/ou emoções você experimentou nesta experiência de hoje? Descreva ou nos fale um pouco sobre elas.

____
____
____
____
____
____

16° Dia Data:___ /___ /___ Horário:________

Quantos minutos você gastou em média para o seu autocuidado: _________

Local em que você realizou o seu autocuidado:
( )Quarto ( )Banheiro ( )Outro: _________________________________

Que sensações e/ou emoções você experimentou nesta experiência de hoje? Descreva ou nos fale um pouco sobre elas.

____
____
____
____
____
____

17° Dia Data:___ /___ /___ Horário:________

Quantos minutos você gastou em média para o seu autocuidado: _________

Local em que você realizou o seu autocuidado:
( )Quarto ( )Banheiro ( )Outro: _________________________________

Que sensações e/ou emoções você experimentou nesta experiência de hoje? Descreva ou nos fale um pouco sobre elas.

____
____
____
____
____

Iniciais do participante: ________________ número de randomização: _____

18° Dia Data:___ /___ /___ Horário:________

Quantos minutos você gastou em média para o seu autocuidado: _________

Local em que você realizou o seu autocuidado:
( )Quarto ( )Banheiro ( )Outro: _________________________________

Que sensações e/ou emoções você experimentou nesta experiência de hoje? Descreva ou nos fale um pouco sobre elas.

____
____
____
____
____
____

19° Dia Data:___ /___ /___ Horário:________

Quantos minutos você gastou em média para o seu autocuidado: _________

Local em que você realizou o seu autocuidado:
( )Quarto ( )Banheiro ( )Outro: _________________________________

Que sensações e/ou emoções você experimentou nesta experiência de hoje? Descreva ou nos fale um pouco sobre elas.

____
____
____
____
____
____

20° Dia Data:___ /___ /___ Horário:________

Quantos minutos você gastou em média para o seu autocuidado: _________

Local em que você realizou o seu autocuidado:
( )Quarto ( )Banheiro ( )Outro: _________________________________

Que sensações e/ou emoções você experimentou nesta experiência de hoje? Descreva ou nos fale um pouco sobre elas.

____
____
____
____
____
____

Iniciais do participante: ________________ número de randomização: _____

21° Dia Data:___ /___ /___ Horário:________

Quantos minutos você gastou em média para o seu autocuidado: _________

Local em que você realizou o seu autocuidado:
( )Quarto ( )Banheiro ( )Outro: _________________________________

Que sensações e/ou emoções você experimentou nesta experiência de hoje? Descreva ou nos fale um pouco sobre elas.

____
____
____
____
____
____

22° Dia Data:___ /___ /___ Horário:________

Quantos minutos você gastou em média para o seu autocuidado: _________

Local em que você realizou o seu autocuidado:
( )Quarto ( )Banheiro ( )Outro: _________________________________

Que sensações e/ou emoções você experimentou nesta experiência de hoje? Descreva ou nos fale um pouco sobre elas.

____
____
____
____
____
____

23° Dia Data:___ /___ /___ Horário:________

Quantos minutos você gastou em média para o seu autocuidado: _________

Local em que você realizou o seu autocuidado:
( )Quarto ( )Banheiro ( )Outro: _________________________________

Que sensações e/ou emoções você experimentou nesta experiência de hoje? Descreva ou nos fale um pouco sobre elas.

____
____
____
____
____
____

Iniciais do participante: ________________ número de randomização: _____

24° Dia Data:___ /___ /___ Horário:________

Quantos minutos você gastou em média para o seu autocuidado: _________

Local em que você realizou o seu autocuidado:
( )Quarto ( )Banheiro ( )Outro: _________________________________

Que sensações e/ou emoções você experimentou nesta experiência de hoje? Descreva ou nos fale um pouco sobre elas.

____
____
____
____
____
____

25° Dia Data:___ /___ /___ Horário:________

Quantos minutos você gastou em média para o seu autocuidado: _________

Local em que você realizou o seu autocuidado:
( )Quarto ( )Banheiro ( )Outro: _________________________________

Que sensações e/ou emoções você experimentou nesta experiência de hoje? Descreva ou nos fale um pouco sobre elas.

____
____
____
____
____
____

26° Dia Data:___ /___ /___ Horário:________

Quantos minutos você gastou em média para o seu autocuidado: _________

Local em que você realizou o seu autocuidado:
( )Quarto ( )Banheiro ( )Outro: _________________________________

Que sensações e/ou emoções você experimentou nesta experiência de hoje? Descreva ou nos fale um pouco sobre elas.

____
____
____
____
____
____

Iniciais do participante: ________________ número de randomização: _____

27° Dia Data:___ /___ /___ Horário:________

Quantos minutos você gastou em média para o seu autocuidado: _________

Local em que você realizou o seu autocuidado:
( )Quarto ( )Banheiro ( )Outro: _________________________________

Que sensações e/ou emoções você experimentou nesta experiência de hoje? Descreva ou nos fale um pouco sobre elas.

____
____
____
____
____
____

28° Dia Data:___ /___ /___ Horário:________

Quantos minutos você gastou em média para o seu autocuidado: _________

Local em que você realizou o seu autocuidado:
( )Quarto ( )Banheiro ( )Outro: _________________________________

Que sensações e/ou emoções você experimentou nesta experiência de hoje? Descreva ou nos fale um pouco sobre elas.

____
____
____
____
____
____

29° Dia Data:___ /___ /___ Horário:________

Quantos minutos você gastou em média para o seu autocuidado: _________

Local em que você realizou o seu autocuidado:
( )Quarto ( )Banheiro ( )Outro: _________________________________

Que sensações e/ou emoções você experimentou nesta experiência de hoje? Descreva ou nos fale um pouco sobre elas.

____
____
____
____
____
____

Iniciais do participante: ________________ número de randomização: _____

30° Dia Data:___ /___ /___ Horário:________

Qual a data da sua última menstruação? ___/___/____

Quantos minutos você gastou em média para o seu autocuidado: _________

Local em que você realizou o seu autocuidado:
( )Quarto ( )Banheiro ( )Outro: _________________________________

Que sensações e/ou emoções você experimentou nesta experiência de hoje? Descreva ou nos fale um pouco sobre elas.

Obrigado por ter participado destes trinta dias de pesquisa.
Lembre-se que você tem uma data agendada conosco para sua avaliação de 30 dias.
Não se esqueça de trazer o(s) frasco(s) independente do volume que ele(s) contenha (m).
Não se esqueça também de trazer o tablet para devolvê-lo à equipe do estudo.

Como foi para você participar desta experiência de autocuidado?

Iniciais do participante: ________________ número de randomização: _____

Durante os trinta dias de utilização do produto você verificou algum sinal clínico?

Sim ( )
Não ( )
Se sim, qual? ____

Entrou em contato com o responsável pela condução do estudo?

Sim ( )
Não ( )
Porque?____
____

Iniciais do participante: _______________ Nr. de Randomização: _________

Quanto ao material audiovisual onde zero representa "não gostei nada" e dez, "Gostei muito":

Dê uma nota de zero a dez que represente o quanto você gostou do material audiovisual,
( ) 0 ( )1 ( )2 ( )3 ( ) 4 ( )5 ( )6 ( )7 ( ) 8 ( )9 ( )10

Dê uma nota de zero a dez que represente o quanto você gostou das imagens:
( ) 0 ( )1 ( )2 ( )3 ( ) 4 ( )5 ( )6 ( )7 ( ) 8 ( )9 ( )10

Dê uma nota de zero a dez que represente o quanto você gostou da música:
( ) 0 ( )1 ( )2 ( )3 ( ) 4 ( )5 ( )6 ( )7 ( ) 8 ( )9 ( )10

Qual gênero musical você mais gosta?____

Você tem conhecimentos musicais (é músico ou toca algum instrumento musical)?
( )sim ( )não

Você assistiu o material audiovisual todos os dias? ( ) sim ( ) não

Caso não tenha assistido:
Quantos dias:______________
Porquê?
____
____
____
____
____
____
____
____
____

Anexo XVIII – Desenho

Iniciais do participante: ________________   número de randomização: _____

( )pré-estudo ( )pós-estudo

Faça um desenho que represente como você tem se sentido nos últimos 30 dias.

Anexo XIX – Follow-up

Iniciais do participante: _________________ número de randomização: _____

Você tem exercitado o autocuidado nestes últimos 30 dias? ( )sim ( )não

Se sim, como?

____
____
____
____
____
____
____
____
____
____
____
____
____
____
____

Anexo XX

Iniciais do participante: _______________ Nr. de Randomização: _________

Grupo da randomização: Grupo 1 □ Grupo 2 □ Grupo 3 □ Grupo 4 □

Termo de Consentimento Livre e Esclarecido

PESQUISA: Autocuidado mediado pelos sentidos: avaliação do impacto na autoestima, stress e bem-estar de mulheres profissionais de saúde.

Pesquisadora: Eliseth Ribeiro Leão

Prezada colaboradora,

Você é convidada a participar desta pesquisa, que tem como finalidade conhecer como as profissionais de saúde respondem a uma prática diária de autocuidado.

1. PARTICIPANTES DA PESQUISA: profissionais da saúde da Sociedade Beneficente Israelita Brasileira Albert Einstein.

2. ENVOLVIMENTO NA PESQUISA: Ao participar deste estudo você se compromete a realizar uma atividade autocuidado por um período de trinta dias. O estudo é composto por quatro grupos e ao aceitar participar você será sorteado para um deles explicados a seguir:

Grupo 1) é um grupo controle e você não realizará a atividade de autocuidado proposta neste estudo;

Grupo 2) você realizará uma atividade diária de hidratação corporal com um creme inodoro;

Grupo 3) você realizará uma atividade diária de hidratação corporal com um creme com fragrância;

Grupo 4) você realizará uma atividade diária de hidratação corporal com um creme com fragrância após (enquanto?) assistir um breve material audiovisual com cenas da natureza.

Quais procedimentos serão realizados?

Se você for sorteada para o Grupo 1 (grupo controle e que não terá intervenção)

Visita 01

Local: Hospital Albert Einstein

Após a assinatura do TCLE, você será convidada a responder aos seguintes questionários (4 a 10 minutos cada):

- Dados pessoais: nome, idade, unidade de trabalho, tempo de formado, tempo de instituição, horário de trabalho, ou seja, dados que nos permitirá caracterizar o perfil das participantes deste estudo.
- Inventário de Sintomas de Stress para adultos
- Escala de autoestima
- Escala de satisfação com a vida
- Escala de afetos positivos e negativos

Você irá fazer um desenho que represente a seguinte pergunta:

- *Como você está se sentindo tendo como referência sua última semana?*

Após isso, você receberá um pedido de exame para realização da coleta de cortisol salivar, que será realizado no laboratório clínico do Hospital Israelita Albert Einstein, sem nenhum custo. O cortisol é uma medida de stress coletada na saliva por um método não invasivo, indolor e seguro. A coleta é feita mediante a utilização de tubos plásticos com algodão específicos para a coleta de saliva e deverá ser coletado entre 8h e 9h conforme solicitação do exame.Este dispositivo permite uma coleta fácil e limpa.

Visita 02

Local: Hospital Albert Einstein

Após 30 dias da visita 1, você será convidada a responder aos seguintes questionários (4 a 10 minutos cada):

- Inventário de Sintomas de Stress para adultos
- Escala de autoestima
- Escala de satisfação com a vida
- Escala de afetos positivos e negativos

Você irá fazer um desenho que represente a seguinte pergunta:

- *Como você está se sentindo tendo como referência sua última semana?*

Após isso, você receberá um pedido de exame para realização da coleta de cortisol salivar, que será realizado no laboratório clínico do Hospital Israelita Albert Einstein, sem nenhum custo. O cortisol é uma medida de stress coletada na saliva por um método não invasivo, indolor e seguro. A coleta é feita mediante a utilização de tubos plásticos com algodão específicos para a coleta de saliva e deverá ser coletado entre 8h e 9h conforme solicitação do exame.Este dispositivo permite uma coleta fácil e limpa.

Se você for sorteado para o Grupo 2 ou 3 ou 4:

Visita 01

Local: Hospital Albert Einstein

Após a assinatura do TCLE, você será convidada a responder aos seguintes questionários (4 a 10 minutos cada):

- Check-list de critérios de inclusão e exclusão e avaliação do dermatologista;
- Dados pessoais: nome, idade, unidade de trabalho, tempo de formado, tempo de instituição, horário de trabalho, ou seja, dados que nos permitirá caracterizar o perfil das participantes deste estudo.
- Inventário de Sintomas de Stress para adultos
- Escala de autoestima
- Escala de satisfação com a vida
- Escala de afetos positivos e negativos

Você irá fazer um desenho que represente a seguinte pergunta:

- *Como você está se sentindo tendo como referência sua última semana?*

Após isso, você receberá um pedido de exame para realização da coleta de cortisol salivar, que será realizado no laboratório clínico do Hospital Israelita Albert Einstein, sem nenhum custo. O cortisol é uma medida de stress coletada na saliva por um método não invasivo, indolor e seguro. A coleta é feita mediante a utilização de tubos plásticos com algodão específicos para a coleta de saliva e deverá ser coletado entre 8h e 9h conforme solicitação do exame.Este dispositivo permite uma coleta fácil e limpa.

Você receberá também um diário, no qual você fará uma breve anotação sobre sua experiência após a realização da atividade diária de autocuidado proposta neste estudo, o qual você devolverá preenchido ao final do estudo.

Se você for sorteada para o Grupo 2 ou 3 você receberá a seguinte orientação em um folheto impresso:

Obrigada por ser voluntária em participar do estudo

Orientações importantes:

Obrigada por ser voluntária em participar do estudo

- Você está recebendo um creme hidratante corporal para uma atividade de autocuidado por um período de 30 dias. Esse produto não pode ser compartilhado com outras pessoas. Somente você poderá utilizá-lo neste período.

- Caso você esteja utilizando algum outro creme, você terá que suspendê-lo por 30 dias, ou seja, o tempo de duração do estudo.

- O frasco fornecido é suficiente para uso durante 30 dias. Porém, caso você perceba que o produto irá acabar antes desse prazo, entre em contato com a equipe do estudo para que um novo frasco seja providenciado a fim de evitar interrupções. Ao final do estudo você irá devolver o(s) frasco(s) independente da quantidade residual.

- Você irá escolher um horário durante seu dia para o momento de autocuidado. Neste momento você irá aplicar o creme hidratante corporal que estamos lhe fornecendo.

- Ao terminar de aplicar o produto você irá registrar no seu diário as seguintes informações: data, horário, tempo de duração do autocuidado localização física onde você realizou o autocuidado e as sensações e ou emoções proporcionadas pela experiência que acabou de ter e que você queira descrever.

- Caso você venha a apresentar qualquer tipo de alergia, vermelhidão da pele, coceira, ou qualquer manifestação que lhe chame a atenção, você deverá suspender a utilização do produto registrar no diário de uso em domicilio e entrar em contato com a equipe do estudo para receber as devidas orientações e ser avaliada por um médico dermatologista.

- Se por ventura, você se esquecer de usar o produto durante um dia registre esta informação no diário, bem como a razão pela qual você deixou de fazer o seu autocuidado. Mas faça o possível para cumprir essa prática por 30 dias consecutivos.

- Suas próximas avaliações serão em 15 dias, 30 dias e 60 dias, conforme as datas abaixo, no IIEP – 2º subsolo do Bloco A:

___/___/___ Horário: ______
___/___/___ Horário:______
___/___/___ Horário: ______

Nossos telefones: 11 973801412 ou 21511032.
Eliseth Leão
Pesquisadora Responsável do estudo H-SENSES

Se você for sorteada para o Grupo 4 você receberá a seguinte orientação em um folheto impresso:

Obrigada por ser voluntária em participar do estudo

Orientações importantes:

- Você está recebendo um creme hidratante corporal para uma atividade de autocuidado por um período de 30 dias. Esse produto não pode ser compartilhado com outras pessoas. Somente você poderá utilizá-la neste período.

- Caso você esteja utilizando algum outro produto, você terá que suspendê-lo por 30 dias, ou seja, o tempo de duração do estudo.

- O frasco fornecido é suficiente para uso durante 30 dias. Porém, caso você perceba que o produto irá acabar antes desse prazo, entre em contato com a equipe do estudo para que um novo frasco seja providenciado a fim de evitar interrupções. Ao final do estudo você irá devolver o(s) frasco(s) independente da quantidade residual no frasco. Você também irá devolver o tablet, que será entregue hoje, após a assinatura do recibo de entrega e compromisso de devolução.

- Você irá escolher um horário no seu dia para um momento de autocuidado. Neste momento você irá hidratar o seu corpo com o produto que estamos lhe fornecendo.

- Mas, antes de aplicar o creme hidratante corporal você irá assistir um material audiovisual, preparado especialmente para este seu momento e que tem a duração de 2 minutos e quarenta segundos. Este material está contido no tablet que lhe fornecemos, para acessá-lo basta abrir o link.

- Assista ao vídeo completo por uma vez, ao terminar a primeira exibição, inicie a aplicação do creme hidratante corporal, mantendo o vídeo em execução.

- Você irá registrar no seu diário as seguintes informações: data, horário, tempo de duração do autocuidado, localização física onde você realizou o autocuidado e as sensações e ou emoções proporcionadas pela experiência que acabou de ter e que você queira descrever.

- Caso você venha a apresentar qualquer tipo de alergia, vermelhidão da pele, coceira, ou qualquer manifestação que lhe chame a atenção, você deverá suspender a utilização do produto registrar no diário de uso em domicílio e entrar em contato com a equipe do estudo para receber as devidas orientações e ser avaliada por um médico dermatologista.

- Se por ventura, você se esquecer de usar a loção cremosa durante um dia registre isso no diário, bem como a razão pela qual você deixou de fazer o seu autocuidado. Mas faça o possível para cumprir essa prática por 30 dias consecutivos.

- Suas próximas avaliações serão em 15 dias, 30 dias e 60 dias, conforme as datas abaixo, no IIEP – 2º subsolo do Bloco A:
___/___/___ Horário: ______
___/___/___ Horário:______
___/___/___ Horário: ______

Nossos telefones: 11 973801412 ou 21511032.
Eliseth Leão
Pesquisadora Responsável do estudo H-SENSES

Visita 02

Local: Hospital Albert Einstein

Após 15 dias da primeira visita, você será convidada a responder novamente aos seguintes questionários (4 a 10 minutos cada):

- Inventário de Sintomas de Stress para adultos
- Escala de autoestima
- Escala de satisfação com a vida
- Escala de afetos positivos e negativos

Após isso, você receberá um pedido de exame para realização da coleta de cortisol salivar, que será realizado no laboratório clínico do Hospital Israelita Albert Einstein, sem nenhum custo.

Visita 03

Local: Hospital Albert Einstein

Após 15 dias da segunda visita, você será convidada a responder novamente aos seguintes questionários (4 a 10 minutos cada):

- Inventário de Sintomas de Stress para adultos
- Escala de autoestima
- Escala de satisfação com a vida
- Escala de afetos positivos e negativos

Após isso, você receberá um pedido de exame para realização da coleta de cortisol salivar, que será realizado no laboratório clínico do Hospital Israelita Albert Einstein, sem nenhum custo.

Também vamos pedir que você responda, por escrito, à seguinte pergunta: “Como foi esta experiência de autocuidado para você?”

Visita 04

Local: Hospital Albert Einstein

Após 30 dias da terceira visita, você deverá entregar o diário preenchido, bem como a amostra do produto, ainda que seja o frasco vazio.

Você será convidada a responder novamente aos seguintes questionários (4 a 10 minutos cada):

- Inventário de Sintomas de Stress para adultos

- Escala de autoestima
- Escala de satisfação com a vida
- Escala de afetos positivos e negativos
- Você será avaliada novamente por um dermatologista

Após isso, você receberá um pedido de exame para realização da coleta de cortisol salivar, que será realizado no laboratório clínico do Hospital Israelita Albert Einstein, sem nenhum custo.

Também vamos pedir que você responda, após trinta dias, por escrito, à seguinte pergunta: *Você tem exercitado algum tipo de autocuidado? Se sim, como?*

Lembramos que você tem a liberdade de se recusar a participar e pode ainda deixar de responder em qualquer momento da pesquisa, sem nenhum prejuízo. Além disso, ainda tem o direito de ficar com uma das vias do termo de consentimento. Sempre que quiser você poderá pedir mais informações sobre a pesquisa. Para isso, poderá entrar em contato com o coordenador da pesquisa, pelos meios informados mais abaixo, neste documento.

3. RISCOS E DESCONFORTOS: A participação nesta pesquisa apresenta riscos mínimos, talvez, apenas, algum constrangimento que algumas pessoas sentem quando estão fornecendo informações sobre si mesmas. O risco de alergia, ainda que o creme utilizado já seja dermatologicamente testado e comercializado no mercado, é sempre passível de ocorrer e solicitamos que interrompa imediatamente o seu uso, caso ocorra, e comunique o coordenador da pesquisa para as orientações cabíveis e encaminhamento ao Dermatologista da equipe de estudos e posterior encaminhamento ao serviço médico de instituição conveniada conforme benefício institucional da colaboradora, caso seja necessário.
Caso ocorra alguma reação inesperada, tais como: vermelhidão local, alergia, coceira, inchaço, você autoriza a equipe técnica a fotografar o local? ( )sim ( )não.

Os procedimentos utilizados nesta pesquisa seguem as normas estabelecidas pela Resolução 466/12 do Conselho Nacional de Saúde, e não oferecem risco a sua integridade psíquica e moral. Nenhum dos procedimentos utilizados oferece riscos a sua dignidade.

4. CONFIDENCIALIDADE DA PESQUISA: Todas as informações coletadas neste estudo são estritamente confidenciais. Seu nome não será mencionado em nenhum momento. Todos os dados serão analisados em conjunto, garantindo o caráter anônimo das informações. Os resultados poderão ser utilizados em eventos e publicações científicas.

5. BENEFÍCIOS: O benefício direto que você terá ao participar desta pesquisa será conhecer seus níveis de estresse e refletir sobre a prática do autocuidado no seu cotidiano. Sua

participação permitirá conhecer dados importantes acerca de fatores contribuintes para a adesão ao autocuidado por profissionais da saúde que cuidam diariamente de outras pessoas.

6. PAGAMENTO: Você não terá nenhum tipo de despesa por participar desta pesquisa. E nada será pago por sua participação. Entretanto, se você desejar, poderá ter acesso a cópias dos relatórios da pesquisa contendo os resultados do estudo. Para tanto, bastará entrar em contato com a pesquisadora responsável como segue:

Endereço da responsável pela pesquisa:
Nome: Prof. Dr. Eliseth Ribeiro Leão
Instituição: Instituto Israelita de Ensino e Pesquisa
Endereço: Av. Albert Einstein 627/700 – Bloco A – 2º subsolo
Telefones p/contato: 21511032 ou 973801412
ATENÇÃO: Para informar ocorrências irregulares ou para obter informações sobre os aspectos éticos do estudo, durante a sua participação, dirija-se ao:
Comitê de Ética em Pesquisa do Hospital Israelita Albert Einstein
Av. Albert Einstein, 627/700 - Bloco A, 2ºss
Morumbi - São Paulo - SP - 05652-000
Tel.: (55 11) 2151-3729
Fax.: (55 11) 2151-0273

CONSENTIMENTO DA PARTICIPAÇÃO COMO PARTICIPANTE

- Entendo que sou livre para aceitar ou recusar a participar e que posso retirar minha autorização e sair do estudo a qualquer momento. Ao assinar este Termo de Consentimento Informado, não estou abrindo mão de meus direitos legais. Receberei uma via assinada e datada deste Termo de Consentimento Livre e Esclarecido
- Tendo compreendido perfeitamente tudo o que me foi informado sobre a minha participação no mencionado estudo e estando consciente dos meus direitos, das minhas responsabilidades, dos riscos e dos benefícios que a minha participação implica, concordo em dele participar e para isso EU EXPRESSO O MEU CONSENTIMENTO SEM QUE PARA ISSO EU TENHA SIDO FORÇADO OU OBRIGADO.
- Além do consentimento, eu, afirmo não estar grávida ou amamentando no momento do início do estudo e me comprometo caso engravide, comunicar o pesquisador responsável.

Nome do participante:
____
Assinatura:
____
Data:____

Nome de quem aplicou o termo de consentimento livre e esclarecido:
____
Assinatura:
____
Data:____

Anexo XXI
Fórmula qualitativa e respectiva função dos ingredientes

natura

Código: **1082.18602.2**

Composição:

Código LIMS	Ingrediente - INCI	Função
455	AQUA	Veiculo
1773	GLYCERIN	Emoliente
1022	RICINUS COMMUNIS SEED OIL	Emoliente
15643	OLUS OIL	Emoliente
912	ISOHEXADECANE	Emoliente
768	CETEARYL ALCOHOL, CETEARYL ISONONANOATE, CETEARETH-20, CETEARETH-12, GLYCERYL STEARATE, GLYCERIN, CETYL PALMITATE	Emulsionante
599	DICAPRYLYL ETHER	Emoliente
1194	CAPRYLIC/CAPRIC TRIGLYCERIDE	Emoliente
10355	CANOLA OIL	Emoliente
957	BUTYROSPERMUM PARKII BUTTER	Emoliente
6993	SODIUM POLYACRYLATE	Doador de viscosidade
7267	GLYCOL DISTEARATE, GLYCERYL STEARATE, GLYCERYL OLEATE, AQUA, COCO-GLUCOSIDE, CITRIC ACID, LACTIC ACID, BENZOIC ACID, GLYCERIN	Opacificante
19593	PARFUM	Fragrancia
5395	DMDM HYDANTOIN	Conservante
7986	CAPRYLIC/CAPRIC TRIGLYCERIDE	Doador de viscosidade/emulsionante
1500	XANTHAN GUM	Controle de viscosidade
17802	HELIANTHUS ANNUUS SEED EXTRACT	Ativo
1213	TOCOPHERYL ACETATE	Antioxidante
7483	POLYGLYCERYL-3 CAPRYLATE	Ativo desodorante
4691	METHYLISOTHIAZOLINONE	Conservante
720	DISODIUM EDTA	sequestrante

Assinatura: Sandolin Data: 06/03/14
Pesquisador: Talita Sandolin

Assinatura: D.Okuta Data: 06/03/14
Revisor: Daniela Okuta

Anexo XXII
Ficha de Randomização

Número da Voluntária	Iniciais da voluntária	Grupo (1,2,3 OU 4)	Idade	Data de inclusão	Data da visita de 15 dias	Data de encerramento do estudo	Falha de seleção (registrar razão da falha)
01							
02							
03							
04							
05							
06							
07							
08							
09							
10							
11							

Protocolo Pesquisa H-Senses

Número da Voluntária	Iniciais da voluntária	Grupo (1,2,3 OU 4)	Idade	Data de inclusão	Data da visita de 15 dias	Data de encerramento do estudo	Falha de seleção (registrar razão da falha)
12							
13							
14							
15							
16							
17							
18							
19							
20							
21							
22							

Número da Voluntária	Iniciais da voluntária	Grupo (1,2,3 OU 4)	Idade	Data de inclusão	Data da visita de 15 dias	Data de encerramento do estudo	Falha de seleção (registrar razão da falha)
23							
24							
25							
26							
27							
28							
29							
30							
31							
32							
33							

Esta ficha de randomização conterá o número total de voluntárias selecionadas no estudo.
