# Supplementary material for: Stress, self-esteem and well-being among female health professionals: A randomized clinical trial on the impact of a self-care intervention mediated by the senses
Source: PLoS One. 2017 Feb 27;12(2):e0172455. doi: 10.1371/journal.pone.0172455 (PMC5328264; doi:10.1371/journal.pone.0172455)
Supplement: S3 Text — (PDF) [file pone.0172455.s003.pdf]

Protocol Registration Receipt
10/27/2014

H-Senses Project - Self Care Mediated by the Senses

This study is currently recruiting participants.
Verified by Eliseth Ribeiro Leao, Hospital Israelita Albert Einstein, October 2014

Sponsor:	Hospital Israelita Albert Einstein
Collaborators:	
Information provided by (Responsible Party):	Eliseth Ribeiro Leao, Hospital Israelita Albert Einstein
ClinicalTrials.gov Identifier:	

Purpose

Randomized clinical trial about self-care mediated by the senses in female health professionals and their influence on stress, self-esteem, mood states, life satisfaction and levels of salivary cortisol.

Condition	Intervention	Phase
Stress	monossensorial self care<br>multissensorial self care<br>bissensorial self care	N/A

Study Type: Interventional
Study Design: Treatment, Parallel Assignment, Open Label, Randomized, N/A
Official Title: H-SENSES PROJECT Self Care Mediated by the Senses: Assessing the Impact on Self-esteem, Stress and Well-being of Female Health Professionals.

Further study details as provided by Eliseth Ribeiro Leao, Hospital Israelita Albert Einstein:

Primary Outcome Measure:

- Change in perceived stress [Time Frame: baseline, 30 days, 60 days] [Designated as safety issue: No]

Lipp Stress Inventory and Symptoms of Stress List

Secondary Outcome Measures:

- Change in cortisol levels [Time Frame: baseline, 30 days, 60 days] [Designated as safety issue: No]
  Saliva will be collected to measure cortisol levels: 7-9 am

Other Pre-specified Outcome Measures:

- Changes in Satisfaction with life [Time Frame: baseline, 30 days, 60 days] [Designated as safety issue: No]
  Satisfaction With Life Scale
- Changes in Positive and Negative Affective [Time Frame: baseline, 30 days, 60 days] [Designated as safety issue: No]
  Positive and negative affect scale
- Change in perceived self-esteem [Time Frame: baseline, 30 days, 60 days] [Designated as safety issue: No]
  Rosemberg's self-eteem Scale

Estimated Enrollment: 240
Study Start Date: July 2014
Estimated Study Completion Date: March 2015
Primary Completion Date: October 2014

Arms	Assigned Interventions
No Intervention: Control<br>This control group will not receive an intervention	
Active Comparator: monossensorial self care<br>This group will carry out a daily moisturizing body care with an odorless moisturizer for 30 days	monossensorial self care<br>daily use of odorless body moisturizer
Active Comparator: bissensorial self care<br>This group will carry out a daily moisturizing body care with a moisturizer with fragance for 30 days	bissensorial self care<br>daily use of moisturizing body with fragrance
Active Comparator: multissensorial self care<br>This group will carry out a daily moisturizing body care with a moisturizer with fragance for 30 days, and will watch a video with music and images of nature during self-care.	multissensorial self care<br>daily use of moisturizing body with fragrance + audiovisual stimulus

The work environment of health professionals is commonly considered stressful, given that they need to deal with difficult situations that include pain, risk of death, the decisions that need to be fast and accurate, and conflicts generated in interpersonal relationships. There is an ongoing discussion that we need to care for those who care, however, there are few interventions studied and validated to effectively reduce the stress of these professionals that resonate in states of well-being. There is little room for discussion of this topic during the workday, as well as to learn techniques and measures for effective stress management. This study aims to explore the relationship between well-being, self-esteem and stress of women health professionals, and compare three self-care interventions mediated by the senses.

Eligibility

Ages Eligible for Study: 18 Years to 60 Years
Genders Eligible for Study: Female
Accepts healthy volunteers.

Inclusion Criteria:

- Professional healthcare;
- working in healthcare or administrative area in hospital
- Voluntary participation;
- Signed in term of free and informed consent.

Exclusion Criteria:

- Evaluation with relevant dermatological findings diagnosed by a dermatologist;
- Work at nighttime or in alternate periods (because of known chronobiological changes that would affect the study of cortisol, one of the outcomes considered in the study);
- who are breastfeeding;
- Be pregnant

Contacts and Locations

Contacts

Eliseth R Leão, PhD   +5511 996186489   eliseth.leao@einstein.br

Locations

Brazil

Hospital Israelita Albert Einstein   Recruiting
São Paulo, São Paulo, Brazil, 05652000
Contact: Eliseth R Leão, PhD   5511996186489   eliseth.leao@einstein.br

Investigators

Principal Investigator: Eliseth R Leão, PhD   Hospital Israelita Albert Einstein - Albert Einstein Jewish Institute for Education and Research

More Information

Publications:

Responsible Party: Eliseth Ribeiro Leao, PhD, Hospital Israelita Albert Einstein
Study ID Numbers: H-Senses
Health Authority: Brazil: Ethics Committee
