# Supplementary material for: Stress, self-esteem and well-being among female health professionals: A randomized clinical trial on the impact of a self-care intervention mediated by the senses
Source: PLoS One. 2017 Feb 27;12(2):e0172455. doi: 10.1371/journal.pone.0172455 (PMC5328264; doi:10.1371/journal.pone.0172455)
Supplement: S4 Text — (PDF) [file pone.0172455.s004.pdf]

PARECER CONSUBSTANCIADO DO CEP

DADOS DO PROJETO DE PESQUISA

Título da Pesquisa: H-Senses - Autocuidado mediado pelos sentidos: avaliação do impacto na autoestima, stress e bem-estar de mulheres profissionais de saúde.

Pesquisador: Eliseth Ribeiro Leão
Área Temática:
Versão: 2
CAAE: 26547314.3.0000.0071
Instituição Proponente: Hospital Israelita Albert Einstein-SP
Patrocinador Principal: NATURA INOVACAO E TECNOLOGIA DE PRODUTOS LTDA

DADOS DO PARECER

Número do Parecer: 622.662
Data da Relatoria: 08/04/2014

Apresentação do Projeto:
O ambiente de trabalho dos profissionais de saúde comumente é considerado estressante, tendo em vista que necessitam lidar com situações delicadas, que incluem sofrimento, risco de morte, tomadas de decisão que precisam ser rápidas e acuradas, além de conflitos gerados nas relações interpessoais. Há um discurso corrente de que necessitamos cuidar de quem cuida, entretanto, poucas são as intervenções estudadas e validadas para redução efetiva do stress desses profissionais.
O autocuidado precisa ser aprendido e, sobretudo ser vivenciado. Por outro lado, pouco se sabe sobre quais intervenções são eficazes e comprovadas cientificamente, quando se trata do manejo do stress, e de medidas que promovam a autoestima e o bem-estar de mulheres profissionais de saúde.
A negligência do cuidado de si parece decorrer da falta de tempo para alimentar-se adequadamente, cuidar-se física e esteticamente e, ainda, abdicar do uso do tempo para si em prol do trabalho. Parcela significativa dos profissionais da saúde é constituída por mulheres, que muitas vezes têm dupla ou tripla jornada de trabalho, o que torna essa situação ainda mais crítica.
Há algumas décadas o stress dos enfermeiros, bem como de outros profissionais da área da saúde

vem sendo estudado. Os níveis de stress, às vezes, são tão elevados que levam os profissionais a desistirem da profissão que abraçaram a conhecida síndrome de burnout. O desequilíbrio causado pelo stress, muitas vezes repercutem também em baixa autoestima e diminuição do bem-estar subjetivo.

As hipóteses deste estudo são:

1) Existe uma correlação inversa entre bem-estar e autoestima elevados e baixos níveis de stress.

2) O grupo da intervenção monossensorial terá resultados de maior redução do stress e melhora da autoestima e bem-estar do que o grupo controle.

2) O grupo da intervenção bissensorial terá resultados de maior redução do stress e melhora da autoestima e bem-estar do que o grupo controle e o grupo de intervenção monossensorial.

3) O grupo da intervenção multissensorial terá resultados de maior redução do stress e melhora da autoestima e bem-estar do que o grupo controle e os grupo de intervenção monossensorial e bissensorial.

4) O grupo da intervenção multissensorial terá melhores resultados de adesão ao autocuidado do que o grupo controle, grupo de intervenção monossensorial e bissensorial.

Objetivo da Pesquisa:

Este estudo tem como objetivos explorar as relações entre bem-estar, autoestima e stress de mulheres profissionais de saúde; comparar três intervenções de autocuidado mediada pelos sentidos (monossensorial - tato, bissensorial tato e olfato e multissensorial - tato, olfato, visão e audição) e conhecer a percepção das mesmas sobre essa experiência.

Avaliação dos Riscos e Benefícios:

RISCOS: Os riscos para este estudo são mínimos e podem estar relacionados à possível alergia ao hidratante utilizado e a perda de confidencialidade dos dados obtidos;

BENEFICIOS: possível redução do stress, promoção de bem-estar e melhora da autoestima e mulheres profissionais de saúde.

Comentários e Considerações sobre a Pesquisa:

Trata-se de um estudo clínico, controlado, aberto, randomizado, com abordagem mista (quantitativa e qualitativa).

Considerações sobre os Termos de apresentação obrigatória:

O TCLE está redigido em linguagem clara e acessível, utilizando-se das estratégias mais apropriadas à cultura, faixa etária, condição socioeconômica e autonomia dos convidados a

Endereço: Av. Albert Einstein 627 - 2ss
Bairro: Morumbi **CEP:** 05.652-000
UF: SP **Município:** SAO PAULO
Telefone: (11)2151-3729 **Fax:** (11)2151-0273 **E-mail:** cep@einstein.br

participar da pesquisa. Demais termos apresentados estão de acordo com a proposta do estudo.

Recomendações:

1-De acordo com a Res. CNS 466/12, o pesquisador deve apresentar ao CEP/Einstein os relatórios semestrais e o relatório final (através da Plataforma Brasil,ícone Notificação).

2-Em estudos prospectivos e intervencionistas solicita-se que o TCLE aprovado apresente o carimbo do CEP.

Conclusões ou Pendências e Lista de Inadequações:

Análise das Pendências Anteriores:

1- A amostra do projeto será composta por profissionais da saúde. Serão convidados
apenas profissionais da assistência ou todos serão elegíveis? Não está claro o critério para definição da amostra:
RESPOSTA DO PESQUISADOR: Foi acrescentado ao critério de inclusão que poderão ser profissionais de saúde assistenciais ou administrativos que atuem nas unidades descritas no critério. Ambos sofrem influência do estresse ocupacional relacionado à atuação em instituições de saúde, na perspectiva organizacional. Como a divulgação será feita institucionalmente serão recrutadas voluntárias que desejem participar do estudo que atendam aos critérios de inclusão descritos no estudo que foram revisados. PENDENCIA ATENDIDA

2-Por que serão excluídos voluntários que trabalham no noturno e em dias alternados, os quais podem ter um nível maior de stress?
RESPOSTA DO PESQUISADOR: Primeiramente, os trabalhadores do noturno não necessariamente apresentam maiores índices de estresse. A literatura aponta que as causas do estresse estão mais relacionadas a outros fatores organizacionais e ligadas à natureza do trabalho desenvolvido como apresentado no estado da arte do presente projeto, do que propriamente relacionado ao turno de trabalho. Trata-se de uma exclusão clássica nos estudos que envolvem estresse, simplesmente por não permitir a comparação do turno noturno com o diurno, em virtude das alterações do ciclo sonovigília, demonstrados em estudos cronobiológicos e que podem interferir nos exames de cortisol salivar previstos, uma vez que estes são dependentes do ciclo circadiano. PENDENCIA ATENDIDA

3-Se só serão incluídas voluntárias que não utilizam medicamentos, assumimos que as

Endereço: Av. Albert Einstein 627 - 2ss
Bairro: Morumbi **CEP:** 05.652-000
UF: SP **Município:** SAO PAULO
Telefone: (11)2151-3729 **Fax:** (11)2151-0273 **E-mail:** cep@einstein.br

funcionárias sob alto stress, que em geral, estão sob tratamento, não farão parte da amostra. Isso não pode conduzir a uma amostra exclusivamente composta por voluntários com baixo stress?
RESPOSTA DO PESQUISADOR: Os estudos apresentados na introdução do projeto indicam elevada prevalência de estados de estresse na população-alvo, o que minimiza a possibilidade de uma amostra exclusiva por voluntários com baixo stress. Ainda que isso ocorresse, outros desfechos estão sendo analisados em relação ao objeto do estudo (autocuidado) que podem estar presentes, mesmo em indivíduos com baixo grau de estresse que alguns indivíduos venham a apresentar, sem comprometer a avaliação dos achados. PENDENCIA ATENDIDA

4-o nível de stress será um dos itens avaliados como desfecho, contudo, a presença de stress não foi caracterizada como um critério de inclusão. Por favor, avaliar. Se considerado como critério de inclusão, por favor, reconsiderar metodologia das visitas, para definição de como a amostra será recrutada.
RESPOSTA DO PESQUISADOR: O nível de estresse não será caracterizado como critério de inclusão, uma vez que todos os níveis de estresse apresentados serão avaliados em relação ao autocuidado executado, bem como a associação com os desfechos secundários. Como não se trata de um único desfecho, as demais variáveis são igualmente importantes para a compreensão do autocuidado mediado pelos sentidos e também serão conhecidas em relação aos níveis baixos de estresse que houverem. PENDENCIA ATENDIDA

5-Especificar se o braço controle será submetido às mesmas avaliações dos braços com
proposta de cuidado.
RESPOSTA DO PESQUISADOR: Como já descrito no segundo parágrafo da página 11 do arquivo original: “O grupo controle será submetido às mesmas avaliações dos grupos de intervenção, com exceção do preenchimento do diário e da questão sobre a experiência de autocuidado”.
Tal informação, por ajustes no projeto encontra-se agora descrita na página 13 em Medidas de Avaliação – Variáveis de estudo. PENDENCIA ATENDIDA

6- Descrever na metodologia as variáveis que serão coletadas, de acordo com os instrumentos que serão utilizados, a fim de definir os desfechos e análises a serem executadas.
RESPOSTA DO PESQUISADOR: As variáveis que serão coletadas já se encontravam descritas no parágrafo 4º da página 10:“As variáveis estudadas serão: cortisol salivar, nível de stress percebido, autoestima, bem-estar subjetivo, verificadas pré intervenção, após 15 dias de intervenção e após o

período previsto para a intervenção (30 dias), mediante a aplicação de questionários, que serão complementados por desenhos feitos pelas participantes. Uma avaliação da percepção da experiência será realizada ao final do estudo”
Nas páginas 11, 15 estavam descritos todos os instrumentos relacionados às medidas a serem realizadas, bem como as análises (interpretação) a serem realizadas.
Com ajustes de texto todas as informações encontram-se descritas a partir da página 13 onde acrescentamos para facilitar ao revisor o subtítulo: Medidas de Avaliação – Variáveis de estudo. PENDENCIA ATENDIDA

7-Como será o cronograma de visitas? Não está claro o que será aceito em cada visita. Tanto na metodologia, como no TCLE.
Novas informações foram acrescentadas. PENDENCIA ATENDIDA

8- o uso de outros hidratantes é um critério de exclusão? O uso de outros produtos não é viés? Essa abordagem será apresentada no TCLE?
RESPOSTA DO PESQUISADOR: A utilização de outros hidratantes não será considerado critério de exclusão, todavia será orientado a suspensão do uso durante o período do estudo. Esta informação consta das orientações que serão fornecidas às participantes conforme grupo de intervenção para o qual for randomizada, conforme anexos III e VI e foi incluído também no TCLE. PENDENCIA ATENDIDA

9-Explicar como será feita a análise estatística dos dados de acordo com objetivos, hipótese e variáveis coletadas.
RESPOSTA DO PESQUISADOR: Os dados serão submetidos à análise estatística descritiva e inferencial, conforme descrição no projeto fornecida pelo serviço de estatística do IIEPAE. As análises qualitativas também se encontram descritas no método (página 19) já informadas na versão 1. PENDENCIA ATENDIDA

10-Pendencias no TCLE: informações necessárias foram acrescentas e nova versão anexada. PENDENCIA ATENDIDA

APÓS ATENDIMENTO AS PENDENCIAS APONTADAS NO PARECER ANTERIOR, OS SEGUINTES DOCUMENTOS FORAM APROVADOS:

1-Protocolo de Pesquisa Versão 2 de 27 de março de 2014;

2-Termo de Consentimento Livre e Esclarecido - Versão 3 de 22 de Abril de 2014.

Situação do Parecer:

Aprovado

Necessita Apreciação da CONEP:

Não

Considerações Finais a critério do CEP:

DOCUMENTAÇÃO APROVADA PELO CEP DO HOSPITAL ISRAELITA ALBERT EINSTEIN EM REUNIÃO REALIZADA EM 08/04/2014.

SAO PAULO, 23 de Abril de 2014

Assinador por:
Fabio Pires de Souza Santos
(Coordenador)
