# Supplementary material for: Stress, self-esteem and well-being among female health professionals: A randomized clinical trial on the impact of a self-care intervention mediated by the senses
Source: PLoS One. 2017 Feb 27;12(2):e0172455. doi: 10.1371/journal.pone.0172455 (PMC5328264; doi:10.1371/journal.pone.0172455)
Supplement: S5 Text — (PDF) [file pone.0172455.s005.pdf]

H-Senses Project – Self-care mediated by the senses

1. Study objectives and target problems.

The objectives of this study include exploring potential relationships among well-being, self-esteem and stress in female healthcare professionals; comparing three self-care interventions mediated by the senses (mono-sensorial – touch, bi-sensorial – touch and smell, and multi-sensorial – touch, smell, sight, and sound); and understanding how these women perceive the experience.

We start from the premise that the quality of healthcare depends on professional competence, and on the good health conditions of the care giver. In this context, self-care is crucial to those who intend to care for others. Nevertheless, we have observed, both in the clinical and academic settings, that experienced and unexperienced professionals often have little awareness of this fact and rarely adopt healthy habits.

The work environment of healthcare professionals generates a number of stressful situations including the need for fast and accurate decision-making, interpersonal conflicts, and coping with moments of life or death for many patients. The health system should clearly prioritize caring for those who care, however, few studies have focused on the validation of interventions that reduce the stress and improve welfare of these professionals. During their work journeys health care professionals have little time available to discuss the theme, or to learn effective stress-management techniques.

Self-care represents the central point of Dorothea Orem’s theory, which is taught in nursing undergraduate programs and adopted in the Systematization of Nursing Assistance in many institutions to guide patient self-care. Nevertheless, a gap often forms between the theory and practice of healthcare. We observe that many professionals do not take good care of themselves. They often develop poor eating habits, do not take time off, among other issues that may erode the confidence and adhesion of patients regarding their orientations, especially those involving self-care.

Self-care theory involves related concepts such as self-care, self-care action, and therapeutic demand for self-care. The first concept consists of the activity that individuals engage in their own benefit, to improve their lives, health and welfare. Self-care action refers to the ability that humans have to engage in self-care. Finally, the therapeutic demand for self-care include the factors that must be approached, controlled and modified in an individual because they affect his or her organism and human development (Orem, 1995).

Self-care must be learned and, most of all, lived. However, little is known about which interventions effectively help in stress management, as well as self-esteem and welfare promotion in female healthcare professionals. Stress among nurses and other healthcare professionals has been the target of many studies for decades. It may reach unbearable levels that make professionals give up on their careers, a condition known as burnout syndrome. At this stage, a study shows, nurses will show high levels of emotional exhaustion, depersonalization, and low levels of professional fulfillment. The same authors showed the following associations: emotional exhaustion and frequently performing very fast tasks (p=0.039), and earning salaries that are incompatible with the efforts required by the job (p=0.016). Among study participants, 19% had a high propensity to develop the syndrome in at least two of the three dimensions evaluated by the instrument (Galindo et al; 2012).

A demanding job, among other issues, promotes stress in healthcare professionals and results in physical and psychological symptoms: nervousness, nightmares, irritability, headaches, insomnia, and gastrointestinal problems (Tsai, Liu, 2012). The unbalance caused by stress often reduces self-esteem and the subjective feeling of welfare.

Healthcare places a natural focus on assisting patients, whereas professional self-care remains in second plan. This pattern emerges from the lack of time for adequate eating, exercising, and for taking care of one's own appearance. Women constitute a large portion of healthcare workers, who often have double or triple work shifts, making the situation ever more critical.

We seem to imagine, collectively, that the female healthcare professional, the educator-caregiver-human never becomes ill or gets tired. Thus, the caregiver often forgets to care for herself. Researchers point to a necessary rescue of self-care to enable the full exercise of the healthcare profession and personal fulfillment (Baggio, Formaggio; 2007).

Investigating self-care strategies that might be learned and incorporated into the daily lives of healthcare professionals represents a crucial step for the improvement of healthcare in general. In this sense, this study seeks alignment with the concept of well-being/being well developed by our partner company Natura. We will evaluate to what extent we could promote a "harmonious and pleasant relation of an individual with his- or herself, with his or her body, as well as an empathic, successful, pleasant relation of an individual with others, with nature, with the whole".

We perceive the world through the senses of touch, sound, sight, smell and taste. Thus, in this study, we propose to assess a body hydration intervention in relation to the senses, in three modes: mono-sensorial (touch), bi-sensorial (touch and smell) and multi-sensorial (smell, touch, sound and sight). In doing so, we will verify the impact of the intervention on stress levels, subjective welfare (i.e. satisfaction with the individual's own life) and self-esteem of healthcare professionals.

Our working hypothesis include:

1) Welfare and self-esteem are inversely correlated with stress.
2) The mono-sensorial group will display greater stress reduction and welfare/self-esteem improvement than the control group.
3) The bi-sensorial group will display greater stress reduction and welfare/self-esteem improvement than the control and mono-sensorial groups.
4) The multi-sensorial group will display greater stress reduction and welfare/self-esteem improvement than the control, mono- and bi-sensorial groups.
5) The multi-sensorial group will have the highest degree of self-care compliance of any other group.

2. Rational and expected impacts

Stress represents a current, trendy term, widely present in the literature, specifically regarding its effects on health care and nursing professionals. The increase in professional activities targeting stress management has not found a parallel in the increase in compatible research. Most existing programs have not been evaluated properly because of two types of problems. First, most interventions have not been systematically assessed and, second, when a systematic evaluation does occur, the study plans are often marked by serious methodological flaws. Data generation remains slow and covering only a minority of existing stress prevention activities. Finally, this type of research assessment continues to have “a post-hoc bias for the individual” (Kompier, Kristensen; 2003).

Some studies briefly address the questions of stress reduction and control of other symptoms by means of relaxation techniques, such as massage, music and the use of mental images. However, the current study, as it has been designed, provides a novel insight into stress management.

Expected results:

- We expect that at least one intervention should prove effective in reducing stress and promoting self-esteem and welfare;
- We are testing female healthcare workers, but positive results, mediated by human senses, could apply equally well to other women in the same age range;
- If tests yield the expected results, further studies may focus on distinct age ranges and in male professionals.

Project deliverables:

- Scientific publications and exposure in scientific events.

3. Case and method

Study design

This will be an open clinical trial with randomized allocation of participants to groups and a mix of quantitative and qualitative approaches.

The interventions will last thirty days. Data will be collected in the beginning of the study (baseline), after 15 days, at the end of interventions, day 30, and after a follow-up period of 30 days, i.e., 60 days after the beginning of the study.

After an explanation of study objectives and validation of inclusion/exclusion criteria (Appendix II), women who chose to participate will read and confirm their decision by signing the informed consent form ICF (Appendix XX). Participants will receive specific orientations in accordance with the group to which they were randomly assigned. We will use the Research Randomizer® software to assign the 60 participants to four groups:

- Group 1) Control;
- Group 2) Mono-sensorial intervention (touch): daily body hydration with scentless cream;
- Group 3) Bi-sensorial intervention (touch and smell): daily body hydration with scented cream;
- Group 4) Multi-sensorial intervention (touch, sound, sight and smell): daily body hydration with scented cream, after watching brief audiovisual materials with nature scenes.

Ethical Aspects

Participants will receive a code number to ensure the privacy of their responses to interventions. Data collection forms and other study documents will be safely stored in a locker and electronic files will be kept in keyword-protected computers and folders to which only researchers will have access. The project will be submitted to the Institutional Research Ethics Committee, and participation in the study will depend on participants agreeing to read and sign an informed consent form.

In conformity with Resolution 466/1, National Health Council, participants will be informed about research contents and goals and will be able to freely leave the study at any moment.

Study participants

After approval by the Ethics Committee, the research will be announced and patient enrollment into each group will begin. The enrollment period will end when each group includes 60 female healthcare professionals from the Sociedade Beneficente Brasileira Israelita Albert Einstein (SBIBAE), totaling 240 women. Sample size was defined by convenience with no theoretical calculation, because no a priori estimates exist on expected results.

The Internal Institutional Communication Area will recruit participants via the intranet, e-mail messages, posters, and boards with great visibility by potential participants (Appendix I).

Inclusion criteria:

- Female healthcare professional;
- Adult between 18 and 60 years of age;
- Assistance or administrative role in the institution;
- Voluntary participation;
- Signed the informed consent form.

Exclusion criteria:

- Relevant skin condition diagnosed by dermatologist;
- Night shift or alternate shift workers (circadian fluctuations could affect cortisol, a marker for this study);
- Lactating mothers;
- Pregnant women.

Current use of hydrating creams will not be considered an exclusion criterion, however, participants will be instructed to interrupt use during the study. The same procedure will apply to stress-relief drugs. The hydrating cream to be used by Groups II, III and IV will be provided in the first visit.

Evaluations of each participant will be conducted at the Instituto Israelita de Ensino e Pesquisa Albert Einstein do Hospital Israelita Albert Einstein (IIEP), upon appointment made by the research coordinator at the Clinical Research Center of IIEP-SBIBAE.

In the first contact with potential participants, the inclusion and exclusion criteria check-list (Appendix II) and the informed consent form (Appendix XX) will be used to decide participant enrollment in the study.
We will run a pilot test with 10 volunteers to evaluate potential adjustments to study operational procedures.

Experimental intervention

Women in groups III and IV will receive hydrating body cream, and the same base without scent will be given to group II women. Containers will have no reference to Natura or to any other brand name. Product formula code (1082.18602.2), list of ingredients and ingredient functions are shown in Appendix XXI.

Mode of use of hydrating cream: the cream will be applied to the whole body except to the face, daily. Each participant will select a time of day to perform this application, for 30 days. Participants will make daily entries into a diary to record product use.

In groups II and III, cream application will consist of soft self-massage for absorption of the hydrating product. Participants in this group will receive specific orientations presented in Appendix III.

Group IV participants, before and after the self-care activity, will watch an audiovisual material made available in a tablet given to them in the beginning of the study. These participants will sign a receipt and a form guaranteeing return of the equipment by the end of the study (Appendix IV). A PowerPoint presentation will be available in the tablet containing a sequence of nature scenes and music lasting two minutes and forty seconds. The tablet will have no other function available other than reproducing selected images. Images were selected from previously mentioned studies (Ulrich, 1984; Marcus, Barnes; 1995), and from unpublished semiotic work seeking chromatic, verbal, visual, tactile and olfactory signs in cotton developed by Natura consultants aiming at finding connecting sighs for the hydrating cream. The sequence of images comprises 17 photographs of flowers and landscapes shown in Appendix V. Group IV orientations can be found in Appendix VI.

The aforementioned semiotic study also provided the basis for the musical selection, the traditional Irish song *Sweet Portaferry* (McGrath,1992), executed by Eve Watters in the CD Quiet Visit – Celtic Harp (2005). The melody is conducted in a harp, in E flat major, slow tempo (35 beats/minute), 6/8 compass, with a warm and melancholic character. This song (Appendix VII) lies within the musical parameters for relaxation (Nilsson 2010; Lai, Li; 2011), it is loaded with harp sounds, and signs that are similar to those pertaining to cotton, such as smoothness, coziness, and care (Ganzini, Rakoski, Cohn, Mularski; 2013)

If a participant develops allergies or other problems during the course of the study, she will be evaluated by a dermatologist who will be on call during the study, and if necessary she will be sent to the appropriate medical institution covered by her health insurance, and an Adverse Effect Notification will be filled out (Appendix VIII), which will be sent to the Ethics Committee.

By the end of the study, the sponsoring company Natura will collect left-over samples and dispose of them appropriately at the company's residue center.

Study variables

The main variables evaluated will include salivary cortisol, perceived stress level, self-esteem, subjective welfare, symbolic experience, daily and global perceptions of the self-care experience, and adhesion to self-care after the study end.

Control group women will be submitted to the same evaluations and procedures, except for the required daily entries in the diary. Control and intervention groups will be evaluated at the days 0 (baseline), 15, 30 (final day of the interventions) and 60 (follow-up). All participants, regardless of group, will be evaluated by a dermatologist at the beginning and end of the study.

The frequencies for each specific evaluation are shown in Appendix IX.

In the first study visit, baseline, participants will fill out a form with demographic data including age, marital status, number of children, religion, educational level, area of work, time since graduation, time working in the same area, work hours, income, existence of financial, physical or emotional difficulties, frequency of use of self-care activities. This information will provide a broad characterization of the sample and will allow us to verify the homogeneity of randomly formed groups (Appendix X). The registration of randomized participants will be presented in Appendix XXII.

Study participants will have their salivary cortisol samples collected once per visit at the clinical lab of the host institution between 8 and 9 am. Cortisol is a steroid hormone produced in the cortical region of the adrenal glands in response to stress. Hormone levels will serve as a quantitative measure of stress. Salivary cortisol displays the same circadian rhythm as serum cortisol, thus, levels are more elevated at approximately 8 am and at their lowest between 0 and 4 am. Salivary cortisol concentrations depend on the free fraction of cortisol, and reflect blood cortisol. However, extremely high serum levels may lead to disproportional levels in the saliva. The method we chose to use to measure cortisol is simple, clean, non-invasive, painless, safe and does not cause stress. Sample collection is made in plastic tubes with special cotton. Samples are stable at room temperature for one week (Castro; 2003).

Participants will fill out the following forms:

- LIPP Stress Symptom Inventory, LSSI (Appendix XI)
- Adult Stress Symptom List, SSL (Appendix XII)
- Self-esteem scale (Appendix XIII)

- Life satisfaction scale (Appendix XIV)
- Positive and negative effect scale (Appendix XV)
- Group 2 and 3 diaries (Appendix XVI)
- Group 4 diary (Appendix XVII)

Finally, a qualitative analysis will be conducted to assess participant experience.

Diaries (Appendices XVI and XVII)
Participants will receive a brochure-like diary where they will register the date of their last period and the date of the first day of the cycle (if the participant has her period during the study). They will also make the following daily entries: date, time when they began self-care, time when they finished self-care, emotions, and clinical signs if any resulting from the experience during that day or 30 days after the end of the study. As a global assessment of the experience, the participants will be asked to answer the following open question: “How did you feel about the self-care experience during this month?”

The Group 4 diary will be identical to the others except for containing 3 specific questions about the audiovisual material and a reminder that the tablet should be returned.

DRAWING (Appendix XVIII)
As an assessment of the participants’ symbolic experience, it will be asked at the beginning and at the end of the study that each one of them make a drawing representing the following question: “How do you feel? (use the last week as a reference)”. To this end, participants will receive color pencils and Letter size paper.

FOLLOW-UP (Appendix XIX)
The evaluation variables (except the diaries) will be measured again 30 days after the last day of the study period, in addition to a question to assess adhesion to self-care: “Have you been exercising self-care? If so, how?

Data analysis

Demographic variables and specific study variables will be assessed at the baseline visit, and at days 15, 30 and 60. These variables will be described by absolute frequencies and percentages, or with summary measures such as averages, medians, standard deviations, interquartile intervals, and minimum and maximum values.
Instrument results will be evaluated in categories, defined during the studies that validated the instruments to facilitate interpretation.

The potential relation among welfare, self-esteem and stress levels will be assessed in double-entry tables with Pearson’s Chi-square test.

Comparisons among groups regarding stress, welfare and self-esteem will be performed with general estimate equations, focusing on the correlation among the averages from different time points, or with the use of non-parametric techniques of analysis of variance for repeat measurements.

The qualitative findings that complement the clinical survey will contemplate the analyses of drawings as to the colors used classified according to the visible spectrum as hot (red, orange, yellow, brown and intermediate tones) or cold (green, blue, grey, violet and intermediate tones). Colors and objects will be analyzed with the help of the Dictionary of Symbols (Chevalier, Gheerbrant, 2001), used in a previous work (Leão, 2005).

Qualitative answers will be submitted to content analysis, according to Bardin (1977), a group of analytical techniques used in communications to describe the content of messages.

Appendix only Portuguese version

References

Baggio MA, Formaggio FM. Profissional de enfermagem: compreendendoo autocuidado. Revista Gaúcha de Enfermagem 2007;28(2):233-41.

Bardin L. Análise de conteúdo. Lisboa: Edições 70; 1977.

Castro M, Moreira AC. Análise crítica do cortisol salivar na avaliação do eixo hipotálamo-hipófise-adrenal. Arq Bras Endocrinol Metab 2003; 47(4): 358-367.

Chevalier J, Gheerbrant A. Dicionário de Símbolos. 16ª ed. Rio de Janeiro: José Olympio; 2001.

Galindo SG, Moreno IM, Muñoz JG. Prevalencia de Ansiedad y Depresión en una Población de Estudiantes Universitarios: Factores Académicos y Sociofamiliares Asociados. Cínica y Salud 2009, 20(2): 177-187.

Ganzini L, Rakoski A, Cohn S, Mularki RA. Family members’ views on the benefits of harp music vigils for terminally-ill or dying loved ones. Palliat Support Care 2013; 16:1-4.

Kompier M A J, Kristensen TS. As intervenções em stress organizacional. Cad. psicol. soc. trab. 2003 periódico na Internet. citado 2012 Out 19. Disponível em: http://www.revistasusp.sibi.usp.br/scielo.php?script=sci_arttext&pid=S15163717200300020000 4&lng=pt.

Lai HL, Li YM. The effect of musico n biochemical markers and self-perceived stress among first-line nurses: a randomized controlled crossover trial. J Adv Nurs 2011. 67(11): 2414-24.

Marcus CC, BarnesM. Gardens in healthcare facilities: uses, therapeutic benefits, and design recommendations.California: The Center for Health Design; 1995.

McGrath M. My gentle harp: Irish fold tunes. Dublin: Stepaside; 1992.

Nilsson U. Music: a nursing intervention. European J Cardiovasc Nurs 2010; 10:73-4.

Orem DE.Nursing – concepts of practice. 5 ed. St. Louis: Mosby; 1995.

YC Tsai, Liu CH. Factors and symptoms associated with work stress and health-promoting lifestyles among hospital staff: a pilot study in Taiwan. . BMC Health Services Research 2012; 12:199.

Ulrich R. View through a window may influence recovery from surgery. Science1984; 224: 420-21.
